# Supplementary material for: A Middle Pleistocene Denisovan molar from the Annamite Chain of northern Laos
Source: Nat Commun. 2022 May 17;13:2557. doi: 10.1038/s41467-022-29923-z (PMC9114389; doi:10.1038/s41467-022-29923-z)
Supplement: Supplementary file 1 — Supplementary Information [file 41467_2022_29923_MOESM1_ESM.pdf]

Supplementary Information for

A Middle Pleistocene Denisovan molar from the Annamite Chain of northern Laos

This PDF file includes:

Supplementary Materials
Supplementary Discussion
Supplementary Figures 1 to 14
Supplementary Tables 1 to 17
Supplementary References

Supplementary Discussion
Geology

Cobra cave is located in Huà Pan province, northeastern Laos, ~260 km NNE of Vientiane. The tower karst in which the cave is formed is situated on the southeastern side of P'ou Loi Mountain and is 20 m north of the road from Na Phit to Muang Kout (Figure 1a). The 66-m karstic network has two levels with a number of minor galleries (Supplementary Figure 1). The main entrance overlooks the road and the alluvial plain at a height of 34 m. Another cave (Tam Ngu Hao 1, Balcony Cave) is located ~10 m below the main entrance to Cobra Cave and has yielded numerous Hoabinhian stone tools as yet unpublished although similar to those identified in the nearby Tam Hang rock shelter[1,2]. The lithic assemblage at Balcony Cave is mixed with fragments of freshwater shells and some terrestrial molluscs on two occupation levels, the oldest of which dates to 5000 years BP. Cobra Cave was identified for prospecting in December 2018.

During multiple visits to the cave between 2018-2020, the geology and sediment sequence was studied in detail and samples were taken for micromorphology, dating and fossil recovery. The basement of the site consists of a Palaeozoic granite and diorite covered with a folded widespread sedimentary formation. This formation is weakly metamorphic and characterized by grey to yellow pelites, siltites and arkosic sandstones, attributed primarily by Saurin[3] to the Silurian, possibly with some rock of Devonian origin. This clastic episode is capped by a thick limestone formation attributed to an interval between the Late Carboniferous (Moscovian) and the Permian[3,4]. The karstic limestone formation hosting the cave consists of a pluri-decametric massive sparitic dark-grey carbonate without marl intercalation containing place-to-place crinoidal remains and tetracoral fossils.

The sediments that contain the teeth and bone fragments infill a chamber located close to the main entrance, and we focus our study on this cave chamber. The upper part of the deposit is well preserved; a significant amount of sediment has been eroded from the middle part of the section, although an appreciable quantity of sediment has been preserved and is plastered to the wall of the cave (Figure 1b). These partially eroded sediments consist of lower and upper facies, representing two phases of sediment accumulation separated by an unknown period of time and marked by an erosional surface (see dotted line in Figure 1b). The lower facies (Lithological Unit 1, LU1) is well preserved and consists of brown arenitic silty clays and locally poorly cemented calcite along the wall and is without vertebrate remains (Figure 1e). The upper facies (Lithological Unit 2, LU2) is a mixed breccia/conglomerate unit in an arenitic-dominated matrix that is cemented by calcite (Figure 1d). Clast size ranges from very fine gravel up to pebbles and cobbles. Large cobbles do not exceed 20 cm on the long axis and are found primarily towards the top of the sequence. The maturity of these gravel clasts is highly variable, ranging from moderately angular to sub-rounded and occasionally rounded, a function of the varying sources of these coarse components and their variable residence times. They are formed of limestone originating from the calcareous substratum (intra-karstic origin). The extra-karstic clasts are very angular to sub-

rounded in shape, according to their petrography. These are made of quartzite, milky quartz, sandstone, pelite and some rare, altered granite pebbles, all of which originate from the basement bedrock outcropping everywhere in the catchment area of the site, but are currently exposed at a lower altitude of the studied cave due to downcutting/uplift. Some volcanic pebbles close to dolerite have been recognized. According to the imbrication of pebbles, the flow current was directed from the inside to the outside of the cave (from NW to SE).

This breccia/conglomerate unit displays a characteristic coarsening up negative sequence from the level -180 to the top (Figure 1b). A thick flowstone has formed on the highest part of the sequence (Figure 1c). A detailed examination showed the occurrence of two episodes of flowstone formation. A 10 cm–thick flowstone covers most of the surface, leaving only the largest pebbles exposed above the calcite, and a second very thin (~5 mm) flowstone that is not easy to trace terminates the sequence.

The depositional history of the stratigraphic sequence can be summarised thus: the lower facies (LU1) was deposited under relatively low energy hydrological conditions, possibly in a partially or totally drowned gallery, although we do not have a complete understanding of this phase of aggradation. The lack of discontinuities, small sequences or laminae limits any interpretation of the dynamics or duration of deposition of LU1. The coarsening-up negative sequence of the upper facies (LU2) is consistent with an increase of fluvial dynamics, usually associated with a flood event, occurring in the cave following heavy rains and potentially including super-saturated debris flows as well as fluvial deposits. The corollary of this is that LU2 was most likely deposited during a single event over the course of days or even hours. We record intra-karstic and extra-karstic clasts, as well as bones and teeth, mobilized and deposited together in the same sequence. As is the case in many karstic systems, a significant proportion of the sediments, bones and teeth derive from fossil-bearing sediments removed from higher up the karstic networks. This implies that LU1 and LU2 represent two distinct aggradational phases separated in time and marked by a modification of the karst hydrographic network.

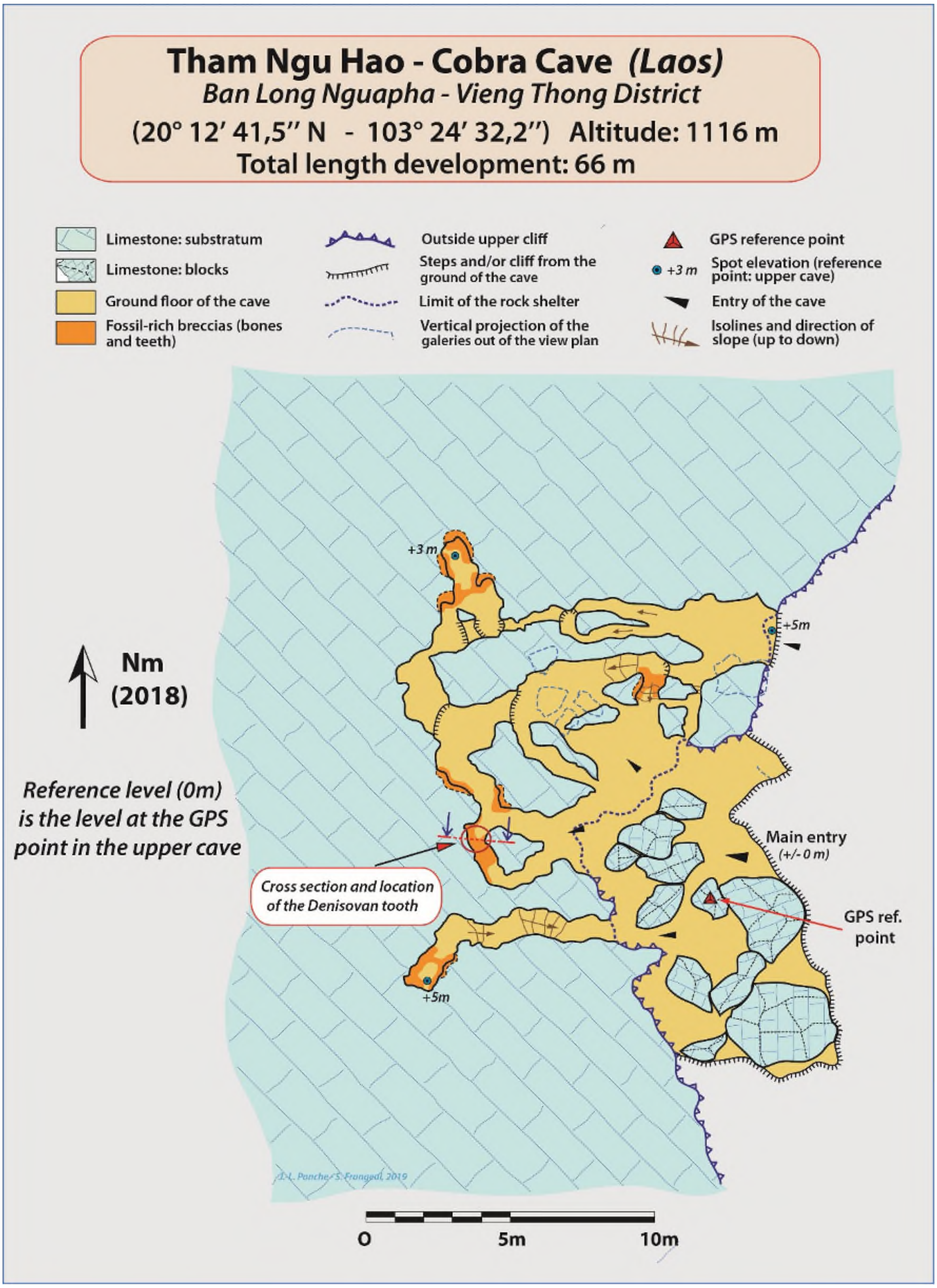

Supplementary Figure 1. Location of Tam Ngu Hao 2 (Cobra Cave) and its surrounding karstic network.

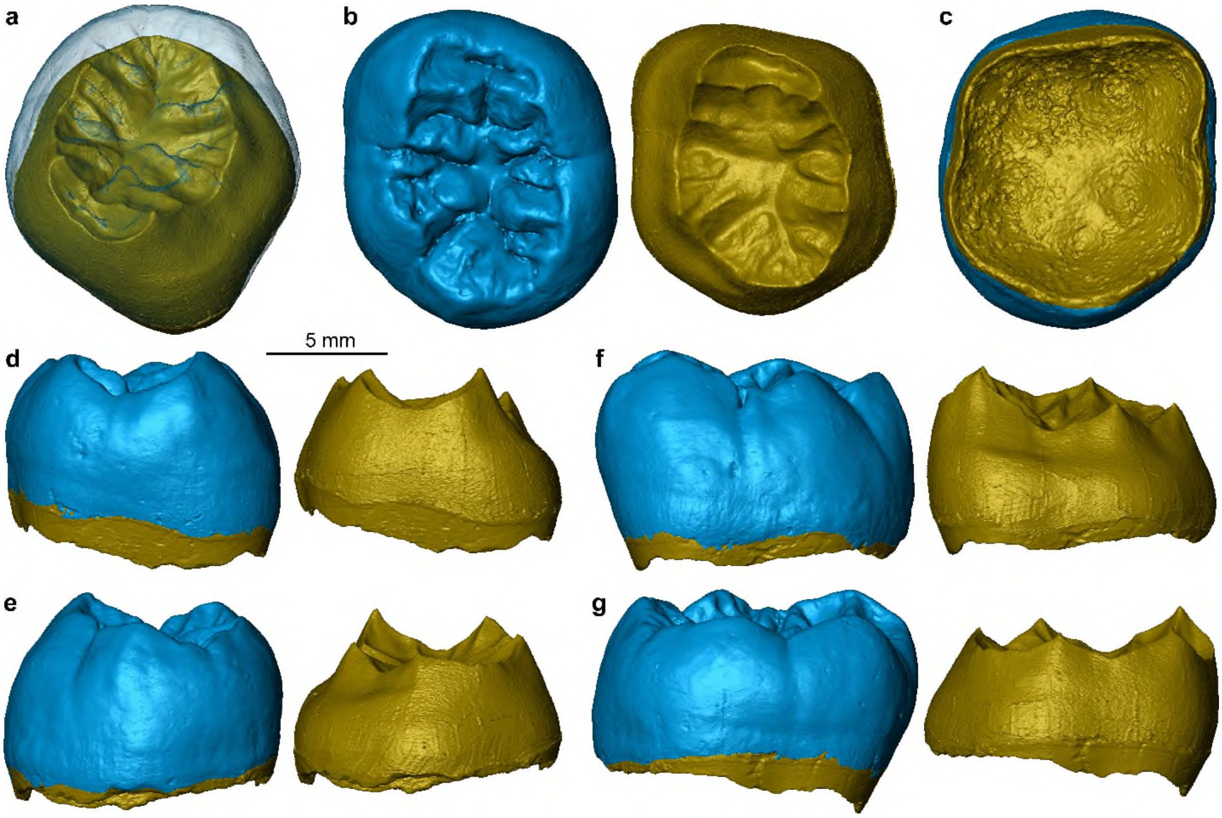

Supplementary Figure 2. Virtual rendering of TNH2-1. **a**, TNH2-1 shown in tilted view with the enamel shown in semi-transparency and the dentine below. **b-g**, the outer enamel surface (blue) and dentine (yellow) illustrated in occlusal (**b**), inferior (**c**), mesial (**d**), distal (**e**), buccal (**f**), and lingual (**g**) views.

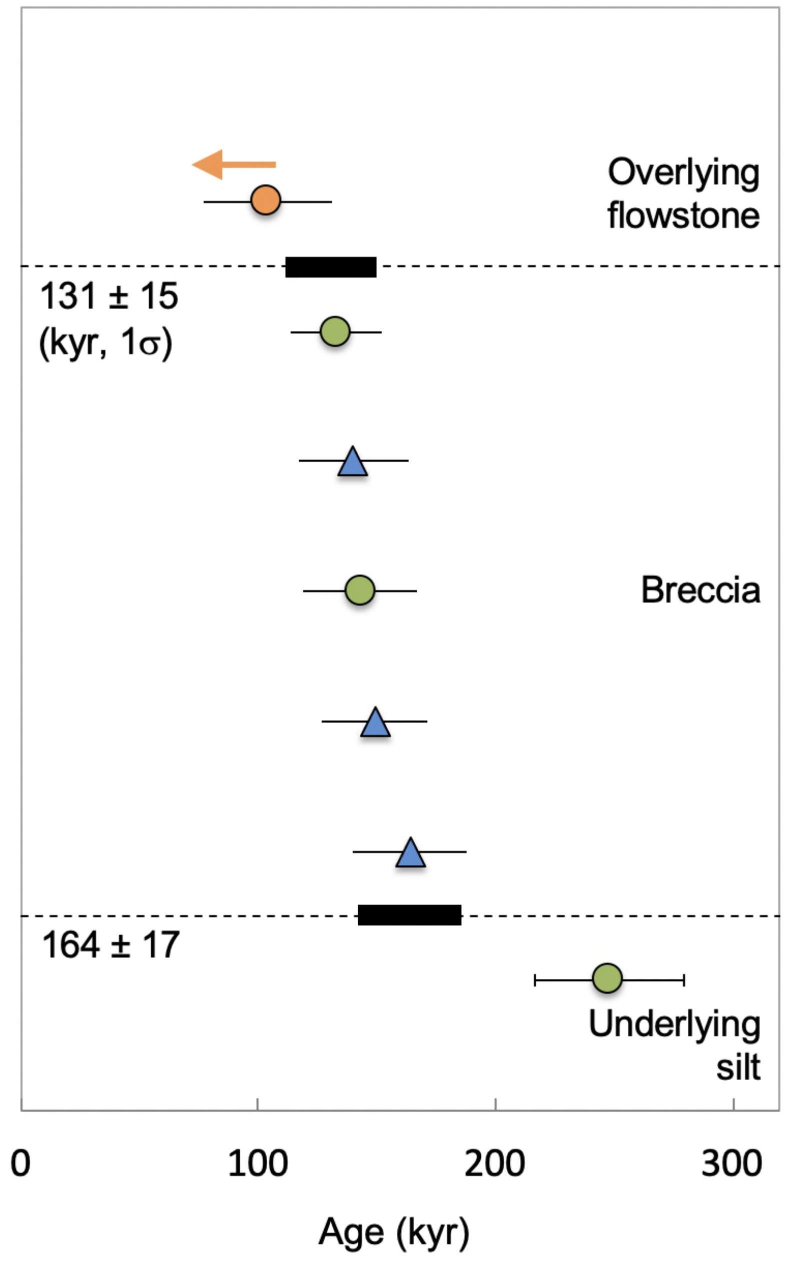

Supplementary Figure 3. Bayesian modelling of all independent age estimates. U-series dating of flowstone (orange circle) presented at 2 sigma error margins, pIR-IRSL dating of breccia matrix (green circles) presented at 1 sigma error margins, and US-ESR dating of mammalian teeth (blue triangles) from the fossiliferous breccia presented at 2 sigma error margins. The black dashed lines represent the modelled age ranges for the boundaries between each unit. The breccia containing the tooth has a modelled age range of 164-131 kyr.

a **b**

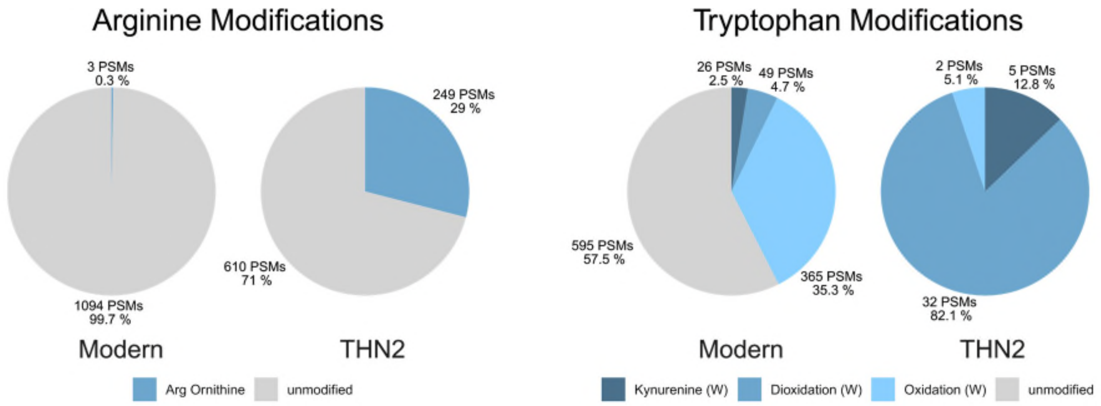
Arginine Modifications
3 PSMs 0.3 %
1094 PSMs 99.7 %
249 PSMs 29 %
610 PSMs 71 %
Modern
THN2
Arg Ornithine
unmodified
Tryptophan Modifications
26 PSMs 2.5 %
49 PSMs 4.7 %
595 PSMs 57.5 %
365 PSMs 35.3 %
2 PSMs 5.1 %
5 PSMs 12.8 %
32 PSMs 82.1 %
Modern
THN2
Kynurenine (W)
Dioxidation (W)
Oxidation (W)
unmodified

c **d**

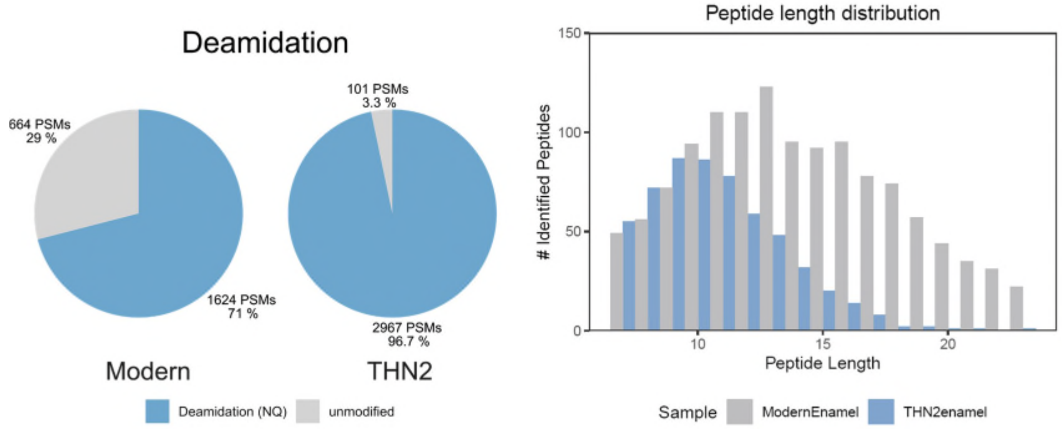
Deamidation
664 PSMs 29 %
1624 PSMs 71 %
101 PSMs 3.3 %
2967 PSMs 96.7 %
Modern
THN2
Deamidation (NQ)
unmodified
Peptide length distribution
Identified Peptides
Peptide Length
Sample
ModernEnamel
THN2enamel

e

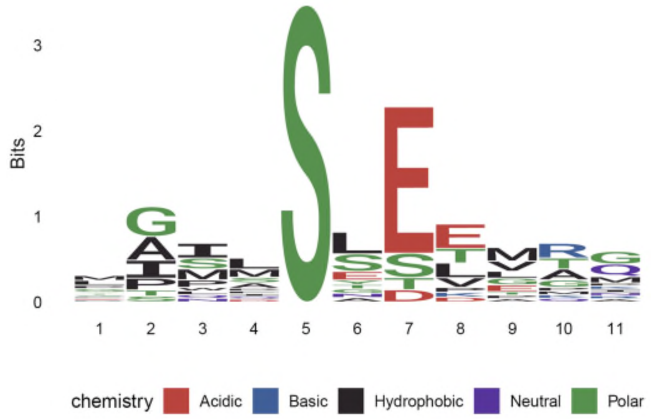
Bits
chemistry
Acidic
Basic
Hydrophobic
Neutral
Polar

Supplementary Figure 4. Quantitative analyses of diagenetic protein modifications and sequence motif analyses of serine phosphorylation sites in the TNH2-1 enamel proteome. a-c, degree of modification was approximated by spectral counts of peptides with the modified amino acid divided by all spectral counts containing the respective amino acid. **d**, peptide lengths distribution comparing TNH2-1 with a medieval enamel sample. **e**, phosphorylation is observed almost exclusively on serines (S, position 5) followed by a glutamic acid (E, position 7) in the second position towards the C-terminal end.

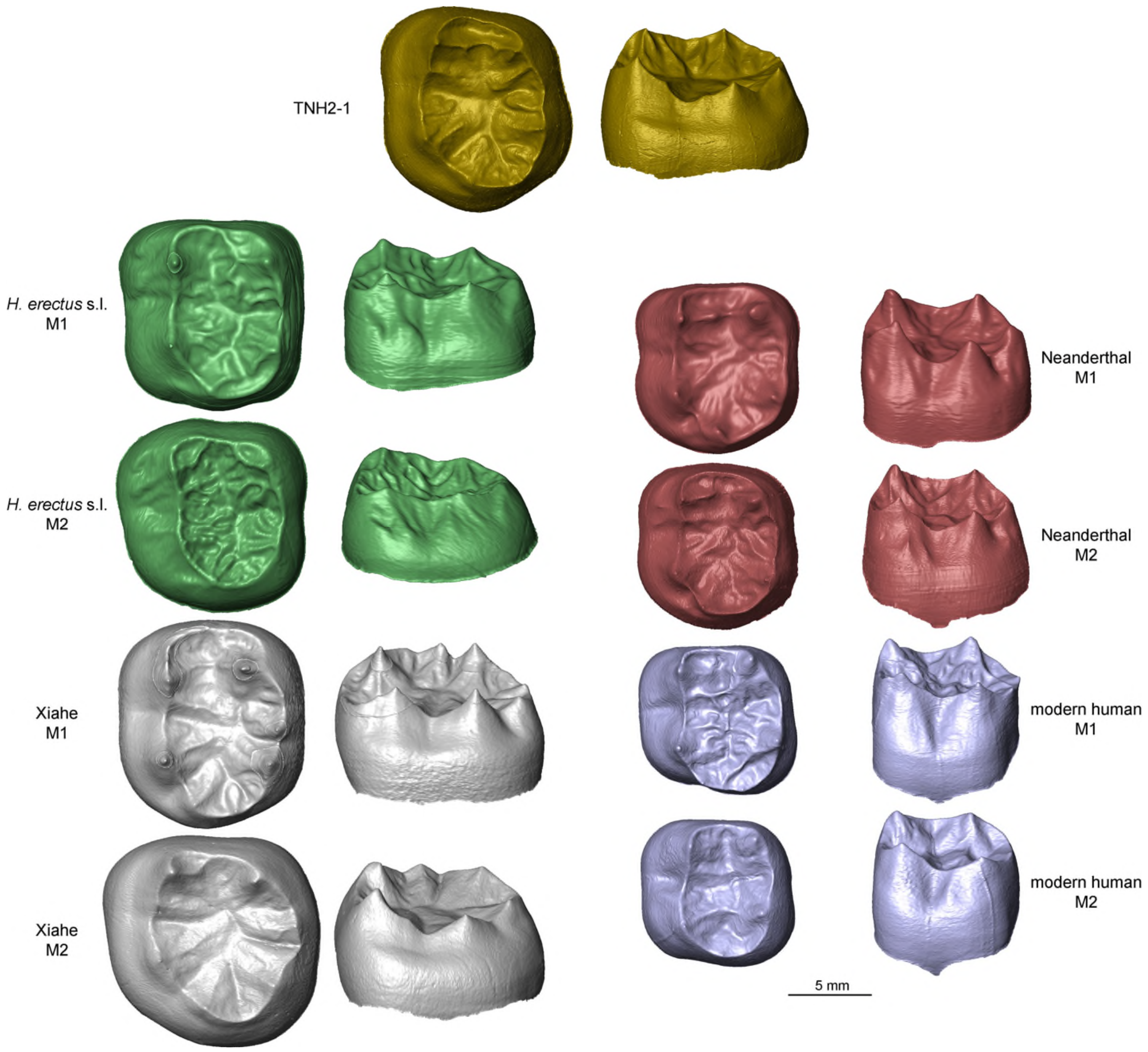

Supplementary Figure 5. Enamel-dentine junction of TNH2-1 compared with other Pleistocene and extant humans.

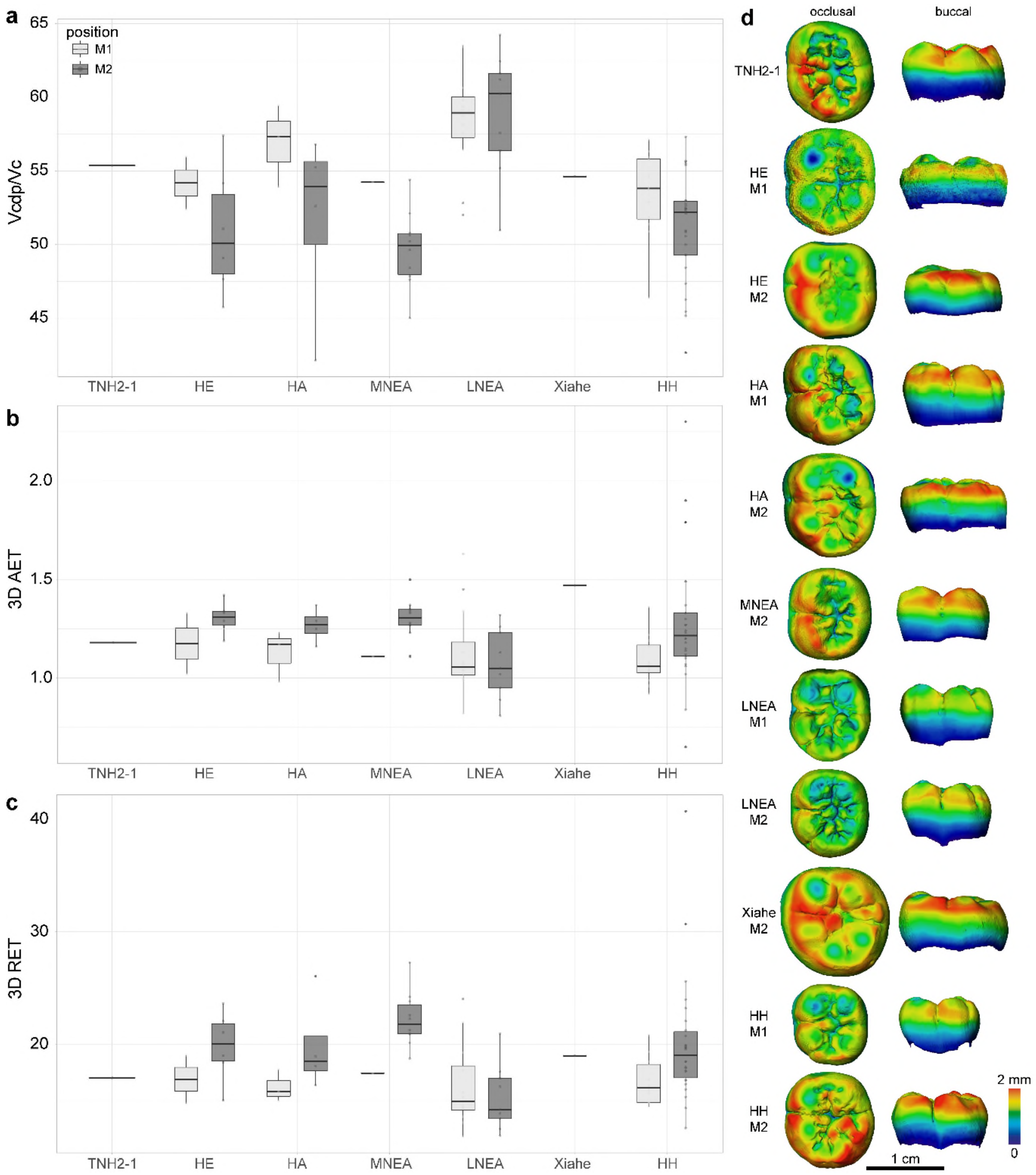

Supplementary Figure 6. Tooth tissue proportions and enamel thickness. a-d, The percent of crown dentine (**a**), three-dimensional average enamel thickness (**b**), three-dimensional relative enamel thickness (**c**) and enamel thickness distribution maps (**d**) of TNH2-1 compared with the M1s and M2s of *H. erectus* (HE; n = 8), *H. antecessor* (HA; n = 7), Middle Pleistocene Neanderthals (MNEA; n = 11), Late Pleistocene Neanderthals (LNEA; n = 21), the Xiahe M2 (Xiahe; n = 1) and Holocene humans (HH; n = 38). The boxplots show the median, the 25th and 75th percentiles (upper and lower hinges). Circles indicate outliers. Source data are provided as a Source Data file.

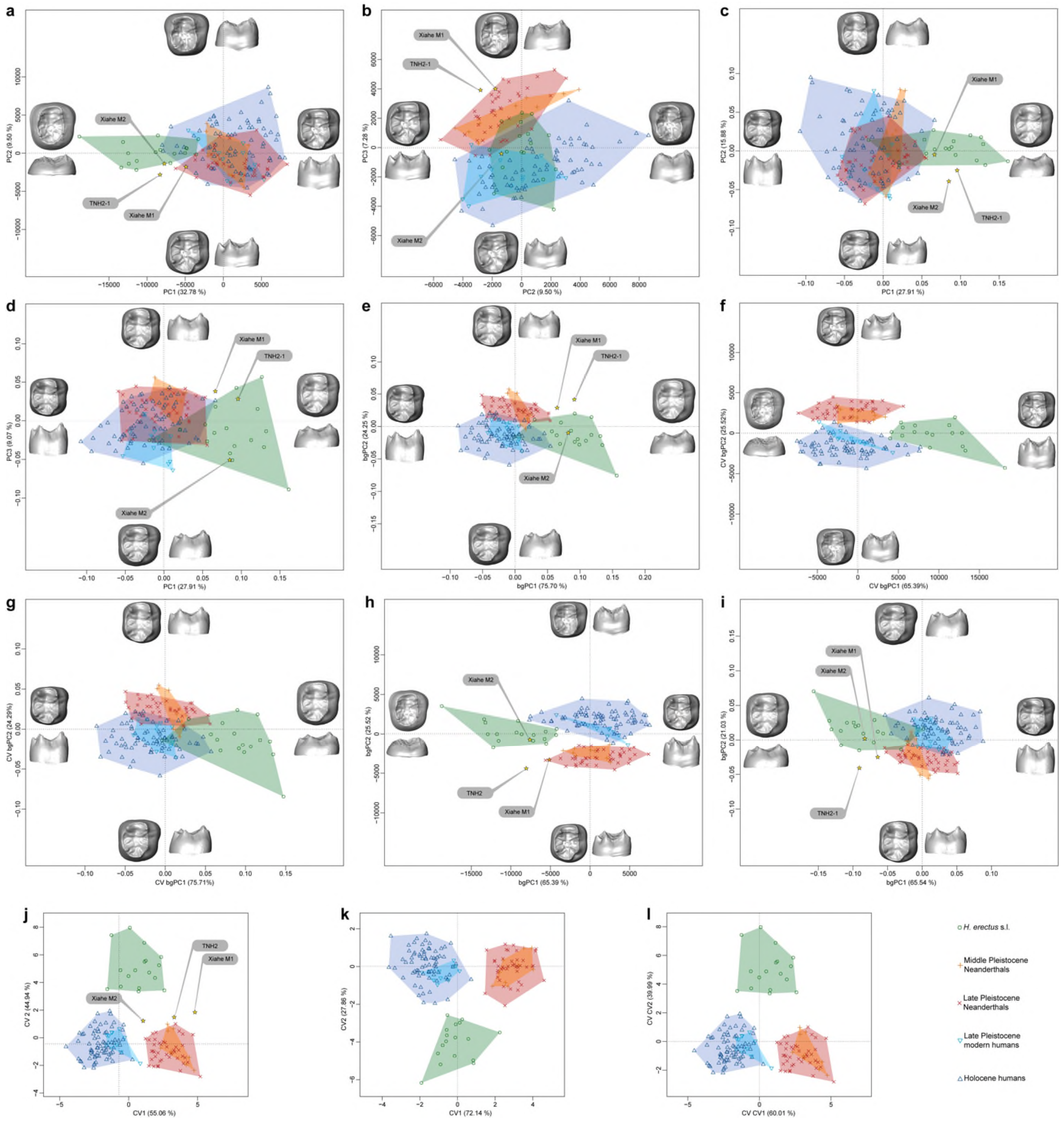

Supplementary Figure 7. Geometric morphometric analyses of the enamel-dentine junction shape. a-b, principal component analyses (**a**, PC1 vs. PC2; **b**, PC2 vs. PC3) of the EDJ deformation-based shape comparisons of TNH2-1, *H. erectus* s.l., the specimen from Xiahe, Neanderthals and modern humans. **c-d**, principal component analyses (**c**, PC1 vs. PC2; **d**, PC1 vs. PC3) of the 3D semilandmark Procrustes-registered shape coordinates based on the same sample. **e**, between group principal component analyses of the 3D semilandmark Procrustes-registered shape coordinates. **f-g**, cross-validated bgPCA of the deformation-based (**f**) and semilandmark-based analyses (**g**). **h-i**, bgPCA computed with five groups for the deformation-based (**h**) and semilandmark-based analyses (**i**). **j-l**, canonical variate analyses based on PC scores of the 3D

semilandmarks shape analyses (**j**) and cross-validated CVA of the deformation-based (**k**) and semilandmark-based analyses (**l**). Source data are provided as a Source Data file.

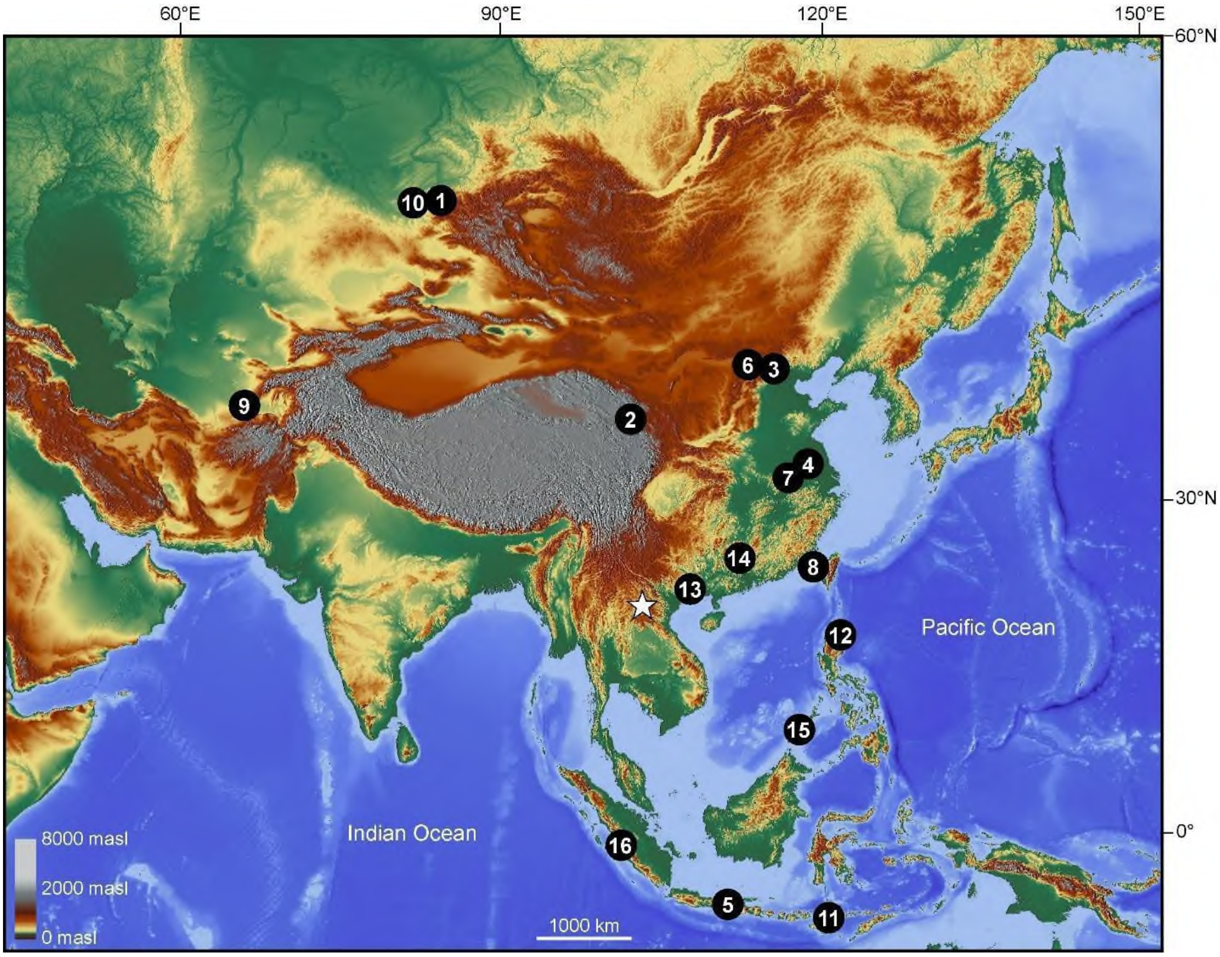

Supplementary Figure 8. Location of Tam Ngu Hao 2 cave and other localities bearing Pleistocene *Homo* remains in Asia. TNH2 and Tam Pà Ling (white star) and selected sites with identified Denisovan remains (1. Denisova Cave; 2. Baishiya Karst Cave), Middle to Late Pleistocene *H. erectus* (3. Zhoukoudian Locality 1; 4. Longtan Cave; 5. Ngandong, Ngawi and Sambungmacan sites), taxonomically-debated Middle Pleistocene *Homo* specimens (6. Xujiayao; 7. Hualong Cave; 8. Penghu), Neanderthals (9. Teshik-Tash Cave; 10. Okladnikov Cave), *H. floresiensis* (11. Liang Bua), *H. luzonensis* (12. Callao Cave) and Pleistocene *H. sapiens* (13. Zhiren Cave; 14. Daoxian; 15. Tabon Cave; 16. Lida Ajer). Map from Wikipedia (https://upload.wikimedia.org/wikipedia/commons/e/eb/Geografia_Fisica_Asia.jpg, © Contributeurs d'OpenStreetMap, under ODbL licence (Open Database License)).

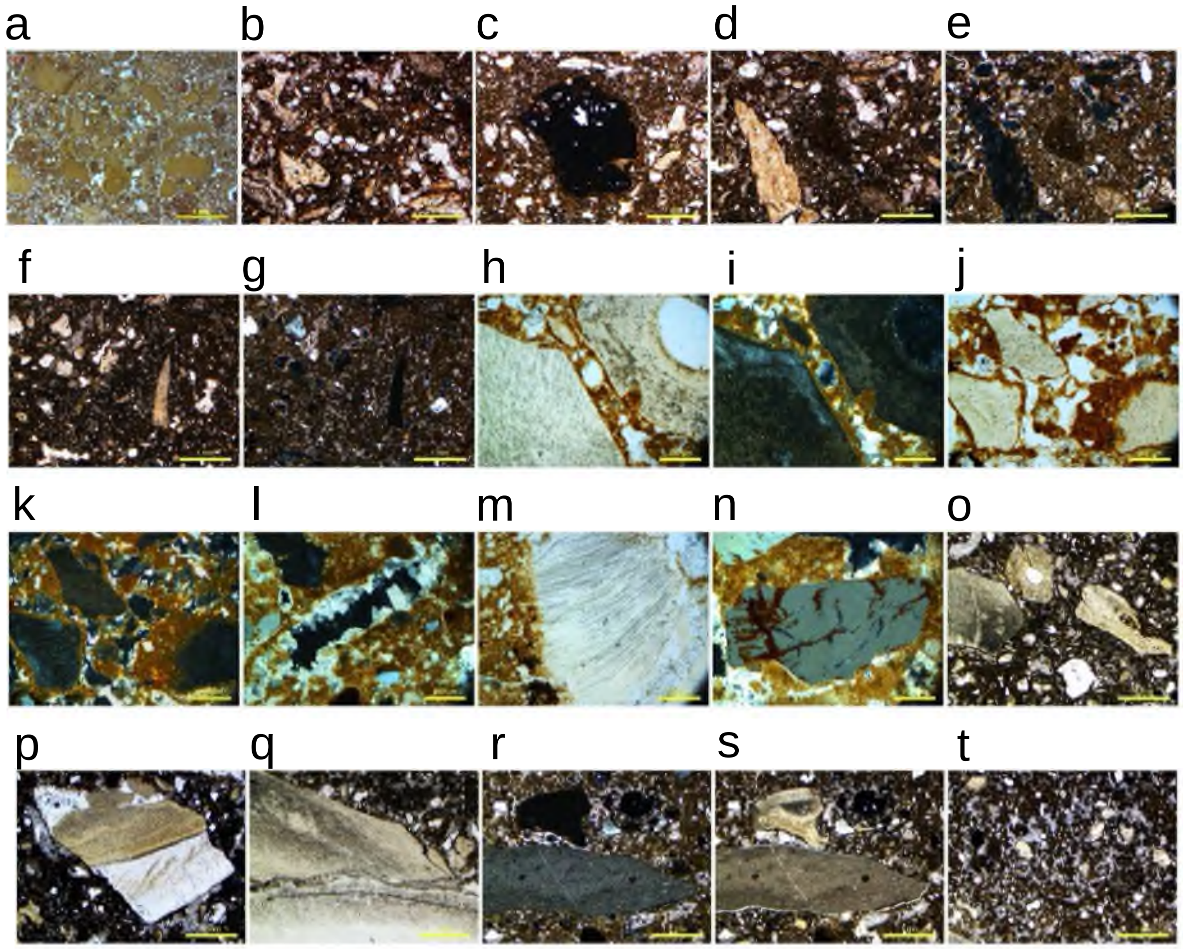

Supplementary Figure 9. Photomicrographs of key features in the Tam Ngu Hao 2 (CC) microstratigraphy. LU1 **a**, general micro-fabric of the lower unit, demonstrating that the sand-size granules are formed of aggregates of silt and clay, presumably reworked from higher up the catchment, plane polarised light (PPL), scale bar = 1mm; **b**, general micro-fabric of the upper, fossiliferous conglomerate/breccia, showing that it is formed of various organic and inorganic inclusions (primarily quartz mineral grains and small bone fragments) suspended in a silt and clay matrix. The sediment that forms this unit is generally uniform, but localised areas of increased clay content are apparent (PPL, scale bar = 1mm); **c**, Iron stained inclusion in clay-rich matrix (PPL, scale bar = 1mm); **d-e**, Large, elongate pointed bone fragment (to left) in a moderate state of preservation. The sediments here are particularly rich in clay, with some minor granostriation of the clay around a compound grain (at centre) suggesting some mass movement of the sediments (e.g., saturated debris flow). Note that the void spaces are all filled with calcite. PPL and XPL respectively, scale bar = 1mm; **f-g**, Very clay-rich sediment matrix. Bone fragment shows no signs of rounding or abrasion, although it is partially isotropic in XPL. PPL and XPL respectively, scale bar = 1mm; **h-i**, small fragments of tooth enamel (lower left) and bone (upper right, probably bird bone) in a very clay-rich matrix. PPL and XPL, scale bar = 200µm; **j-k**, small fragments of bone in moderate state of preservation, coated in undifferentiated clay coatings, possibly suggesting some reworking after initial emplacement. PPL and XPL respectively, scale bar = 200µm; **l**,

secondary calcite infilling void space. PPL, scale bar = 200 µm); **m**, tooth enamel fragment. PPL, scale bar 200 µm); **n**, weathered quartz grain, with etched surface infilled with veins of iron. These are not common in the CC microstratigraphic sequence, ruling out major reworking. XPL, scale bar = 200 µm); **o**, small, sand-sized bone and tooth fragments in a clay silt matrix with moderate amounts of fine to very fine sand, primarily quartz. PPL, scale bar = 200 µm; **p**, Large tooth fragment, with enamel dentine junction visible. Note the excellent state of preservation. XPL, scale bar = 1mm; **q** large tooth fragment with microstructure of enamel and dentine visible, with minor clay coating around top edge, possibly suggesting some minor illuviation and presence of percolating groundwater; **r-s**, horizontally-aligned, pointed fragment of microcrystalline rock, in close proximity with bone fragment. The matrix here is very clay rich and may relate to deposition or re-deposition by water; XPL & PPL respectively, scale bar = 1mm; **t**, detail of the clay-rich matrix observed in some areas of the sampled breccia, suggestive of the presence of water. PPL, scale bar = 1mm.

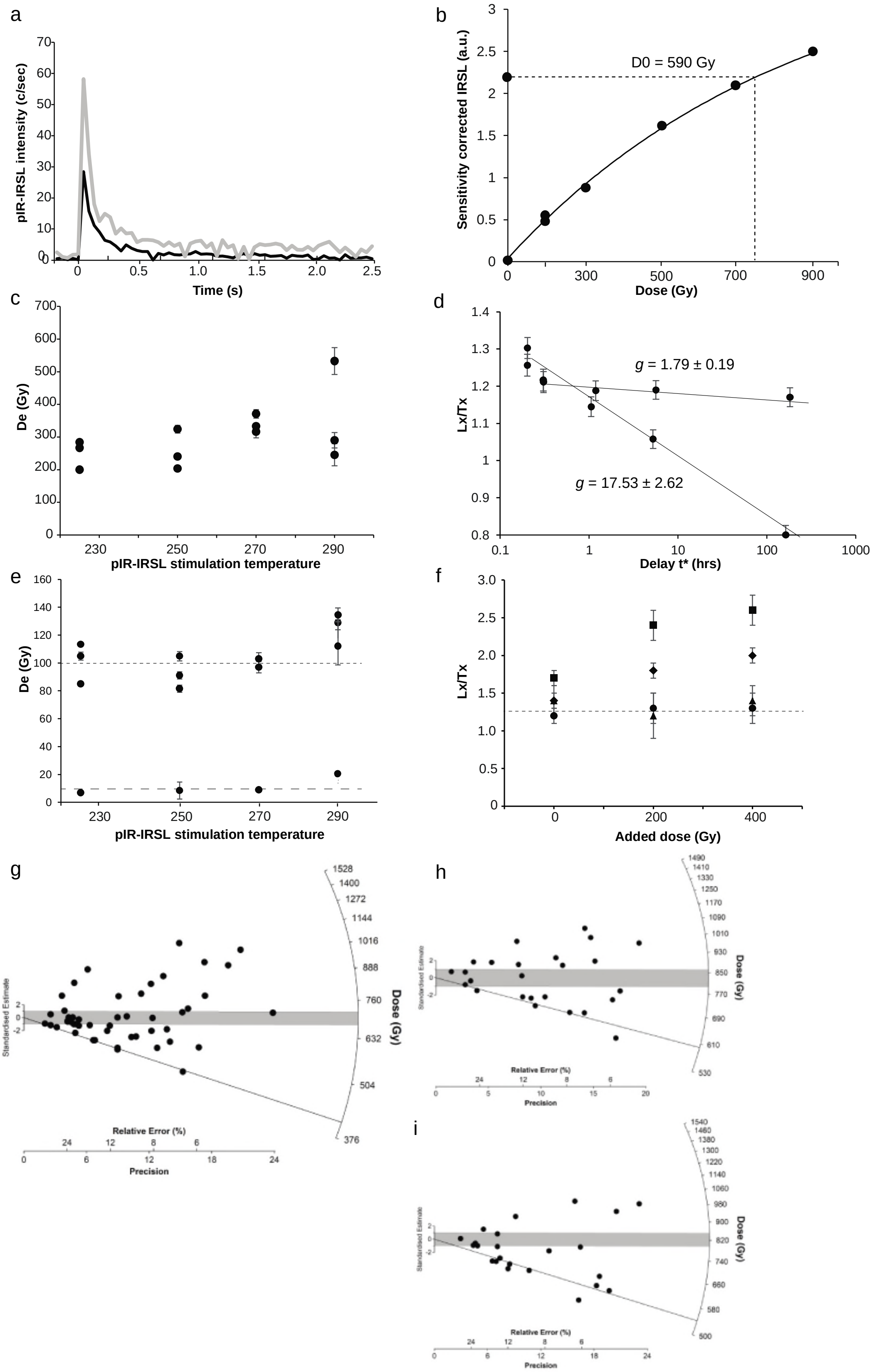
a
pIR-IRSL intensity (c/sec)
Time (s)
b
Sensitivity corrected IRSL (a.u.)
D0 = 590 Gy
Dose (Gy)
c
De (Gy)
pIR-IRSL stimulation temperature
d
Lx/Tx
g = 1.79 ± 0.19
g = 17.53 ± 2.62
Delay t* (hrs)
e
De (Gy)
pIR-IRSL stimulation temperature
f
Lx/Tx
Added dose (Gy)
g
Standardised Estimate
Dose (Gy)
Relative Error (%)
Precision
h
Standardised Estimate
Dose (Gy)
Relative Error (%)
Precision
i
Standardised Estimate
Dose (Gy)
Relative Error (%)
Precision

Supplementary Figure 10. **pIR-IRSL single-grain feldspar data for samples LCC1-3**. **a**, pIR-IRSL single-grain feldspars decays for 2.5 s of IR laser stimulation for LCC1. The black line is the natural signal and the grey line is the smallest regenerative dose at 100 Gy. **b**, dose response curve for the same feldspar grain as A demonstrating a high response to dose with a D0 value of 590 Gy and a D_e value of 755 ± 85 Gy. Data are presented as median values ± SEM. Error bars have been included in each dose point but they are too small to see. **c**, A preheat plateau test for the four different preheat and stimulation combinations pIR-$IRSL_{50,225}$, pIR-$IRSL_{50,250}$, pIR-$IRSL_{50,270}$, pIR-$IRSL_{50,290}$, using three aliquots for each combination (n=12). Error bars have been included on each D_e. **d**, fading tests over 10 days on single grains of LCC1 using the IR_{50} with a g value of 17.53 ± 2.62 indicating extreme anomalous fading. Data are presented as median values ± SEM. This is compared to the pIR-$IRSL_{270}$ signal with a much improved g value of 1.79 ± 0.19 – indicating only a small amount of fading. **e**, A dose recovery test for the four combinations described in C using three aliquots each (n=12) and a surrogate dose of 100 Gy (as indicated by the dashed line). Data are presented as median values ± SEM. **f**, Saturation experiments on feldspar grains from a weathered cobble from the breccia deposit (n=12). Data are presented as median values ± SEM. The grains should be saturated and contain an infinite age and this was tested by adding 200 and 400 Gy to the natural signal before measurement. The IR_{50} signal (squares) grew with added dose and so did the pIR-$IRSL_{50,225}$ (diamonds) to a lesser degree, but the pIR-$IRSL_{50,270}$ (circles) and pIR-$IRSL_{50,290}$ (triangles) did not grow with dose indicating saturation and minimal fading. **g**, the pIR-IRSL single grain feldspars grains plotted on a radial plot for LCC1 (n=74). The central point of 700 ± 43 Gy has been shaded in grey with a 2 sigma error margin. There is an overdispersion of 39 % indicating that this share of grains lie outside of this shading. This overdispersion value is higher than that of a normal distribution of well bleached grains indicating that some grains were partially bleached, thus we employed a MAM resulting in a fading uncorrected D_e of 411 ± 40 Gy and is indicated with a black line. The relative error of each grain ranges from 10-25%. **h**, the pIR-IRSL single grain feldspars grains for LCC2 (n=25) with an overdispersion of 26%, **i**, for LCC3 (n=23) with an overdispersion of 23%.

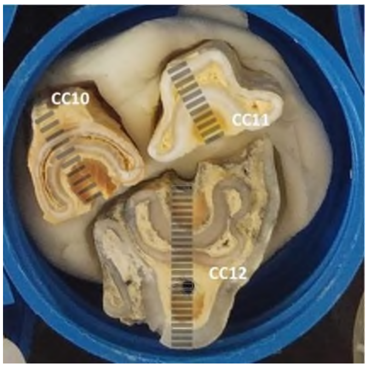

Supplementary Figure 11. Uranium series ablation tracks on the three faunal samples from Tam Ngu Hao 2.

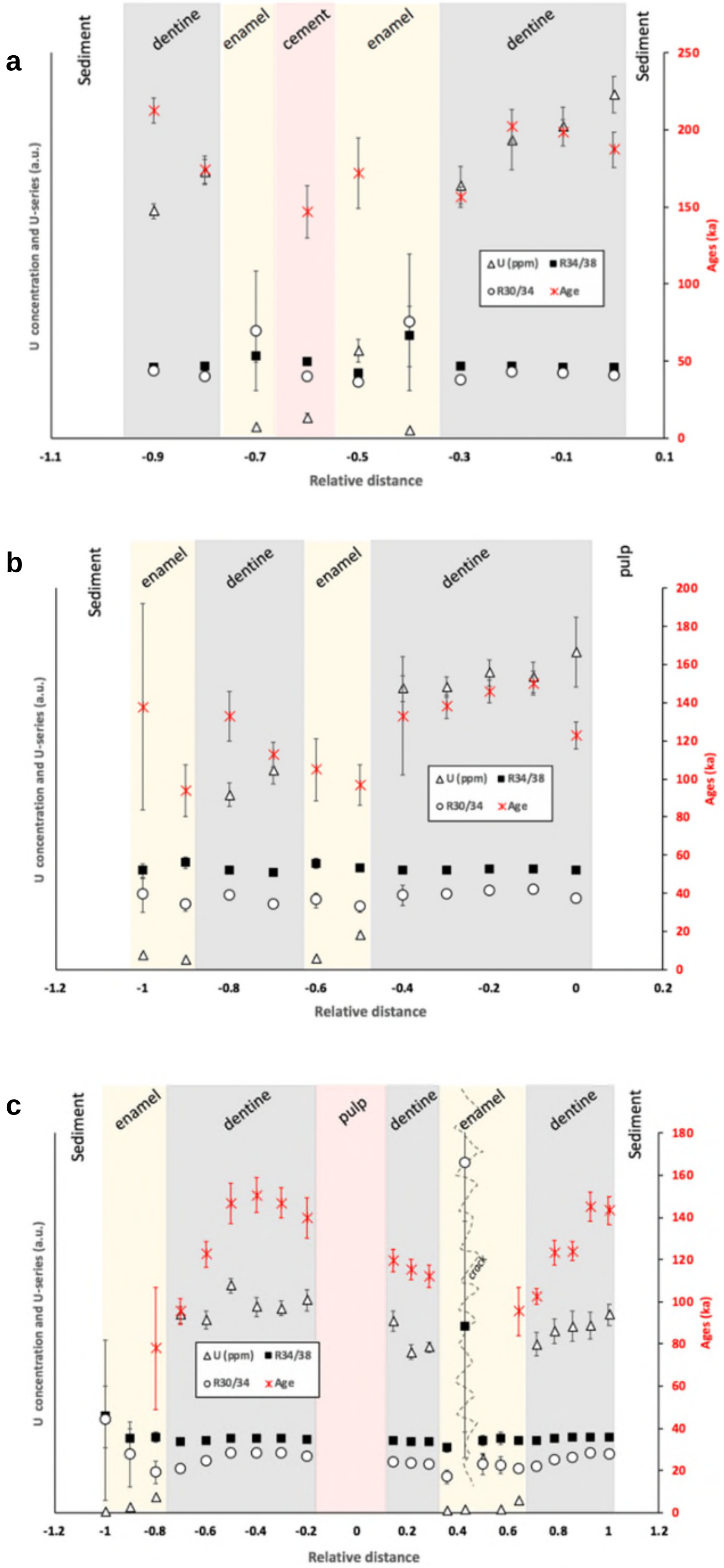
a
b
c
Sediment
dentine
enamel
cement
pulp
crack
U concentration and U-series (a.u.)
Ages (ka)
Relative distance
△ U (ppm)
■ R34/38
○ R30/34
× Age

Supplementary Figure 12. Uranium series dating results for bovid teeth (n=3) recovered from upper fossil-bearing breccia relative to the vector of diffusion. **a**, CC10. **b**, CC11. **c**, CC12. Each data point (U ppm and Isotopic ratios R34/38 and R30/34) are obtained by averaging the measurements over an entire raster, and associated error bars are standard deviation. Ages and associated uncertainties are calculated using the mean value of each raster.

OxCal (version 4.2) script for modelling ages
(https://c14.arch.ox.ac.uk/oxcal.html)

```
Options()
{
 Resolution=500;
};
Plot()
{
 Sequence("Tooth" )
 {
  //-----------------------------------------------------------------
  Phase()
  {
          //True age estimate
          Date( "Underlying_silty_unit", N(calBP(248000), 31000));
  };
  Boundary( "Underlying-to-Breccia");
  //------------------------------------------------------------------
  Phase()
  {
          //True age estimates
  Date( "US-ESR1", N(calBP(164000), 24000));
          Date( "US-ESR2", N(calBP(149000), 22000));
          Date( "US-ESR3", N(calBP(140000), 23000));
          Date( "pIR-IRSL", N(calBP(143000), 24000));
          Date( "pIR-IRSL", N(calBP(133000), 19000));
  };
  Boundary( "Breccia-to-Flowstone");
  //------------------------------------------------------------------
  Phase()
  {
          Before( )
          {
          //Minimum age
          Date( "U-series", N(calBP(104000), 27000));
          };
  };
 };
};
```

Supplementary Figure 13. OxCal (version 4.2) script for Bayesian modelling the Tam Ngu Hao 2 age estimates.

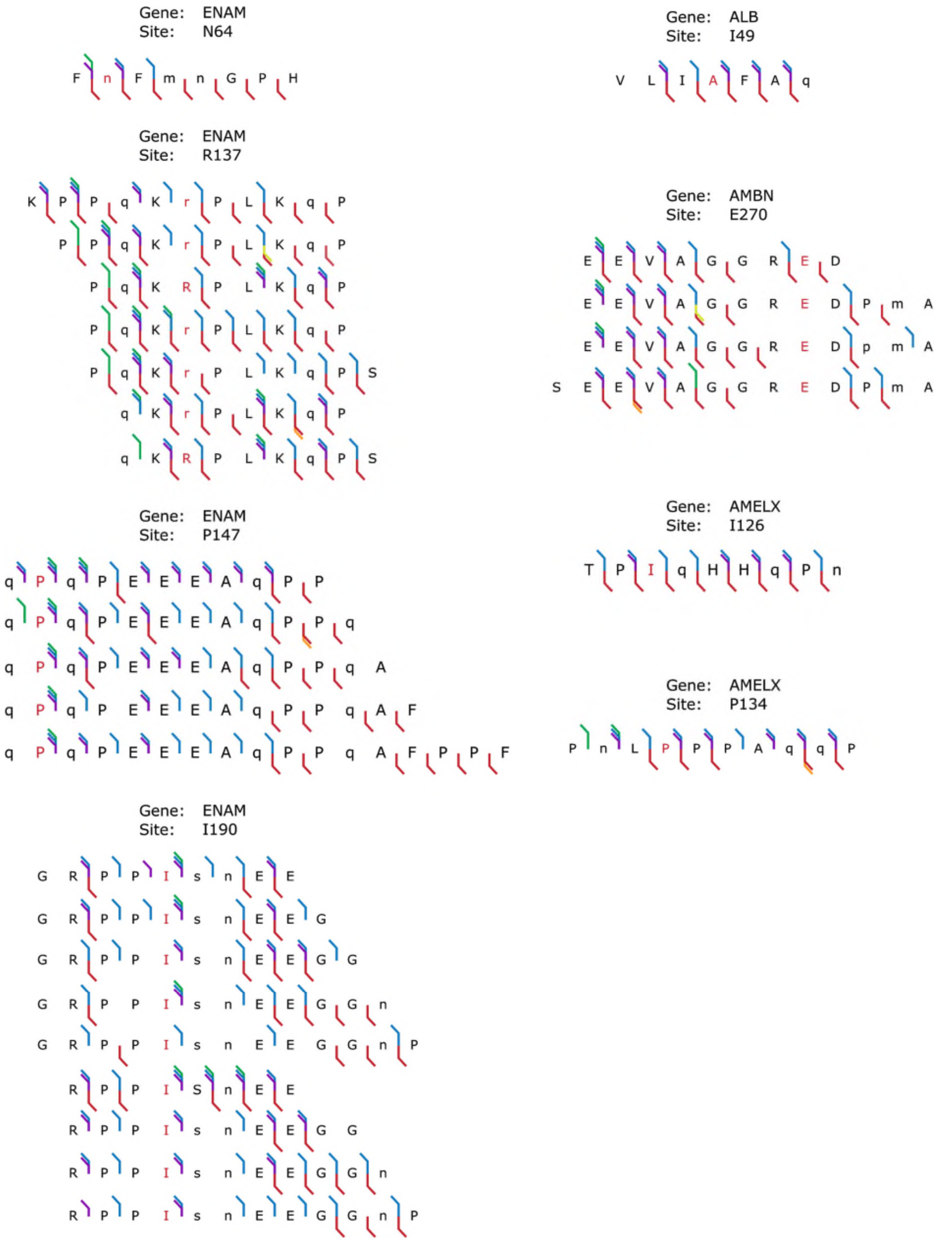

Supplementary Figure 14. Sequence alignments covering sites relevant for phylogenetic placement. Fragment types are colour coded: red (y), blue (b), green (c), purple (a), orange (z).

Letters in red represent high-confidence amino acid positions of phylogenetic interest. Lower case letters represent modified amino acids: Deamidation (qn), Oxidation (m), Hydroxyproline (p), Arginine to Ornithine (r), and phosphorylation (s).

Supplementary Table 1. Uranium series data for faunal remains. Data obtained using a Laser ablation Multicollector inductively coupled plasma mass spectrometer on the section teeth of TNH2-10/CC10, TNH2-11/CC11 and TNH2-12/CC12. Values in red do not satisfy conditions for U-series age calculations. All values are reported at 2-sigma.

Sample	Dental tissues	U (ppm)	Error (2s)	U/Th	234/238	Error (2s)	230/238	Error (2s)	Age	Error (2s)
CC10_1	dentine	5.31	0.17	2709	1.6550	0.0110	1.5590	0.0240	212.8	8.0
CC10_2	dentine	6.22	0.29	3418	1.6630	0.0130	1.4380	0.0360	174.1	8.8
CC10_3	enamel	0.25	0.02	446	1.9000	0.1300	-	-	-	-
CC10_4	enamel	0.47	0.11	297	1.7850	0.0520	1.4280	0.0900	-	-
CC10_5	cement	2.04	0.27	1	1.5140	0.0680	1.2870	0.0670	-	-
CC10_6	enamel	0.18	0.03	179	2.3800	0.7100	-	-	-	-
CC10_7	dentine	5.91	0.44	552	1.6650	0.0130	1.3650	0.0310	156.1	6.7
CC10_8	dentine	6.97	0.71	12907	1.6650	0.0110	1.5390	0.0340	202.5	10.4
CC10_9	dentine	7.28	0.44	4022	1.6580	0.0110	1.5180	0.0290	198.2	8.7
CC10_10	dentine	8.02	0.43	634	1.6450	0.0140	1.4680	0.0420	187.2	11.7
			**Average U-series age for CC10**						**188.5**	**9.1**
CC11_1	enamel	0.23	0.02	89	1.5500	0.1100	1.1800	0.2700	-	-
CC11_2	enamel	0.16	0.02	847	1.6800	0.0930	1.0200	0.1000	94.1	13.6
CC11_3	dentine	2.75	0.19	212	1.5520	0.0210	1.1590	0.0660	-	-
CC11_4	dentine	3.14	0.22	331	1.5270	0.0210	1.0330	0.0370	-	-
CC11_5	enamel	0.17	0.02	249	1.6720	0.0780	1.0900	0.1100	-	-
CC11_6	enamel	0.55	0.04	255	1.5970	0.0420	0.9830	0.0740	-	-
CC11_7	dentine	4.42	0.20	2244	1.5650	0.0470	1.1700	0.1600	133.0	31.0
CC11_8	dentine	4.45	0.16	1802	1.5630	0.0140	1.1920	0.0310	137.9	6.3
CC11_9	dentine	4.67	0.20	1213	1.5810	0.0120	1.2440	0.0280	145.8	6.0
CC11_10	dentine	4.60	0.24	757	1.5790	0.0130	1.2620	0.0290	150.2	6.4
CC11_11	dentine	4.99	0.55	70	1.5530	0.0200	1.1080	0.0400	-	-
			**Average U-series age for CC11**						**132.2**	**12.6**
CC12_1	enamel	0.03	0.00	216	2.2700	0.7400	2.2000	1.9000	-	-
CC12_2	enamel	0.13	0.01	1299	1.7500	0.2400	1.3700	0.7700	141.7	138.0
CC12_3	enamel	0.36	0.02	4500	1.7800	0.1200	0.9500	0.2700	77.9	29.1
CC12_4	dentine	4.69	0.20	2333	1.6800	0.0180	1.0290	0.0470	95.4	6.2
CC12_5	dentine	4.57	0.22	2308	1.7010	0.0140	1.2220	0.0370	122.5	6.0
CC12_6	dentine	5.38	0.18	5327	1.7530	0.0270	1.3990	0.0490	146.8	9.4
CC12_7	dentine	4.88	0.22	9208	1.7430	0.0150	1.4090	0.0420	150.5	8.1
CC12_8	dentine	4.84	0.17	3811	1.7500	0.0120	1.3970	0.0380	146.9	7.1
CC12_9	dentine	5.04	0.25	851	1.7130	0.0190	1.3280	0.0520	139.7	9.5
CC12_10	pulp	0.16	0.08	0	1.2400	0.1500	0.9300	0.3200	-	-

CC12_11	pulp	0.00	0.00	1	-7.4000	7.1000	-11.00	24.0000	-	-
CC12_12	pulp	0.01	0.00	6	0.4000	1.7000	12.00	12.0000	-	-
CC12_13	dentine	4.55	0.24	1188	1.6970	0.0160	1.2010	0.0340	119.5	5.3
CC12_14	dentine	3.81	0.18	1124	1.6820	0.0150	1.1650	0.0310	115.5	4.8
CC12_15	dentine	3.93	0.12	1314	1.6690	0.0150	1.1340	0.0340	112.1	5.2
CC12_16	enamel	0.06	0.01	250	1.5300	0.1100	0.8500	0.1500	-	-
CC12_17	enamel	0.07	0.01	61	4.4000	2.5000	8.3000	7.0000	-	-
CC12_18	intrusion	1.27	0.09	72	1.7100	0.1100	1.1500	0.2500	-	-
CC12_19	enamel	0.07	0.00	287	1.7600	0.1500	1.1200	0.2000	-	-
CC12_20	enamel	0.28	0.02	547	1.6890	0.0670	1.0350	0.0830	95.4	11.3
CC12_21	dentine	3.99	0.28	599	1.7020	0.0130	1.0930	0.0270	102.3	3.7
CC12_22	dentine	4.30	0.29	765	1.7380	0.0170	1.2550	0.0360	123.2	5.7
CC12_23	dentine	4.42	0.36	1091	1.7720	0.0130	1.2870	0.0300	124.0	4.6
CC12_24	dentine	4.43	0.32	1030	1.7700	0.0140	1.4050	0.0380	145.1	6.9
CC12_25	dentine	4.69	0.25	494	1.7650	0.0150	1.3910	0.0380	143.2	6.8
				**Average U-series age for CC12**					**123.6**	**15.7**

Supplementary Table 2. US-ESR dating data.

Sample	CC10	CC11	CC12
	Enamel		
Dose (Gy)[a]	243.1±8.9	186.7±7.7	166.2±10.2
U (ppm)[b]	0.36±0.06	0.19±0.02	0.16±0.02
$^{234}U/^{238}U$ [b]	1.8425±0.0910	1.6340±0.0646	1.7170±0.2253
$^{230}Th/^{234}U$ [b]	0.8874±0.0197*	0.6711±0.0216	0.7035±0.0189
Thickness (m)	917±226	1054±173	1442±287
Water (%)	3±1	3±1	3±1
	Dentine		
U (ppm) [b]	5.96±0.39	4.14±0.25	4.54±0.23
$^{234}U/^{238}U$ [b]	1.6378±0.0203	1.5682±0.0212	1.7239±0.0159
$^{230}Th/^{234}U$ [b]	0.8874±0.0197	0.7480±0.0201	0.7334±0.0157
Water (%)	5±3	5±3	5±3
	Sediment#		
U (ppm)	3.06±0.14	3.06±0.14	3.06±0.14
Th (ppm)	5.16±0.46	5.16±0.46	5.16±0.46
K (%)	1.83±0.59	1.83±0.59	1.83±0.59

Water (%)	5±2	5±2	5±2
	**E**xternal dose rate sediment		
Beta dose (µGy a^{-1})	253±79	215±56	158±47
Gamma Dose (µGy a^{-1})[d]	896±155	896±155	896±155
Cosmic (µGy a^{-1})	55±2	55±2	55±2
	**C**ombined **US-ESR A**ges		
Internal dose rate (µGy a^{-1})[c]	179±123	47±47	45±45
Internal Beta dose (µGy a^{-1})[c]	99±68	40±40	33±33
P enamel [c]	-1.0504±0.0790	-0.7575±0.1319	-0.8739±0.1295
P dentine [c]	-1.660±0.0772	-0.9217±0.1067	-0.9293±0.1201
Total dose rate (µGy a^{-1})[c]	1482±224	1253±192	1187±208
**AGE (kyr) [C]**	**164±24**	**149±22**	**140±23**

[a] Dose equivalent D_e obtained using MCDoseE 2.0, with SSE (from Joannes-Boyau *et al.*, in press).
[b] Uranium concentration values were obtained by LA-MC-ICPMS and LA-ICPMS on both teeth and both dental tissues.
[c] The age was calculated using [5], with the dose rate conversion factors of [6], alpha efficiency and beta attenuation as well as enamel and dentine densities are from [7].
[d] The external gamma dose rate was calculated using the U, Th, and K concentration obtained by ICPSM on the surrounding sediments and assuming a 4π geometry.
* the $^{230}Th/^{234}U$ values in the enamel for this sample could not be modelled with the values measured >1, and therefore the ratio in the enamel was assumed to be identical to the dentine ratio.
# Breccia surrounding the teeth was collected and measured by ICPMS solution to obtain U, Th and K concentrations.

Supplementary Table 3. pIR-IRSL dating of sediments at Tam Ngu Hao 2: dose rate data, equivalent doses, and age estimates.

A: Alpha and beta counting

Sample code[a]	Depth from roof (m)	Gamma dose rate (Gy ka^{-1})[b]	Beta dose rate (Gy ka^{-1})[b]	Cosmic-ray dose rate (Gy ka^{-1})[c]	Internal dose rate (Gy ka^{-1})[d]	Water content (%)[e]	Total dose rate (Gy ka^{-1})	Run/ Accepted grains[f]	Equivalent dose (Gy)[g,h]	Fading Corrected De[i] (Gy)	IRSL age (ka)[h]
**LCC1**	0.8	1.034 ± 0.030	1.631 ± 0.062	0.055 ± 0.002	0.84 ± 0.08	5 / 5 ± 2	3.560 ± 0.137	2400/47	411 ± 40	485 ± 72	136 ± 21
**LCC2**	1.3	1.677 ± 0.057	2.425 ± 0.079	0.055 ± 0.006	0.84 ± 0.08	7 / 5 ± 2	4.997 ± 0.184	2500/31	600 ± 27	708 ± 56	142 ± 13
**LCC3**	2.2	0.717 ± 0.033	1.057 ± 0.035	0.054 ± 0.005	0.84 ± 0.08	3 / 3 ± 0.2	2.668 ± 0.106	3400/23	543 ± 20	662 ± 77	**248 ± 31**

[a] Samples processed using the 180-212 µm size fraction. Fig 1b contains the sampling location
[b] Beta dose rates were estimated using a Geiger Muller beta counting of dried and powdered sediment samples, gamma dose rates were estimated using thick source alpha counting measurements of dried and powdered sediment samples in the laboratory. The difference between these measurements was used to estimate potassium values.
[c] Time-averaged cosmic-ray dose rates (for dry samples), each assigned an uncertainty of ± 10%.
[d] An internal dose rate of 0.84 ± 0.08 Gy/ka was estimated for all feldspar grains. Mean ± total (1σ) uncertainty, calculated as the quadratic sum of the random and systematic uncertainties
[e] Field / time-averaged water contents, expressed as (mass of water/mass of dry sample) x 100. The latter values were used to correct the external gamma and beta dose rates.
[f] Total number of feldspar grains processed verses number of accepted grains - with an average acceptance rate of 1.22%
[g] Equivalent doses include a ± 2% systematic uncertainty associated with laboratory beta-source calibrations.
[h] Uncertainties at 68% confidence interval.
[I] Fading corrections according to Lamothe et al. (2003)

B High resolution gamma spectrometry

Sample code[a]	^{238}U (Bq/kg)	^{226}Ra (Bq/kg)	^{210}Pb (Bq/kg)	^{228}Ra (Bq/kg)	^{228}Th (Bq/kg)	^{40}K (Bq/kg)	Water content (%)	Total dose rate[b] (Gy ka^{-1})	Equivalent dose (Gy)	Fading corrected De[c]	IRSL age[d] (ka)
**LCC1**	31 ± 11	29.6 ± 2.9	24.7 ± 9.8	46.5 ± 4.8	47.7 ± 5.1	467 ± 50	5 / 5 ± 2	3.393 ± 0.256	411 ± 40	485 ± 72	**143 ± 24**
**LCC2**	58 ± 10	48.7 ± 4.5	37.9 ± 8.3	78.9 ± 8.0	84.9 ± 8.2	889 ± 95	7 / 5 ± 2	5.307 ± 0.388	600 ± 27	708 ± 56	**133 ± 19**

[a] High-resolution gamma spectrometry to estimate the U, Th and K concentrations in the dried and powdered sample, provide a comparison of dose rate, and to test the degree of disequilibrium occurring at the site. HRGS was measured for only samples LCC1 and LCC2. All other parameters such as cosmic dose rate, internal dose rate and number of grains remains the same as Table A.

[b] The total dose rate for LCC1 is very similar but LCC2 is slightly higher than Table A resulting in a lower age estimate.

[c] Fading corrections according to Lamothe et al. (2003) [8]

[d] The IRSL ages have been calculated using these alternate dose rate estimations.

Supplementary Table 4. U-series dating of the overlying flowstone in Tam Ngu Hao 2.

Sample Name	U (ppm)	^{232}Th (ppb)	(^{230}Th/ ^{232}Th)	(^{230}Th/^{238}U)	(^{234}U/ ^{238}U)	Uncorr. ^{230}Th Age (kyr)[a]	Corr. ^{230}Th Age I (kyr)[a]	corr. Initial (^{234}U/ ^{238}U)	Corr. ^{230}Th Age-II (kyr)[b]
**LCC1**	0.1685 ± 0.0001	569.9 ± 0.91	0.905 ± 0.003	1.009 ± 0.003	1.281 ± 0.002	153 ± 1	30 ± 149	1.838 ± 0.44	97 ± 38
**LCC1-T**	0.4114 ± 0.0005	1416 ± 8	1.024 ± 0.008	1.162 ± 0.007	1.305 ± 0.002	202 ± 3	89 ± 995	2.078 ± 0.47	150 ± 100
**LCC1-M**	0.2072 ± 0.0002	710 ± 2	0.917 ± 0.005	1.035 ± 0.004	1.291 ± 0.001	159 ± 1	35 ± 242	1.897 ± 0.47	104 ± 46
**LCC1-B**	0.9144 ± 0.0017	3936 ± 35	0.841 ± 0.011	1.193 ± 0.012	1.320 ± 0.002	209 ± 6	negative value	2.638 ± 1.33	141 ± 212

[a] Corrected Age-I (ka) was calculated by applying non-radiogenic ^{230}Th correction assuming non-radiogenic ^{230}Th/^{232}Th activity ratio = 0.825 with an arbitrarily assigned uncertainty of 50% (bulk-Earth value), with ^{238}U, ^{234}U, ^{232}Th and ^{230}Th in secular equilibrium. This scheme is not appropriate as it results in ^{230}Th over-correction evidenced by sample LCC1-B returning a negative age value.

[b] Corrected Age-II (ka) was calculated by applying non-radiogenic ^{230}Th correction assuming non-radiogenic ^{230}Th/^{232}Th activity ratio = 0.5 with an arbitrarily assigned uncertainty of 50% (bulk-Earth value), with ^{238}U, ^{234}U, ^{232}Th and ^{230}Th in secular equilibrium. The value of 0.5 was estimated through (^{234}U/^{232}Th) vs. (^{230}Th/^{232}Th) best-fit line intercepting on (^{230}Th/^{232}Th)-axis. In other words, it represents the common initial ^{230}Th/^{232}Th activity ratio of these sub-samples. Thus, this correction scheme is more appropriate. The four repeats give indistinguishable Corrected Age-II values with a weighted mean of 104 ± 27 ka.

Supplementary Table 5. Number of Identified specimens (NISP) and corresponding minimum number of Individuals (MNI) inventoried in the TNH2 faunal assemblage. MNI has calculated using the most frequent tooth type, within permanent or deciduous sets of teeth. *MNIs are not provided for “Rhinocerotina indet.” and “*Rhinoceros* sp.” as there is no means for discarding any overlap with those of *Rhinoceros sondaicus* and/or *Dicerorhinus* sp. Stegodon sp. is only represented by fragments of enamel.

	**Permanent teeth**		**Deciduous teeth**		**Total**	
**Taxa**	**NISP**	**MNI**	**NISP**	**MNI**	**NISP**	**MNI**
Sus scrofa	37	3 (lp3)	1	1	38	4
Bos sp. (cf. *frontalis*)	29	3 (rp3)	6	2 (rd4)	35	5
Naemorhedus sp.	10	1	-	-	10	1
Small-sized cervid	21	2 (p2)	3	1	24	4
Medium-sized cervid	3	1	3	1	6	2
Large-sized cervid	12	2 (p3)	4	1	16	3
Rhinocerotina indet.	9	-*	3	-*	12	-*
Rhinoceros sp.	2	-*	1	-*	3	-*
Rhinoceros sondaicus	1	1	1	1	2	2
Dicerorhinus sp.	1	1	0	0	1	1
Tapirus sp.	-	-	1	1	1	1
Small-sized carnivora	2	1	-	-	2	1
Small-sized felid	1	1	-	-	1	1
Paradoxurus sp.	1	1	-	-	1	1
Ursus thibetanus	3	1	-	-	3	-
Ailuropoda sp.	2	1	-	-	2	1
Macaca cf. *nemestrina*	10	1	-	-	10	1
Pongo sp.	1	1	-	-	1	1
Hystrix sp.	18	2 (rp4)	-	-	18	2
	163	23	23	8	186	31

Supplementary Table 6. Endogenous proteome composition of TNH2-1. For each of the four injections, the number of peptides and the number of razor+unique peptides (in brackets) are indicated. The human reference accession refers to the UniProt accession, available at uniprot.org. Peptides counts include all peptides identified by MaxQuant. TNH2-1 amino acid counts and coverage after spectral quality filtering and GenBank/UniProt protein blast.

Protein	Human ref. accession	Peptides HCl_01	Peptides HCl_02	Peptides TFA_01	Peptides TFA_02	TNH2 amino acid count	TNH2 coverage (%)
AHSG	P02765	2 (2)	1 (1)	0 (0)	0 (0)	47	12.77
ALB	P02768	14 (14)	8 (8)	8 (8)	4 (4)	71	11.66
AMBN	Q9NP70	53 (53)	72 (72)	77 (77)	72 (72)	121	27.1
AMELX, isoform 3	Q99217-3	112 (18)	136 (14)	140 (13)	132 (11)	161	78.54
AMELX, isoform 1	Q99217	97 (97)	125 (125)	135 (135)	127 (127)	147	76.96
AMTN	Q6UX39	2 (2)	2 (2)	0 (0)	0 (0)	18	8.61
COL1A1	P02452	1 (1)	1 (1)	4 (4)	5 (5)	21	1.43
ENAM	Q9NRM1	123 (123)	106 (106)	98 (98)	105 (105)	122	10.68
MMP20	O60882	1 (1)	0 (0)	1 (1)	0 (0)	18	3.73

Supplementary Table 7. Analyses of protein modifications. Spectral counts of modified peptides and overall PSMs of peptides containing the respective amino acids in four LC-MS/MS runs of TNH2-1 and two runs of the recent reference sample (Ø1952 [9]). AA is amino acid.

		**Modified PSMs**		**All PSMs of the AA**		**Relative modified PSMs**	
**PTM**	**AA**	**Recent**	**THN2**	**Recent**	**THN2**	**Recent**	**THN2**
Glu->pyro-Glu	E	107	534	184	761	58.2%	70.2%
Gln->pyro-Glu	Q	88	215	99	453	88.9%	47.5%
Hydroxyproline	P	652	456	3061	4020	21.3%	11.3%
Phospho (STY)	STY	248	90	2602	1274	9.5%	7.1%
Ornithine (R)	R	3	249	1097	859	0.3%	29.0%
Kynurenine (W)	W	26	5	1035	39	2.5%	12.8%
Dioxidation (W)	W	49	32	1035	39	4.7%	82.1%
Oxidation (W)	W	365	2	1035	39	35.3%	5.1%
Deamidation (NQ)	NQ	1624	2967	2288	3068	71.0%	96.7%
Oxidation (M)	M	551	2058	623	2123	88.4%	96.9%

Supplementary Table 8. Informative amino acid positions covered in the TNH2-1 proteome.
For each hominid genus, the amino acid observed in the reference sequences is indicated, as is the amino acid observed in the TNH2-1 enamel proteome.

**Protein**	**Human ref. accession**	**Amino acid position***	***Pongo* sp.**	***Gorilla* sp.**	***Pan* sp.**	***Homo* sp.**	**Amino acid in TNH2**	**Consequence**
ALB	P02768	49	I	V	V	I	I	Not *Gorilla*, not *Pan*
AHSG	P02765	21	H	H	R	H	H	Not *Pan*
AHSG	P02765	23	P	P	L	P	P	Not *Pan*
AMBN	Q9NP70	270	G	E	E	E	E	Not *Pongo*
ENAM	Q9NRM1	64	N	N	T	N	N	Not *Pan*
ENAM	Q9NRM1	137	Q	R	R	R	R	Not *Pongo*
ENAM	Q9NRM1	147	A	P	P	P	P	Not *Pongo*
ENAM	Q9NRM1	190	I	I	M	I	I	Not *Pan*
AMELX	Q99217-3	126	T	I	I	I	I	Not *Pongo*
AMELX	Q99217-3	134	L	P	P	P	P	Not *Pongo*

Supplementary Table 9. Comparative Pleistocene and Holocene human samples used for morphological and metrical analyses.

Specimen	Locality/ collection (country)	Taxon	Chronology	Crown diameters		Tissue proportions		EDJ shape		Refs
				**M1**	**M2**	**M1**	**M2**	**M1**	**M2**	
KNM-ER 806	Koobi Fora (Kenya)	*H. erectus* s.l.	Early Pleistocene	1	1			1		[10], original data
KNM-ER 820	Koobi Fora (Kenya)	*H. erectus* s.l.	Early Pleistocene	2						[10]
KNM-ER 992	Koobi Fora (Kenya)	*H. erectus* s.l.	Early Pleistocene	2	2			2	2	[10], original data
KNM-ER 1808	Koobi Fora (Kenya)	*H. erectus* s.l.	Early Pleistocene		1					[10]
KNM-WT 15000	Lake Turkana (Kenya)	*H. erectus* s.l.	Early Pleistocene	2	2					[10]
OH 22	Olduvai Gorge (Tanzania)	*H. erectus* s.l.	Early Pleistocene	1	1					[10]

Tighenif 1	Tighenif (Algeria)	*H. erectus* s.l.	Early Pleistocene	1	1					[10]
Tighenif 2	Tighenif (Algeria)	*H. erectus* s.l.	Early Pleistocene	1	1		1		1	[10,11]
Tighenif 3	Tighenif (Algeria)	*H. erectus* s.l.	Early Pleistocene	1	1					[10]
MA 93	Mulhuli-Amo (Eritrea)	*H. erectus* s.l.	Early Pleistocene			1		1		[12]
Sangiran 1b	Sangiran (Indonesia)	*H. erectus* s.l.	Early Pleistocene	1	1			1	1	[10,13]
Sangiran 7-64	Sangiran (Indonesia)	*H. erectus* s.l.	Early Pleistocene		1				1	[14], *original data*
Sangiran 7-65	Sangiran (Indonesia)	*H. erectus* s.l.	Early Pleistocene		1					[14]
Sangiran 7-20	Sangiran (Indonesia)	*H. erectus* s.l.	Early Pleistocene	1				1		[14], *original data*
Sangiran 22	Sangiran (Indonesia)	*H. erectus* s.l.	Early Pleistocene	2	2					[15]

Sangiran 24	Sangiran (Indonesia)	*H. erectus* s.l.	Early Pleistocene		1					[15]
Sangiran 37	Sangiran (Indonesia)	*H. erectus* s.l.	Early Pleistocene		1					[15]
Ardjuna 8	Sangiran (Indonesia)	*H. erectus* s.l.	Early Pleistocene		1					[15]
Sb 8103	Sangiran (Indonesia)	*H. erectus* s.l.	Early Pleistocene	1	1					[15]
Ng 8503	Sangiran (Indonesia)	*H. erectus* s.l.	Early Pleistocene	1	1					[15]
SMF-PA-F-8865	Sangiran (Indonesia)	*H. erectus* s.l.	Early Pleistocene				1		1	[13]
MI92.1	Sangiran (Indonesia)	*H. erectus* s.l.	Early-Middle Pleistocene	1						[16]
NG92.2	Sangiran (Indonesia)	*H. erectus* s.l.	Early-Middle Pleistocene	1						[16]

NG0802.2	Sangiran (Indonesia)	*H. erectus* s.l.	Early-Middle Pleistocene	1	1	[16,17]
NG0802.3	Sangiran (Indonesia)	*H. erectus* s.l.	Early-Middle Pleistocene	1	1	[16,17]
NG92.1	Sangiran (Indonesia)	*H. erectus* s.l.	Early-Middle Pleistocene	1		[16]
NG92.3	Sangiran (Indonesia)	*H. erectus* s.l.	Early-Middle Pleistocene	1	1	[16,17]
NG92.4	Sangiran (Indonesia)	*H. erectus* s.l.	Early-Middle Pleistocene	1		[16]
NG92.D6 ZE 57s/d 76	Sangiran (Indonesia)	*H. erectus* s.l.	Early-Middle Pleistocene	1	1	[16,17]
PCG09_KII_Z1.37	Sangiran (Indonesia)	*H. erectus* s.l.	Early-Middle Pleistocene	1		[16]

CA 808	Apothecary Collection (China)	*H. erectus* s.l.	Middle Pleistocene			1	[18,19]
ZKD A1-1	Zhoukoudian (China)	*H. erectus* s.l.	Middle Pleistocene	1			[10]
ZKD A2-2	Zhoukoudian (China)	*H. erectus* s.l.	Middle Pleistocene	1	1		[10]
ZKD A3-56	Zhoukoudian (China)	*H. erectus* s.l.	Middle Pleistocene	1			[10]
ZKD B1-3	Zhoukoudian (China)	*H. erectus* s.l.	Middle Pleistocene	1			[10]
ZKD B1-63	Zhoukoudian (China)	*H. erectus* s.l.	Middle Pleistocene		1		[10]
ZKD B3-9	Zhoukoudian (China)	*H. erectus* s.l.	Middle Pleistocene	1			[10]
ZKD B4-75	Zhoukoudian (China)	*H. erectus* s.l.	Middle Pleistocene		1		[10]
ZKD C3-45	Zhoukoudian (China)	*H. erectus* s.l.	Middle Pleistocene	1			[10]

ZKD D1-43	Zhoukoudian (China)	*H. erectus* s.l.	Middle Pleistocene		1	[10]
ZKD F1-5	Zhoukoudian (China)	*H. erectus* s.l.	Middle Pleistocene	1		[10]
ZKD G1-6	Zhoukoudian (China)	*H. erectus* s.l.	Middle Pleistocene	1	1	[10]
ZKD I1-PA87	Zhoukoudian (China)	*H. erectus* s.l.	Middle Pleistocene	1		[10]
ZKD K1-96	Zhoukoudian (China)	*H. erectus* s.l.	Middle Pleistocene	1	1	[10]
ZKD L4-309	Zhoukoudian (China)	*H. erectus* s.l.	Middle Pleistocene	1		[10]
ZKD M3-310	Zhoukoudian (China)	*H. erectus* s.l.	Middle Pleistocene		1	[10]
ZKD O2-314	Zhoukoudian (China)	*H. erectus* s.l.	Middle Pleistocene	1		[10]
ZKD AN-518	Zhoukoudian (China)	*H. erectus* s.l.	Middle Pleistocene		1	[10]

PA69	Zhoukoudian (China)	*H. erectus* s.l.	Middle Pleistocene					1		[20]
PA70	Zhoukoudian (China)	*H. erectus* s.l.	Middle Pleistocene						1	[20]
PA102	Chenjiawo (China)	*H. erectus* s.l.	Middle Pleistocene					1		original data
PA531	Xichuan (China)	*H. erectus* s.l.	Middle Pleistocene					1		[20]
PA533	Xichuan (China)	*H. erectus* s.l.	Middle Pleistocene						1	[20]
ATD6-5	Atapuerca Gran Dolina (Spain)	*H. antecessor*	Early Pleistocene	1	1		1			[21, 22]
ATD6-94	Atapuerca Gran Dolina (Spain)	*H. antecessor*	Early Pleistocene	1		1				[21, 22]
ATD6-96	Atapuerca Gran Dolina (Spain)	*H. antecessor*	Early Pleistocene	1	1	1	1			[21, 22]
ATD6-112	Atapuerca Gran Dolina (Spain)	*H. antecessor*	Early Pleistocene			1				[21]

ATD6-113	Atapuerca Gran Dolina (Spain)	*H. antecessor*	Early Pleistocene		1		1			21, 22
ATD6-144	Atapuerca Gran Dolina (Spain)	*H. antecessor*	Early Pleistocene		1		1			21, 22
Xiahe	Baishiya Karst Cave (China)	Denisovan	Middle Pleistocene	1	1		1	1	1	23
HLD 1	Hualong Cave (China)	*Homo* sp.	Middle Pleistocene		1					24
HLD 6	Hualong Cave (China)	*Homo* sp.	Middle Pleistocene	2	2					24
Penghu 1	Penghu (Taiwan)	*Homo* sp.	Middle Pleistocene		1					25
AT-1	Atapuerca Sima de los Huesos (Spain)	Neanderthal	Middle Pleistocene	2	2					26
AT-2	Atapuerca Sima de los Huesos (Spain)	Neanderthal	Middle Pleistocene	1						26

AT-11	Atapuerca Sima de los Huesos (Spain)	Neanderthal	Middle Pleistocene		1	[26]
AT-14	Atapuerca Sima de los Huesos (Spain)	Neanderthal	Middle Pleistocene	1		[26]
AT-21	Atapuerca Sima de los Huesos (Spain)	Neanderthal	Middle Pleistocene	1		[26]
AT-22	Atapuerca Sima de los Huesos (Spain)	Neanderthal	Middle Pleistocene	1		[26]
AT-75	Atapuerca Sima de los Huesos (Spain)	Neanderthal	Middle Pleistocene		1	[26]
AT-101	Atapuerca Sima de los Huesos (Spain)	Neanderthal	Middle Pleistocene	1		[26]
AT-141	Atapuerca Sima de los Huesos (Spain)	Neanderthal	Middle Pleistocene	1		[26]

AT-142	Atapuerca Sima de los Huesos (Spain)	Neanderthal	Middle Pleistocene		1		[26]
AT-169	Atapuerca Sima de los Huesos (Spain)	Neanderthal	Middle Pleistocene		1	1	[21, 26]
AT-271	Atapuerca Sima de los Huesos (Spain)	Neanderthal	Middle Pleistocene		1	1	[21, 26]
AT-272	Atapuerca Sima de los Huesos (Spain)	Neanderthal	Middle Pleistocene	1			[26]
AT-273	Atapuerca Sima de los Huesos (Spain)	Neanderthal	Middle Pleistocene		1		[26]
AT-284	Atapuerca Sima de los Huesos (Spain)	Neanderthal	Middle Pleistocene		1	1	[21, 26]
AT-285	Atapuerca Sima de los Huesos (Spain)	Neanderthal	Middle Pleistocene	1			[26]

AT-286	Atapuerca Sima de los Huesos (Spain)	Neanderthal	Middle Pleistocene	1		[26]
AT-300	Atapuerca Sima de los Huesos (Spain)	Neanderthal	Middle Pleistocene	1	1	[26]
AT-505 + AT-604	Atapuerca Sima de los Huesos (Spain)	Neanderthal	Middle Pleistocene		2	[26]
AT-556	Atapuerca Sima de los Huesos (Spain)	Neanderthal	Middle Pleistocene	1		[26]
AT-557	Atapuerca Sima de los Huesos (Spain)	Neanderthal	Middle Pleistocene		1	[26]
AT-561	Atapuerca Sima de los Huesos (Spain)	Neanderthal	Middle Pleistocene	1		[26]
AT-576	Atapuerca Sima de los Huesos (Spain)	Neanderthal	Middle Pleistocene	1		[26]

AT-605	Atapuerca Sima de los Huesos (Spain)	Neanderthal	Middle Pleistocene	2	2			[26]
AT-607	Atapuerca Sima de los Huesos (Spain)	Neanderthal	Middle Pleistocene	2	2			[26]
AT-792	Atapuerca Sima de los Huesos (Spain)	Neanderthal	Middle Pleistocene	2	1			[26]
AT-793 + AT-250	Atapuerca Sima de los Huesos (Spain)	Neanderthal	Middle Pleistocene		2			[26]
AT-829	Atapuerca Sima de los Huesos (Spain)	Neanderthal	Middle Pleistocene	1		1		[21, 26]
AT-888	Atapuerca Sima de los Huesos (Spain)	Neanderthal	Middle Pleistocene	2	2			[26]
AT-941	Atapuerca Sima de los Huesos (Spain)	Neanderthal	Middle Pleistocene		1		1	[21,26]

AT-943	Atapuerca Sima de los Huesos (Spain)	Neanderthal	Middle Pleistocene	1			26
AT-946	Atapuerca Sima de los Huesos (Spain)	Neanderthal	Middle Pleistocene		1	1	21, 26
AT-950	Atapuerca Sima de los Huesos (Spain)	Neanderthal	Middle Pleistocene	2	2		26
AT-1458	Atapuerca Sima de los Huesos (Spain)	Neanderthal	Middle Pleistocene	1			26
AT-1459	Atapuerca Sima de los Huesos (Spain)	Neanderthal	Middle Pleistocene	1			26
AT-1752	Atapuerca Sima de los Huesos (Spain)	Neanderthal	Middle Pleistocene		1		26
AT-1756	Atapuerca Sima de los Huesos (Spain)	Neanderthal	Middle Pleistocene		1		26

AT-1759	Atapuerca Sima de los Huesos (Spain)	Neanderthal	Middle Pleistocene	1				[26]
AT-1761	Atapuerca Sima de los Huesos (Spain)	Neanderthal	Middle Pleistocene		1		1	[21, 26]
AT-1775	Atapuerca Sima de los Huesos (Spain)	Neanderthal	Middle Pleistocene	1				[26]
AT-1957	Atapuerca Sima de los Huesos (Spain)	Neanderthal	Middle Pleistocene	1	1			[26]
AT-2193	Atapuerca Sima de los Huesos (Spain)	Neanderthal	Middle Pleistocene	1	1			[26]
AT-2270	Atapuerca Sima de los Huesos (Spain)	Neanderthal	Middle Pleistocene		1		1	[21, 26]
AT-2272	Atapuerca Sima de los Huesos (Spain)	Neanderthal	Middle Pleistocene		1			[26]

AT-2276	Atapuerca Sima de los Huesos (Spain)	Neanderthal	Middle Pleistocene	1				[26]
AT-2396	Atapuerca Sima de los Huesos (Spain)	Neanderthal	Middle Pleistocene		1		1	[21,26]
AT-2438	Atapuerca Sima de los Huesos (Spain)	Neanderthal	Middle Pleistocene	1	1			[26]
AT-2763	Atapuerca Sima de los Huesos (Spain)	Neanderthal	Middle Pleistocene		1			[26]
AT-2779	Atapuerca Sima de los Huesos (Spain)	Neanderthal	Middle Pleistocene	1				[26]
AT-3175	Atapuerca Sima de los Huesos (Spain)	Neanderthal	Middle Pleistocene	1				[21,26]
AT-3176	Atapuerca Sima de los Huesos (Spain)	Neanderthal	Middle Pleistocene		1			[26]

AT-3179	Atapuerca Sima de los Huesos (Spain)	Neanderthal	Middle Pleistocene		1	1	[21,26]
AT-3889	Atapuerca Sima de los Huesos (Spain)	Neanderthal	Middle Pleistocene		1		[26]
AT-3890	Atapuerca Sima de los Huesos (Spain)	Neanderthal	Middle Pleistocene		1		[26]
AT-3933	Atapuerca Sima de los Huesos (Spain)	Neanderthal	Middle Pleistocene	1			[26]
AT-3934	Atapuerca Sima de los Huesos (Spain)	Neanderthal	Middle Pleistocene	1			[26]
AT-4147	Atapuerca Sima de los Huesos (Spain)	Neanderthal	Middle Pleistocene	1	1		[26]
AT-4318	Atapuerca Sima de los Huesos (Spain)	Neanderthal	Middle Pleistocene	1			[26]

AT-6579	Atapuerca Sima de los Huesos (Spain)	Neanderthal	Middle Pleistocene		1			[26]
Fontana Ranuccio IV	Fontana Ranuccio (Italy)	Neanderthal	Middle Pleistocene			1		[27]
Montmaurin	Montmaurin (France)	Neanderthal	Middle Pleistocene			2	2	[28]
S5	La Chaise-de-Vouthon Abri Suard (France)	Neanderthal	Middle Pleistocene			1		[12]
S36	La Chaise-de-Vouthon Abri Suard (France)	Neanderthal	Middle Pleistocene		1		1	[29,30]
S49	La Chaise-de-Vouthon Abri Suard (France)	Neanderthal	Middle Pleistocene			1		[12]
S14-7	La Chaise-de-Vouthon Abri Suard (France)	Neanderthal	Middle Pleistocene	1		1		[29,30]

Archi 1	Archi (Italy)	Neanderthal	Late Pleistocene				1		original data
BD-J4-C9	La Chaise-de-Vouthon Abri Bourgeois-Delaunay (France)	Neanderthal	Late Pleistocene		1		1		29,30
GB 78 B13 C65 66	Grotte Boccard (France)	Neanderthal	Late Pleistocene	1					31
Combe Grenal I	Combe Grenal (France)	Neanderthal	Late Pleistocene				1		32
Combe Grenal IV	Combe Grenal (France)	Neanderthal	Late Pleistocene		1				29,31
Combe Grenal VI	Combe Grenal (France)	Neanderthal	Late Pleistocene				1		32
KRD1	Krapina (Croatia)	Neanderthal	Late Pleistocene			1		1	12
KRD6	Krapina (Croatia)	Neanderthal	Late Pleistocene	1		1		1	29,31

KRD9	Krapina (Croatia)	Neanderthal	Late Pleistocene					1	[32]
KRD10	Krapina (Croatia)	Neanderthal	Late Pleistocene			1		1	[29,32]
KRD77	Krapina (Croatia)	Neanderthal	Late Pleistocene				1		[12]
KRD79	Krapina (Croatia)	Neanderthal	Late Pleistocene				1		[12,32]
KRD80	Krapina (Croatia)	Neanderthal	Late Pleistocene				1		[12,32]
KRD81	Krapina (Croatia)	Neanderthal	Late Pleistocene				1		[12,32]
KRD86	Krapina (Croatia)	Neanderthal	Late Pleistocene		1			1	[12,18,32]
KRD105	Krapina (Croatia)	Neanderthal	Late Pleistocene	1			1		[12,18,32]
KRD107	Krapina (Croatia)	Neanderthal	Late Pleistocene		1			1	[12,18,32]

KRP52	Krapina (Croatia)	Neanderthal	Late Pleistocene					1		[32]
KRP53	Krapina (Croatia)	Neanderthal	Late Pleistocene			1		1	1	[29,32]
KRP54	Krapina (Croatia)	Neanderthal	Late Pleistocene			1		1	1	[29,32,33]
KRP55	Krapina (Croatia)	Neanderthal	Late Pleistocene			1		1	1	[32-35]
KRP57	Krapina (Croatia)	Neanderthal	Late Pleistocene						1	[32]
KRP59	Krapina (Croatia)	Neanderthal	Late Pleistocene						1	[32]
Krapina 80	Krapina (Croatia)	Neanderthal	Late Pleistocene	1			1			[31,33]
Krapina D/D	Krapina (Croatia)	Neanderthal	Late Pleistocene	1	1					[31]
Krapina F/H	Krapina (Croatia)	Neanderthal	Late Pleistocene	2	2					[31]

Krapina MND C	Krapina (Croatia)	Neanderthal	Late Pleistocene	1	1	[31]
Krapina MND E	Krapina (Croatia)	Neanderthal	Late Pleistocene	2	2	[31]
Krapina MND G	Krapina (Croatia)	Neanderthal	Late Pleistocene	1	1	[31]
Krapina MND J	Krapina (Croatia)	Neanderthal	Late Pleistocene	1	1	[31]
Krapina MND M	Krapina (Croatia)	Neanderthal	Late Pleistocene	1	1	[31]
Krapina MND P	Krapina (Croatia)	Neanderthal	Late Pleistocene	1		[31]
Krapina MND Q	Krapina (Croatia)	Neanderthal	Late Pleistocene	1		[31]
Krapina N/N	Krapina (Croatia)	Neanderthal	Late Pleistocene	1	1	[31]
Krapina R64/MND L	Krapina (Croatia)	Neanderthal	Late Pleistocene	1	1	[31]

Ehringsdorf I	Ehringsdorf (Germany)	Neanderthal	Late Pleistocene					1		[12]
Engis 1	Engis (Belgium)	Neanderthal	Late Pleistocene			1		1		[29], original data
Fossellone 3	Fossellone (Italy)	Neanderthal	Late Pleistocene	1	1			1	1	[31], original data
Gibraltar 2	Devil's Tower (Gibraltar)	Neanderthal	Late Pleistocene					1		[19]
Montgaudier	Montgaudier (France)	Neanderthal	Late Pleistocene	1						[31]
La Quina H9 Q760	La Quina (France)	Neanderthal	Late Pleistocene	1	1				1	[18,32]
Las Palomas 29	Las Palomas (Spain)	Neanderthal	Late Pleistocene				1			[36]
Le Moustier 1	Le Moustier (France)	Neanderthal	Late Pleistocene				1	1	1	[18,32]
Molare 1	Molare (Italy)	Neanderthal	Late Pleistocene					1		original data

Roc de Marsal 1	Roc de Marsal (France)	Neanderthal	Late Pleistocene			2		1		[29,30,34]
Regourdou 1	Regourdou (France)	Neanderthal	Late Pleistocene				1		1	[33,37]
Scladina 4A1	Scladina (Belgium)	Neanderthal	Late Pleistocene					1	1	[32]
SD-540	El Sidrón (Spain)	Neanderthal	Late Pleistocene			1			1	[29,30,32]
SD-755	El Sidrón (Spain)	Neanderthal	Late Pleistocene			1			1	[29,30,32]
SD-756	El Sidrón (Spain)	Neanderthal	Late Pleistocene					1		[32]
SD-780	El Sidrón (Spain)	Neanderthal	Late Pleistocene					1		[32]
Vi 11-39	Vindija (Croatia)	Neanderthal	Late Pleistocene					1		[32]
Amud 1	Amud (Israel)	Neanderthal	Late Pleistocene	2	2					[31]

Kebara 2	Kebara (Israel)	Neanderthal	Late Pleistocene	2	2					[31]
Shovakh 1	Me'arat Shovakh (Israel)	Neanderthal	Late Pleistocene		1					[31]
Shanidar 1	Shanidar (Iraq)	Neanderthal	Late Pleistocene	2						[31]
Shanidar 2	Shanidar (Iraq)	Neanderthal	Late Pleistocene	2						[31]
Lagar Velho 1	Lagar Velho (Portugal)	modern human	Late Pleistocene					1		[38]
Qafzeh 4	Qafzeh (Israel)	modern human	Late Pleistocene					2		original data
Qafzeh 7	Qafzeh (Israel)	modern human	Late Pleistocene		2					[31]
Qafzeh 8	Qafzeh (Israel)	modern human	Late Pleistocene		1					[31]
Qafzeh 9	Qafzeh (Israel)	modern human	Late Pleistocene	1	2			1	2	[31,32]

Qafzeh 10	Qafzeh (Israel)	modern human	Late Pleistocene			1		[31,32]
Qafzeh11	Qafzeh (Israel)	modern human	Late Pleistocene			1	1	[32]
Qafzeh15	Qafzeh (Israel)	modern human	Late Pleistocene			2		[19,32]
Mladeč 52	Mladeč (Czech Republic)	modern human	Late Pleistocene	2	2			[31]
Mladeč 54	Mladeč (Czech Republic)	modern human	Late Pleistocene	2	2			[31]
Pavlov 1	Pavlov (Czech Republic)	modern human	Late Pleistocene	1	2			[31]
Předmost 1	Předmost (Czech Republic)	modern human	Late Pleistocene	2	2			[31]
Předmost 3	Předmost (Czech Republic)	modern human	Late Pleistocene	2	1			[31]
Předmost 4	Předmost (Czech Republic)	modern human	Late Pleistocene	2	2			[31]

Předmost 5	Předmost (Czech Republic)	modern human	Late Pleistocene	2	2	[31]
Předmost 7	Předmost (Czech Republic)	modern human	Late Pleistocene	1	2	[31]
Předmost 9	Předmost (Czech Republic)	modern human	Late Pleistocene	2	2	[31]
Předmost 10	Předmost (Czech Republic)	modern human	Late Pleistocene	2	2	[31]
Předmost 14	Předmost (Czech Republic)	modern human	Late Pleistocene	2	1	[31]
Předmost 18	Předmost (Czech Republic)	modern human	Late Pleistocene	2	1	[31]
Předmost 26	Předmost (Czech Republic)	modern human	Late Pleistocene	1	1	[31]
Předmost 27	Předmost (Czech Republic)	modern human	Late Pleistocene	2	2	[31]

Předmost 259	Předmost (Czech Republic)	modern human	Late Pleistocene	1		31
Předmost 476	Předmost (Czech Republic)	modern human	Late Pleistocene	2	1	31
Předmost 3070	Předmost (Czech Republic)	modern human	Late Pleistocene	1		31
Abri Pataud P1	Abri Pataud (France)	modern human	Late Pleistocene	2	2	31
Cap Blanc 1	Cap Blanc (France)	modern human	Late Pleistocene	2	2	31
Les Rois A	Les Rois (France)	modern human	Late Pleistocene	2		31
R50.31	Les Rois (France)	modern human	Late Pleistocene		1	31
R51.6	Les Rois (France)	modern human	Late Pleistocene		1	31
R51.14	Les Rois (France)	modern human	Late Pleistocene		1	31

R51.16	Les Rois (France)	modern human	Late Pleistocene	1		31
55.148b	Les Rois (France)	modern human	Late Pleistocene	1	1	31
Les Vachons 1	Les Vachons (France)	modern human	Late Pleistocene	1	1	31
L.B. II	Grotte de la Balauzière (France)	modern human	Late Pleistocene	1		31
L.B. III	Grotte de la Balauzière (France)	modern human	Late Pleistocene	1		31
L.B. IV	Grotte de la Balauzière (France)	modern human	Late Pleistocene	1		31
L.B. VIII	Grotte de la Balauzière (France)	modern human	Late Pleistocene	1		31
Mas d'Azil 900	Mas d'Azil (France)	modern human	Late Pleistocene	2		31
Mas d'Azil 901	Mas d'Azil (France)	modern human	Late Pleistocene		1	31

La Madeleine 24835	La Madeleine (France)	modern human	Late Pleistocene	1	1	31
B11970.8	Saint Germain La Rivière (France)	modern human	Late Pleistocene		1	31
B31970.8	Saint Germain La Rivière (France)	modern human	Late Pleistocene	2		31
B41970.8b	Saint Germain La Rivière (France)	modern human	Late Pleistocene	2	1	31
SGLR 4	Saint Germain La Rivière (France)	modern human	Late Pleistocene	1	4	31
Arene Candide 1	Arene Candide (Italy)	modern human	Late Pleistocene	1	1	31
Arene Candide 2	Arene Candide (Italy)	modern human	Late Pleistocene	1	2	31
Arene Candide 4	Arene Candide (Italy)	modern human	Late Pleistocene	1	2	31

Arene Candide 5	Arene Candide (Italy)	modern human	Late Pleistocene	1	2	31
Arene Candide 20	Arene Candide (Italy)	modern human	Late Pleistocene	2	2	31
Arene Candide 19.6725	Arene Candide (Italy)	modern human	Late Pleistocene	2	2	31
Barma Grande 2	Barma Grande (Italy)	modern human	Late Pleistocene	2	2	31
Barma Grande 4	Barma Grande (Italy)	modern human	Late Pleistocene	2	2	31
Barma Grande 5	Barma Grande (Italy)	modern human	Late Pleistocene	1	1	31
Grotte des Enfants 4	Grotte des Enfants (Italy)	modern human	Late Pleistocene		2	31
Grotte des Enfants 6	Grotte des Enfants (Italy)	modern human	Late Pleistocene	2	2	31

Paglicci 12	Paglicci (Italy)	modern human	Late Pleistocene	2	2	31
PA20.6.B	Paglicci (Italy)	modern human	Late Pleistocene		1	31
Paglicci 20.d.G	Paglicci (Italy)	modern human	Late Pleistocene	1		31
Paglicci C	Paglicci (Italy)	modern human	Late Pleistocene		1	31
Romanelli R7	Romanelli (Italy)	modern human	Late Pleistocene	1	1	31
Romanelli R8	Romanelli (Italy)	modern human	Late Pleistocene	1	2	31
Romito 1	Romito (Italy)	modern human	Late Pleistocene	2	2	31
Romito 2	Romito (Italy)	modern human	Late Pleistocene	1	1	31
Romito 3	Romito (Italy)	modern human	Late Pleistocene	1	1	31

Romito 4	Romito (Italy)	modern human	Late Pleistocene	2	2					[31]
Romito 6	Romito (Italy)	modern human	Late Pleistocene	2	2					[31]
Holocene humans	worldwide	modern human	Holocene	174	204	12	26	33	41	[17,31,32,39]

Supplementary Table 10. Tooth crown metrics of TNH2-1 compared with fossil and extant human M1 and M2 samples. MD: mesiodistal diameter (mm); BL: buccolingual diameter (mm); CCA: calculated crown area (BL*MD; mm^2); CI: crown index (BL/MD*100; %). HE: *H. erectus* s.l.; HA: *H. antecessor*; EMPH: European Middle Pleistocene *Homo*; AMPH: Asian Middle Pleistocene *Homo*; NEA: Neanderthals; UPMH: Upper Palaeolithic modern humans; HH: Holocene humans.

		**MD**	**BL**	**CCA**	**CI**
TNH2-1		13.20	11.30	149.16	0.86
M1					
HE (n=31)	mean	12.61	11.76	148.71	0.93
	s.d.	0.86	0.83	18.77	0.05
HA (n=3)	mean	11.90	11.53	137.63	0.99
	s.d.	1.28	0.46	19.74	0.07
EMPH (n=41)	mean	11.21	10.46	117.47	0.93
	s.d.	0.52	0.49	10.09	0.03
AMPH (n=3)	mean	13.53	11.50	156.43	0.85

	s.d.	1.38	0.87	28.28	0.02
NEA (n=25)	mean	11.80	11.13	131.83	0.95
	s.d.	0.91	0.73	18.05	0.04
UPMH (n=79)	mean	11.45	10.88	124.70	0.95
	s.d.	0.70	0.70	14.16	0.05
HH (n=174)	mean	11.40	10.96	124.87	0.96
	s.d.	0.63	0.48	11.10	0.04
**M2**					
HE (n=35)	mean	12.49	11.77	148.00	0.94
	s.d.	1.15	1.04	25.17	0.05
HA (n=4)	mean	13.15	11.55	152.05	0.88
	s.d.	0.66	0.41	12.19	0.03
EMPH (n=41)	mean	11.04	10.22	113.15	0.93

	s.d.	0.57	0.66	12.34	0.03
AMPH (n=5)	mean	13.32	11.90	160.16	0.89
	s.d.	1.09	1.90	38.14	0.07
NEA (n=31)	mean	12.08	11.29	136.75	0.94
	s.d.	0.92	0.66	17.40	0.05
UMPH (n=81)	mean	11.03	10.71	118.29	0.98
	s.d.	0.84	0.80	16.48	0.06
HH (n=204)	mean	10.73	10.54	113.38	0.98
	s.d.	0.62	0.58	11.74	0.04

Supplementary Table 11. Statistics of the tooth crown diameters. Adjusted Z-score analyses of the mesiodistal and buccolingual of TNH2-1 compared with Pleistocene and Holocene human samples. The values above 1 or below -1, representing those outside of the 95% confidence interval of the comparative group variability, are highlighted in bold. MD: mesiodistal diameter (mm); BL: buccolingual diameter (mm); CCA: calculated crown area (BL*MD; mm^2); CI: crown index (BL/MD*100; %). HE: *H. erectus* s.l.; HA: *H. antecessor*; EMPH: European Middle Pleistocene *Homo*; AMPH: Asian Middle Pleistocene *Homo*; NEA: Neanderthals; UPMH: Upper Palaeolithic modern humans; HH: Holocene humans.

	**MD**	**BL**	**CCA**	**CI**
**M1**				
TNH2-1 vs. HE	0.33	-0.27	0.01	-0.67
TNH2-1 vs. HA	0.20	-0.10	0.12	-0.37
TNH2-1 vs. EMPH	**1.87**	0.84	**1.54**	**-1.14**
TNH2-1 vs. AMPH	-0.05	-0.05	-0.05	0.10
TNH2-1 vs. NEA	0.73	0.11	0.46	**-1.07**
TNH2-1 vs. UPMH	**1.25**	0.30	0.86	-0.90
TNH2-1 vs. HH	**1.44**	0.36	**1.11**	**-1.26**
**M2**				
TNH2-1 vs. HE	0.30	-0.22	0.02	-0.78
TNH2-1 vs. HA	0.02	-0.17	-0.07	-0.19

TNH2-1 vs. EMPH	**1.85**	0.80	**1.43**	**-1.14**
TNH2-1 vs. AMPH	-0.04	-0.10	-0.09	-0.14
TNH2-1 vs. NEA	0.59	0.01	0.34	-0.77
TNH2-1 vs. UPMH	**1.29**	0.37	0.94	**-1.00**
TNH2-1 vs. HH	**2.02**	0.66	**1.54**	**-1.49**

Supplementary Table 12. Tooth crown tissue proportions of TNH2-1 compared with fossil and extant human M1 and M2 samples. Vcdp/Vc: percent of the crown volume that is dentine and pulp (%); 3D AET: three-dimensional average enamel thickness (mm); 3D RET: three-dimensional relative enamel thickness. HE: *H. erectus* s.l.; HA: *H. antecessor*; EMPH: European Middle Pleistocene *Homo*; AMPH: Asian Middle Pleistocene *Homo*; NEA: Neanderthals; HH: Holocene humans.

		Vcdp/Vc	3D AET	3D RET
TNH2-1		55.37	1.18	17.00
**M1**				
HE (n=1)		55.96	1.02	14.74
HA (n=3)	mean	56.89	1.13	16.16
	s.d.	2.80	0.13	1.43
EMPH (n=1)		54.23	1.11	17.42
NEA (n=11)	mean	59.06	1.12	16.02
	s.d.	3.02	0.23	3.63
HH (n=12)	mean	53.40	1.09	16.67
	s.d.	3.00	0.12	4.97
**M2**				
HE (n=8)	mean	50.70	1.33	20.09
	s.d.	3.84	0.10	2.80

HA (n=4)	mean	51.70	1.27	19.85
	s.d.	6.60	0.09	4.26
EMPH (n=10)	mean	49.67	1.31	22.20
	s.d.	2.62	0.10	6.53
AMPH (n=1)		54.62	1.47	18.97
NEA (n=10)	mean	58.19	1.10	15.67
	s.d.	4.51	0.19	5.49
HH (n=26)	mean	51.11	1.28	20.11
	s.d.	3.63	0.33	7.27

Supplementary Table 13. Statistics of the tooth crown tissue proportions. Adjusted Z-score analyses of the tissue proportions of TNH2-1 compared with Pleistocene and Holocene human samples. The values above 1 or below -1, representing those outside of the 95% confidence interval of the comparative group variability, are highlighted in bold. Vcdp/Vc: percent of the crown volume that is dentine and pulp (%); 3D AET: three-dimensional average enamel thickness (mm); 3D RET: three-dimensional relative enamel thickness. HE: *H. erectus* s.l.; HA: *H. antecessor*; EMPH: European Middle Pleistocene *Homo*; NEA: Neanderthals; HH: Holocene humans.

	**Vcdp/Vc**	**3D AET**	**3D RET**
**M1**			
TNH2-1 vs. HA	-0.11	0.08	0.12
TNH2-1 vs. NEA	-0.52	0.12	0.12
TNH2-1 vs. HH	0.29	0.31	0.03
**M2**			
TNH2-1 vs. HE	0.41	-0.69	-0.37
TNH2-1 vs. HA	0.16	-0.29	-0.19
TNH2-1 vs. EMPH	0.92	-0.53	-0.34
TNH2-1 vs. NEA	-0.26	0.18	0.10
TNH2-1 vs. HH	0.56	-0.14	-0.20

Supplementary Table 14. Procedural tests for pIR-IRSL for single-grains of feldspar.

**Procedural Tests**	(pIR-IRSL$_{50,225}$)	(pIR-IRSL$_{50,250}$)	(pIR-IRSL$_{50,270}$)	(pIR-IRSL$_{50,290}$)	(pIR-IRSL$_{200,290}$)
Preheat range (Gy)	83	80	55	287	203
Dose recovery ratio	0.945	0.975	0.995	1.20	1.25
Fading SG % per decade	3.84	4.61	2.32	2.56	2.78
Fading SA % per decade	2.5	2.2	1.70	1.50	1.51
Residuals (Gy)	7	8.5	9	21	12
Recuperation %	4.4 ± 0.4	2.2 ± 0.12	0.78 ± 0.22	2.4 ± 0.12	2.6 ± 0.24
Recycling ratio	1.22 ± 0.23	1.02 ± 0.23	1.04 ± 0.03	0.88 ± 0.04	1.34 ± 0.21
Average D0 (Gy)	820 ± 72	780 ± 68	1023 ± 102	978 ± 89	823 ± 76

Supplementary Table 15: Rejection criteria for feldspar grains. Based on rejection criteria from (*40*).

**Rejections**	**LCC1**	**LCC2**	**LCC3**
**Total grains run**	3000	3000	2700
**Accepted**	48	25	23
**Poor signals <3 >sd bg**[a]	2578	2200	1832
**Recycling ratios > or < 0.8-1.2**[b]	254	456	564
**Super saturated**[c]	40	23	79
**Recuperation >5 %**[d]	24	50	24
**De = 1 sig 0**[e]	56	246	178

[a] Net natural signals less than three times above the standard deviation of the background
[b] Recycling ratios outside of the range 0.8-1.2,
[c] Yielded natural signals that did not intersect the regeneration growth curves,
[d] Signal larger than 10% of the natural signal after a zero dose
[e] Produced a De within 1-sigma of zero.

Supplementary Table 16. Results of the canonical variates analyses (CVA). The cross-validated (CV) CVA shows good predictive accuracy between the first and second molar (M1 and M2) of the combined dataset of *H. erectus*, Neanderthal and modern human teeth. The posterior probabilities of the three teeth projected a posteriori in the EDJ shape analyses (TNH2-1, Xiahe M1 and M2) were then calculated.

	**CV correct classification**	**Posterior probability**
Landmark-based analyses	M1: 89.7% ; M2: 93.3	TNH2-1: 100.0% M2<br>Xiahe M1: 65.0% M1<br>Xiahe M2: 60.0% M1
Deformation-based analyses	M1: 89.9% ; M2: 90.5%	TNH2-1: 97.0% M2<br>Xiahe M1: 99.0% M2<br>Xiahe M2: 86.0% M2

Supplementary Table 17. Results of the canonical variates analyses (CVA). The cross-validated (CV) CVA shows excellent predictive accuracy, with a clear discrimination between *H. erectus* s.l. (HE), Middle and Late Pleistocene Neanderthals (NEA), and Pleistocene and Holocene modern humans (MH). The posterior probabilities of the three teeth projected a posteriori in the EDJ shape analyses (TNH2-1, Xiahe M1 and M2) were then calculated.

	**CV correct classification**	**Posterior probability**
Landmark-based analyses	HE: 94.1%; NEA: 100.0%;<br>MH: 97.6%	TNH2-1: 100% NEA<br>Xiahe M1: 100% NEA<br>Xiahe M2: 82% NEA
Deformation-based analyses	HE: 100.0%; NEA: 100.0%;<br>MH: 97.6%	TNH2-1: 100% NEA<br>Xiahe M1: 96% NEA<br>Xiahe M2: 100% HE

Supplementary References

1. Patole-Edoumba, E. *et al.* Evolution of the Hoabinhian techno-complex of Tam Hang rock shelter in northeastern Laos. *Archaeol. Discov.* **3**, 140–157 (2015).

2. Patole-Edoumba, E. & Demeter, F. *Pa Hang: La montagne habitée*. (Indes Savantes, 2018).

3. Saurin, E. *Carte geologique du Laos, feuille Luang Prabang*. (Publication du service geographique national du Vietnam, 1961).

4. Duringer, P., Bacon, A. M., Sayavongkhamdy, T. & Nguyen, T. K. T. Karst development, breccias history, and mammalian assemblages in Southeast Asia: A brief review. *Comptes Rendus Palevol* **11**, 133–157 (2012).

5. Shao, Q., Bahain, J. J., Falguères, C., Dolo, J. M. & Garcia, T. A new U-uptake model for combined ESR/U-series dating of tooth enamel. *Quat. Geochronol.* **10**, 406–411 (2012).

6. Guerin, G., Mercier, N. & Adamiec, G. Dose rate conversion factors: update. *Anc. TL* **29**, 5–11 (2011).

7. Grun, R. Beta dose attenuation in thin layers. *Anc. TL* **4**, 1–8 (1986).

8. Lamothe, M., Auclair, M. H. C. & S., H. Towards a prediction of long-term anomalous fading of feldspar IRSL. *Radiat. Meas.* **37**, 493–498 (2003).

9. Welker, F. *et al.* The dental proteome of Homo antecessor. *Nature* **580**, 235-238 (2020)

10. Wood, B. A. *Koobi Fora Research Project, Volume 4. Hominid Cranial Remains*. (Clarendon Press, 1991).

11. Zanolli, C. & Mazurier, A. Endostructural characterization of the *H. heidelbergensis* dental remains from the early Middle Pleistocene site of Tighenif, Algeria. *Comptes Rendus Palevol* **12**, 293-304 (2013).

12. Zanolli, C. *et al.* The late Early Pleistocene human dental remains from Uadi Aalad and Mulhuli-Amo (Buia), Eritrean Danakil: Macromorphology and microstructure. *J. Hum. Evol.* **74**, 96–113 (2014).

13. Zanolli, C. *et al.* Evidence for increased hominid diversity in the Early to Middle Pleistocene of Indonesia. *Nat. Ecol. Evol.* **3**, 755–764 (2019).

14. Grine, F. E. & Franzen, J. L. Fossil hominid teeth from the Sangiran Dome (Java, Indonesia). *Cour Forsch-Inst Senckenb.* **171**, 75–103 (1994).

15. Kaifu, Y. *et al.* Taxonomic affinities and evolutionary history of the Early Pleistocene hominids of Java: Dentognathic evidence. *Am. J. Phys. Anthropol.* **128**, 709-726 (2005).

16. Zanolli, C. Additional evidence for morpho-dimensional tooth crown variation in a new Indonesian *H. erectus* sample from the Sangiran Dome (Central Java). *PLoS One* **8**, e67233 (2013).

17. Zanolli, C. Molar crown inner structural organization in Javanese *Homo erectus*. *Am. J. Phys. Anthropol.* **156**, 148–157 (2015).

18. Smith, T. M. *et al.* Disentangling isolated dental remains of Asian Pleistocene hominins and pongines. *PLoS One* **13**, e0204737 (2018).

19. ESRF Paleontological Microtomographic database. (2018).

20. Xing, S., Martinón-Torres, M. & Bermúdez De Castro, J. M. The fossil teeth of the Peking Man. *Sci. Rep.* **8**, 2066 (2018).

21. Martin-Frances, L. *et al.* Tooth crown tissue proportions and enamel thickness in Early Pleistocene *Homo antecessor* molars (Atapuerca, Spain). *PLoS One* **13**, e0203334 (2018).

22. Martinón-Torres, M. *et al.* New permanent teeth from Gran Dolina-TD6 (Sierra de Atapuerca). The bearing of *Homo antecessor* on the evolutionary scenario of Early and Middle Pleistocene Europe. *J. Hum. Evol.* **127**, 93-117 (2019).

23. Chen, F. *et al.* A late Middle Pleistocene Denisovan mandible from the Tibetan Plateau. *Nature* **569**, 409–412 (2019).

24. Wu, X. J. *et al.* Archaic human remains from Hualongdong, China, and Middle Pleistocene human continuity and variation. *Proc. Natl. Acad. Sci. U. S. A.* **116**, 9820–9824 (2019).

25. Chang, C. H. *et al.* The first archaic *Homo* from Taiwan. *Nat. Commun.* **6**, 1–33 (2015).

26. Martinón-Torres, M., Bermúdez De Castro, J. M., Gómez-Robles, A., Prado-Simón, L. & Arsuaga, J. L. Morphological description and comparison of the dental remains from Atapuerca-Sima de los Huesos site (Spain). *J. Hum. Evol.* **62**, 7-58 (2012).

27. Zanolli, C. *et al.* The Middle Pleistocene (MIS 12) human dental remains from Fontana Ranuccio (Latium) and Visogliano (Friuli-Venezia Giulia), Italy. A comparative high resolution endostructural assessment. *PLoS One* **13**, e0189773 (2018).

28. Martínez de Pinillos, M. *et al.* Inner morphological and metric characterization of the molar remains from the Montmaurin-La Niche mandible: The Neanderthal signal. *J. Hum. Evol.* 145, 102739 (2020).

29. Olejniczak, A. J. *et al.* Dental tissue proportions and enamel thickness in Neandertal and modern human molars. *J. Hum. Evol.* **55**, 12–23 (2008).

30. Macchiarelli, R. *et al.* How Neanderthal molar teeth grew. *Nature* **444**, 748-751 (2006).

31. Voisin, J.-L., Condemi, S., Wolpoff, M. H. & Frayer, D. W. A new online database (http://anthropologicaldata.free.fr) and a short reflection about the productive use of compiling internet data. *PaleoAnthropology* **2012**, 241-244 (2012).

32. Skinner, M. M. *et al.* A dental perspective on the taxonomic affinity of the Balanica mandible (BH-1). *J. Hum. Evol.* **93**, 63–81 (2016).

33. Kupczik, K. & Hublin, J. J. Mandibular molar root morphology in Neanderthals and Late Pleistocene and recent *Homo sapiens*. *J. Hum. Evol.* **59**, 525-541 (2010).

34. Bayle, P., Braga, J., Mazurier, A. & Macchiarelli, R. Dental developmental pattern of the Neanderthal child from Roc de Marsal: a high-resolution 3D analysis. *J. Hum. Evol.* **56**, 66-75 (2009).

35. Duval, M. & Grün, R. Are published ESR dose assessments on fossil tooth enamel reliable? *Quat. Geochronol.* **31**, 19–27 (2016).

36. Bayle, P., M., L. L. & Robson Brown, K. A. in *The People of Palomas - Neandertals from the Sima de los Palomas del Cabezo Gordo, Southeastern Spain* (Trinkaus, E. & Walker, M. J., eds) 115–137 (Texas A&M University Press, 2017).

37. Macchiarelli, R., Bayle, P., Bondioli, L., Mazurier, A. & Zanolli, C. in *Anthropological Perspectives on Tooth Morphology: Genetics, Evolution, Variation* (eds. Scott, R. G. & Irish, J. D.) 250–277 (Cambridge University Press, 2013).

38. Bayle, P. *et al.* Dental maturational sequence and dental tissue proportions in the early Upper Paleolithic child from Abrigo do Lagar Velho, Portugal. *Proc. Natl. Acad. Sci. U. S. A.* **107**, 1338-1342 (2010).

39. Olejniczak, A. J., Tafforeau, P., Feeney, R. N. M. & Martin, L. B. Three-dimensional primate molar enamel thickness. *J. Hum. Evol.* **54**, 187-195 (2008).

40. Jacobs, Z., Duller, G. A. T. & Wintle, A. G. Interpretation of single grain Dedistributions and calculation of De. *Radiat. Meas.* **41**, 264–277 (2006).
