# Supplementary material for: A Middle Pleistocene Denisovan molar from the Annamite Chain of northern Laos
Source: Nat Commun. 2022 May 17;13:2557. doi: 10.1038/s41467-022-29923-z (PMC9114389; doi:10.1038/s41467-022-29923-z)
Supplement: Supplementary file 3 — Description of Additional Supplementary Files [file 41467_2022_29923_MOESM3_ESM.pdf]

Description of Additional Supplementary Files

Supplementary Data 1 | Annotated HCD fragmentation spectrum using the "Interactive Peptide Spectral Annotator" web tool with 10 ppm mass accuracy.

Supplementary Data 2 | Measurements of all faunal and hominin teeth included in the analyses in an xsls file with two tabs.
