# Supplementary material for: A Middle Pleistocene Denisovan molar from the Annamite Chain of northern Laos
Source: Nat Commun. 2022 May 17;13:2557. doi: 10.1038/s41467-022-29923-z (PMC9114389; doi:10.1038/s41467-022-29923-z)
Supplement: Supplementary file 4 — Supplementary Data 1 [file 41467_2022_29923_MOESM4_ESM.pdf]

Supplementary Data 1 | Annotated HCD fragmentation spectrum. Fragments were annotated using the "Interactive Peptide Spectral Annotator" web tool with 10 ppm mass accuracy. Spectra can be identified by raw file ("Sample") and scan number. Score refers to the Andromeda score. Fragment types are color coded: red (y), blue (b), green (c), purple (a), orange (z). Lower case letters represent modified amino acids: Deamidation (qn), Oxidation (m), Hydroxyproline (p), Arginine to Ornithine (r), and phosphorylation (s). Neutral losses were only annotated for water and ammonium, but not for phosphate losses.

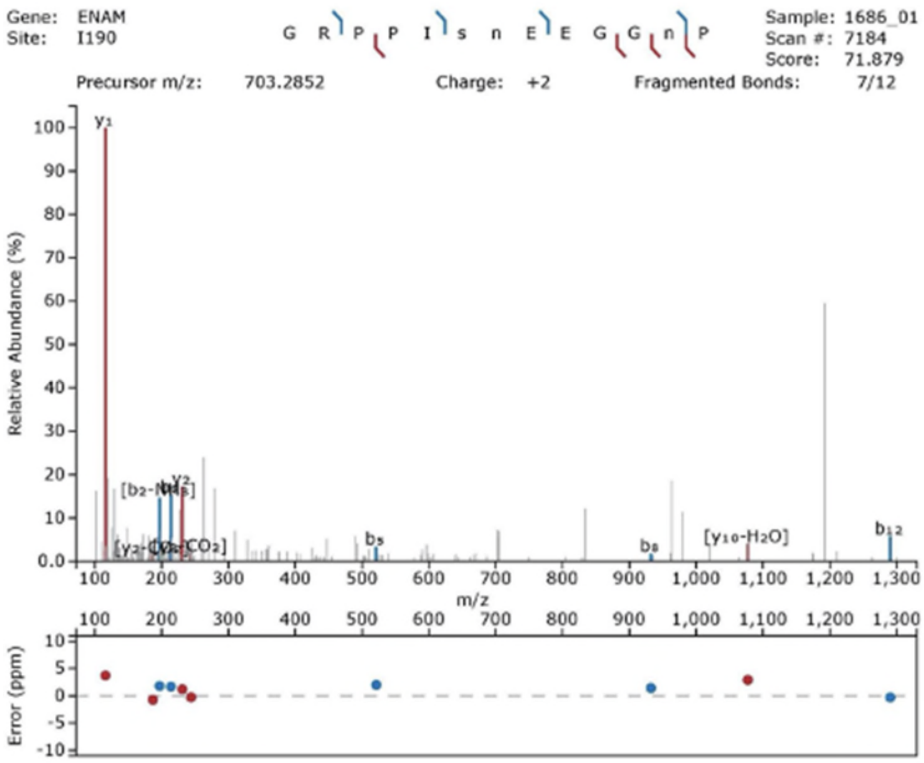

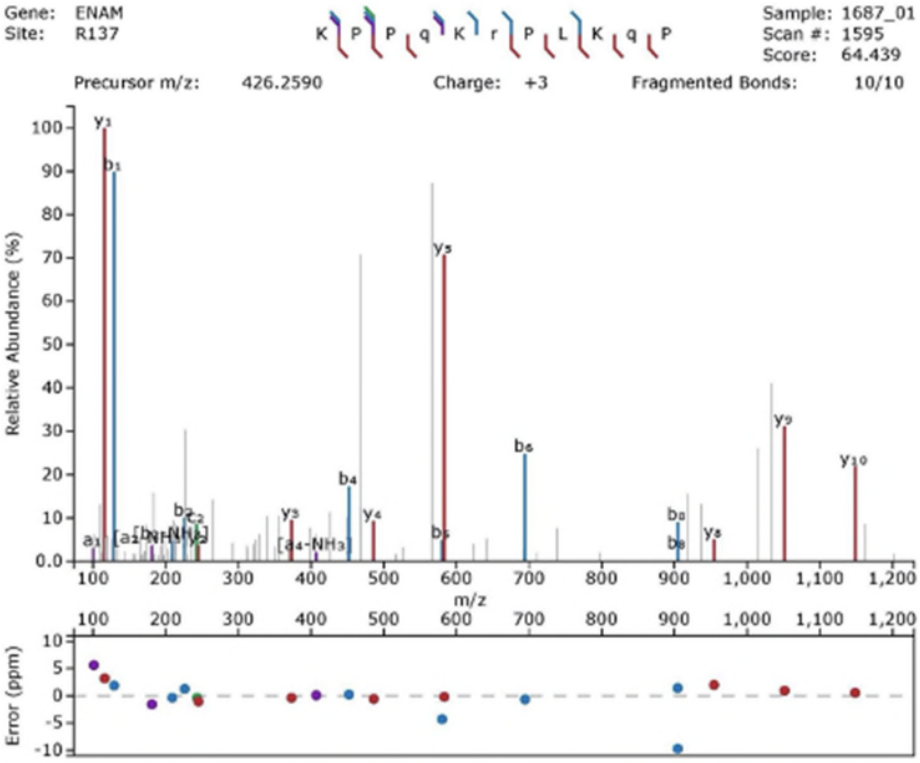
Gene: ENAM
Site: R137
K P P q K r P L K q P
Sample: 1687_01
Scan #: 1595
Score: 64.439
Precursor m/z: 426.2590
Charge: +3
Fragmented Bonds: 10/10
Relative Abundance (%)
m/z
Error (ppm)

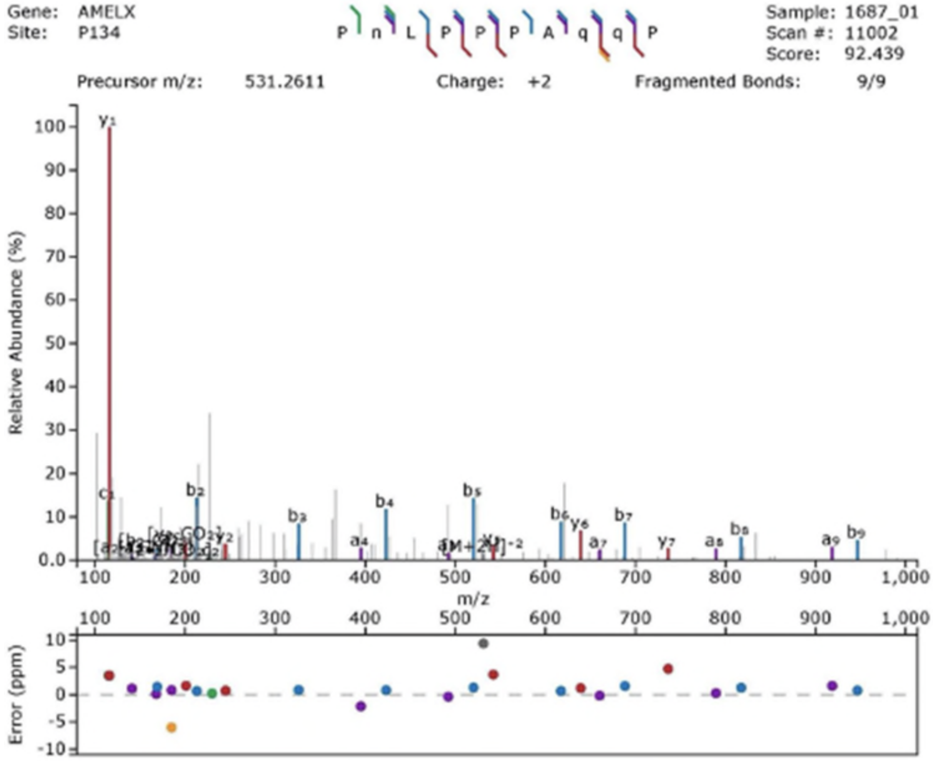
Gene: AMELX
Site: P134
P n L P P P A q q P
Sample: 1687_01
Scan #: 11002
Score: 92.439
Precursor m/z: 531.2611
Charge: +2
Fragmented Bonds: 9/9
Relative Abundance (%)
m/z
Error (ppm)

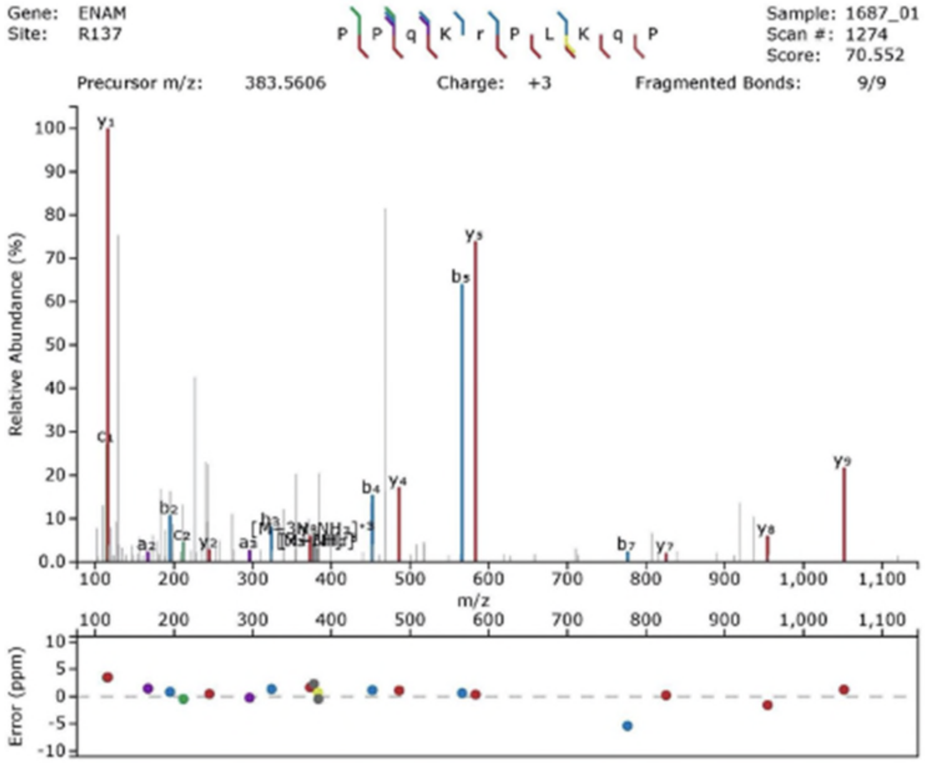
Gene: ENAM
Site: R137
P P q K r P L K q P
Sample: 1687_01
Scan #: 1274
Score: 70.552
Precursor m/z: 383.5606
Charge: +3
Fragmented Bonds: 9/9
Relative Abundance (%)
m/z
Error (ppm)

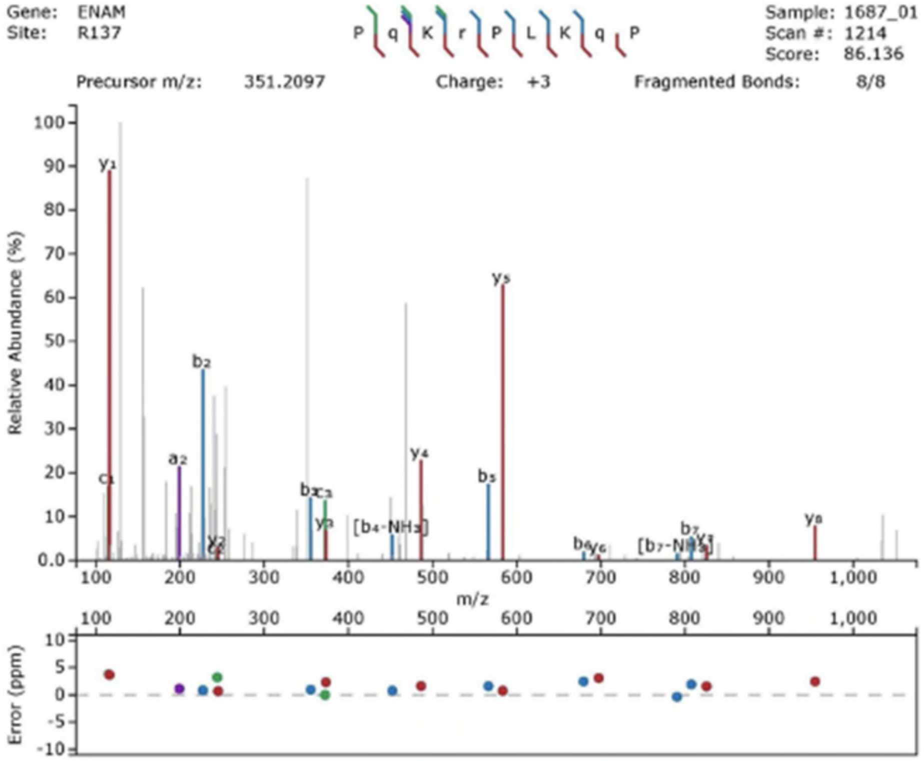
Gene: ENAM
Site: R137
P q K r P L K q P
Sample: 1687_01
Scan #: 1214
Score: 86.136
Precursor m/z: 351.2097
Charge: +3
Fragmented Bonds: 8/8
Relative Abundance (%)
m/z
Error (ppm)

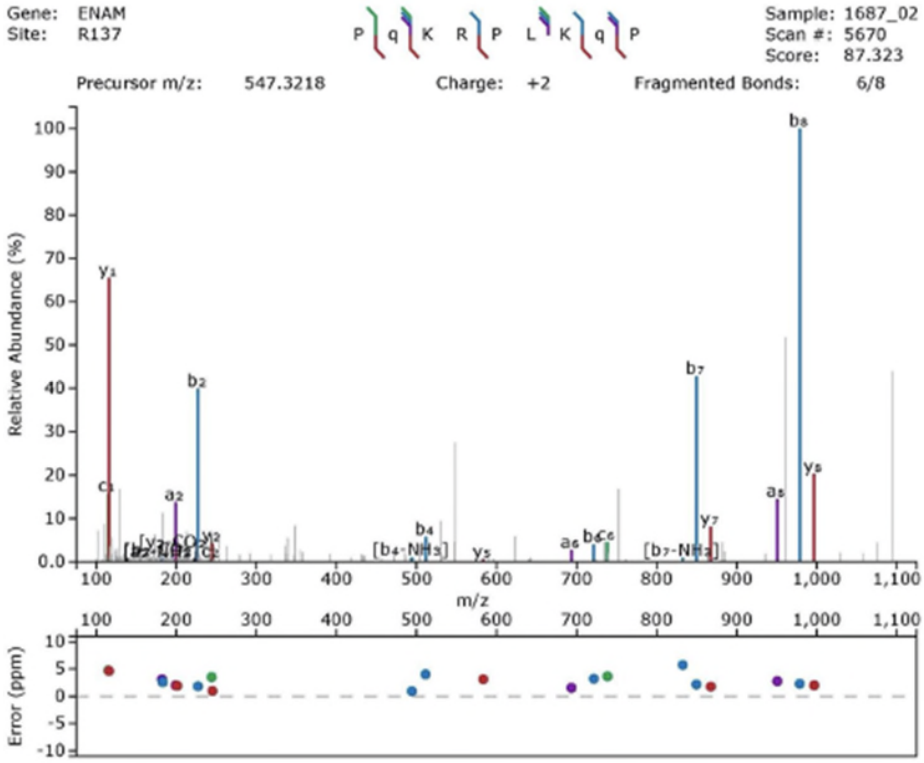
Gene: ENAM
Site: R137
P q K R P L K q P
Sample: 1687_02
Scan #: 5670
Score: 87.323
Precursor m/z: 547.3218
Charge: +2
Fragmented Bonds: 6/8
Relative Abundance (%)
m/z
Error (ppm)

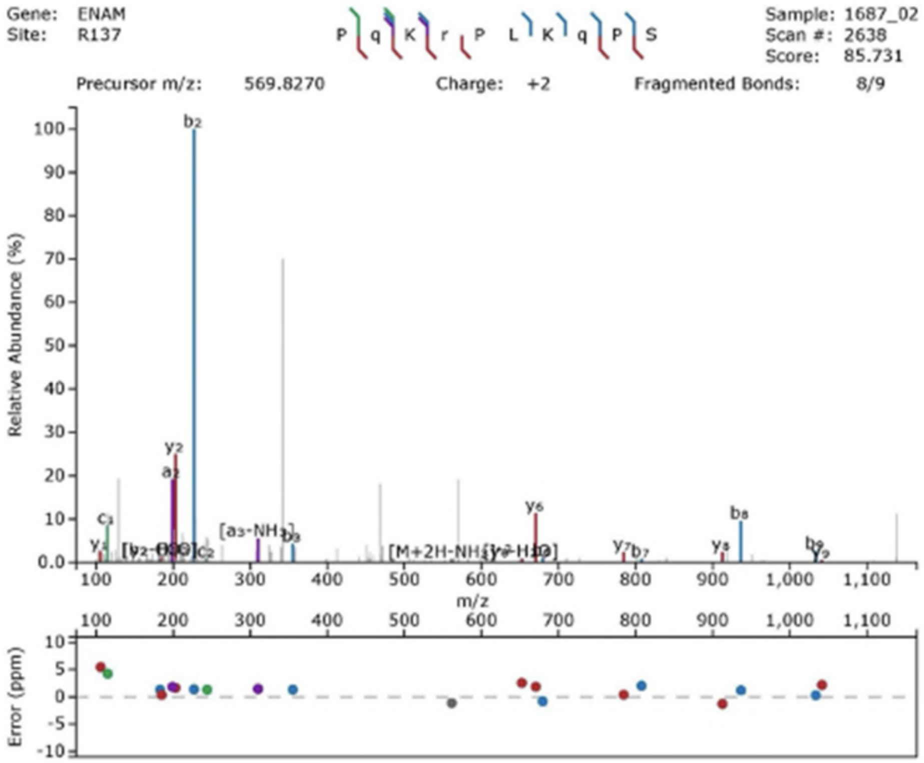
Gene: ENAM
Site: R137
P q K r P L K q P S
Sample: 1687_02
Scan #: 2638
Score: 85.731
Precursor m/z: 569.8270
Charge: +2
Fragmented Bonds: 8/9
Relative Abundance (%)
m/z
Error (ppm)

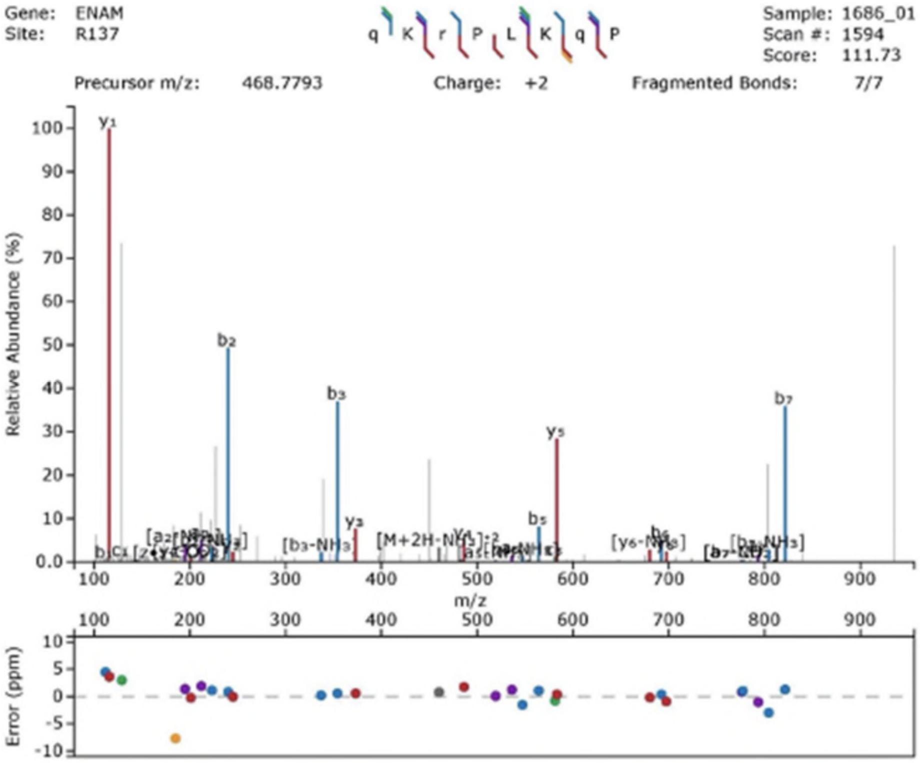

Gene: ENAM
Site: R137
q K r P L K q P
Sample: 1686_01
Scan #: 1594
Score: 111.73
Precursor m/z: 468.7793
Charge: +2
Fragmented Bonds: 7/7
Relative Abundance (%)
m/z
Error (ppm)

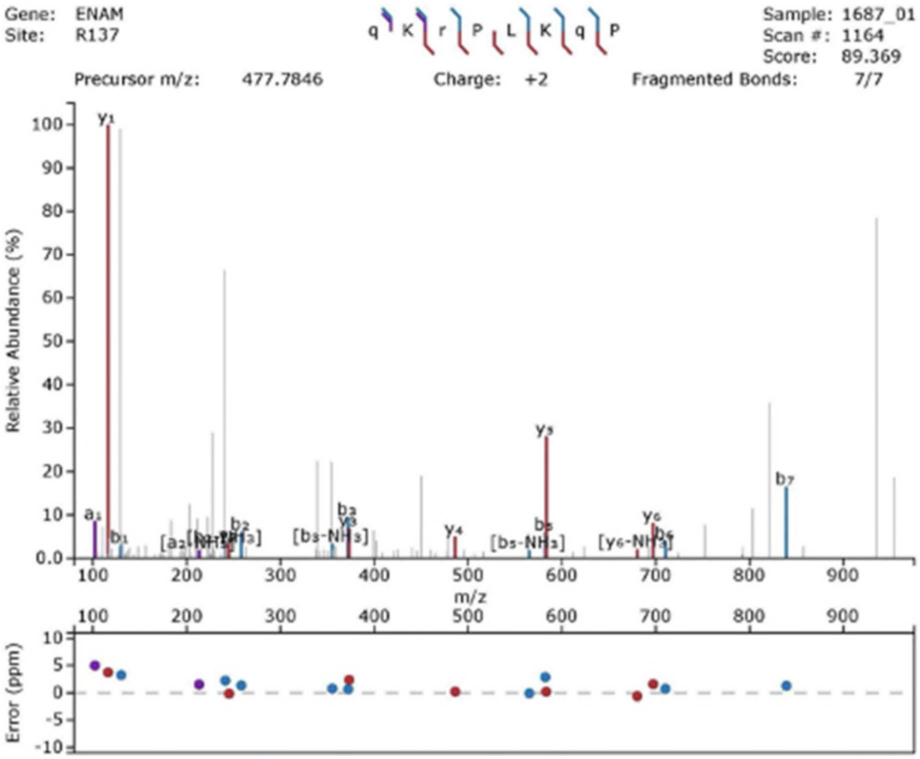

Gene: ENAM
Site: R137
q K r P L K q P
Sample: 1687_01
Scan #: 1164
Score: 89.369
Precursor m/z: 477.7846
Charge: +2
Fragmented Bonds: 7/7
Relative Abundance (%)
m/z
Error (ppm)

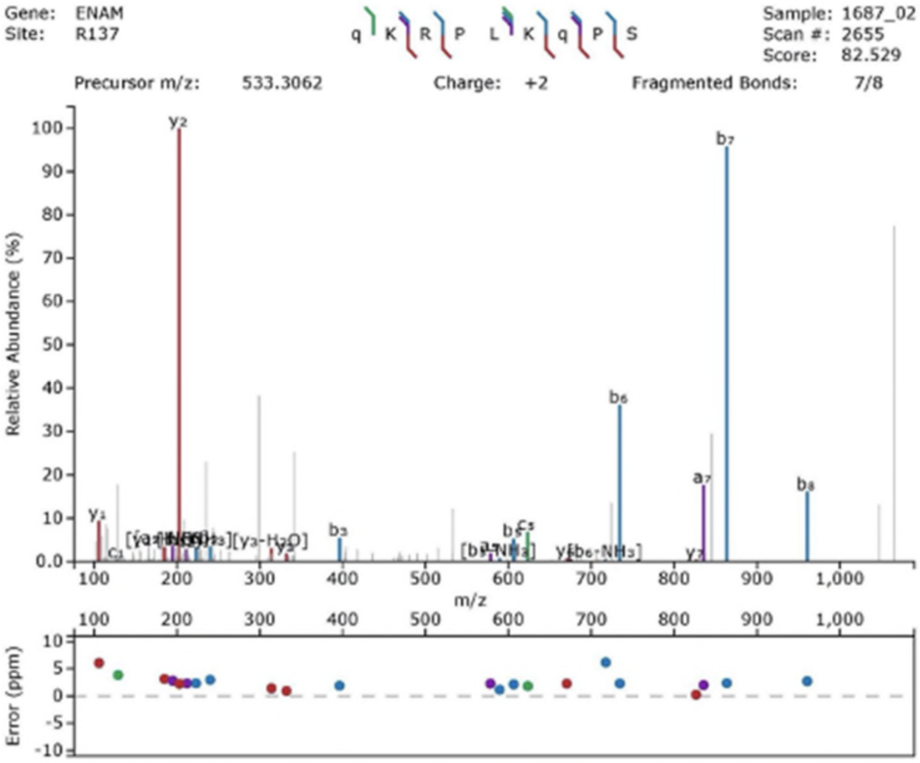
Gene: ENAM
Site: R137
q K R P L K q P S
Sample: 1687_02
Scan #: 2655
Score: 82.529
Precursor m/z: 533.3062
Charge: +2
Fragmented Bonds: 7/8
Relative Abundance (%)
m/z
Error (ppm)
y2
b7
b6
a7
b8
y1
b3
y7

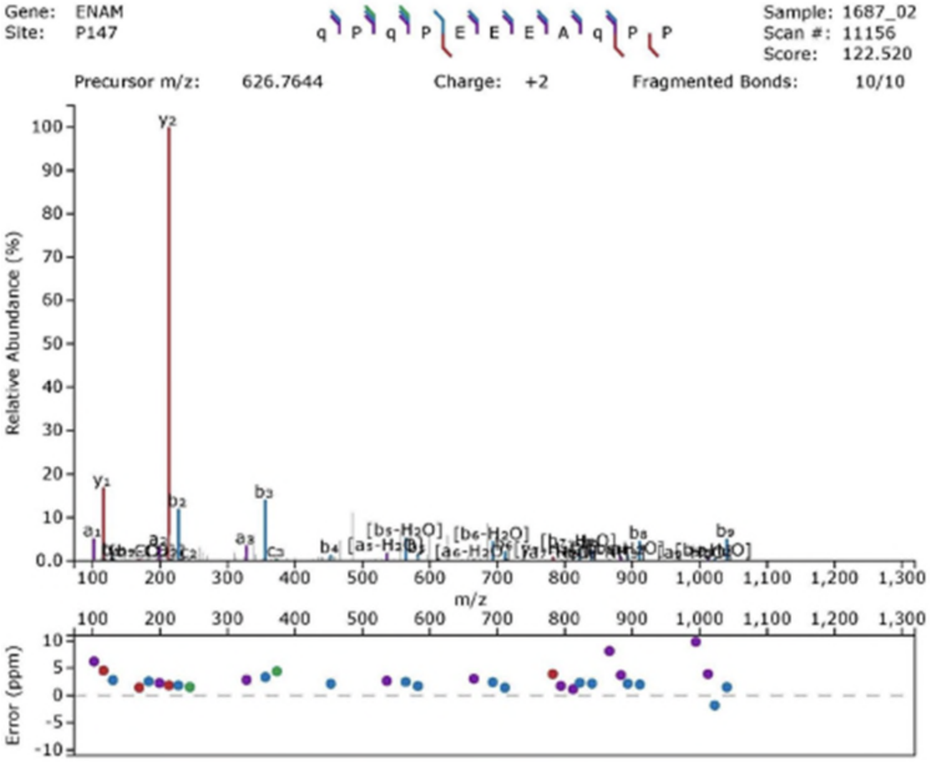
Gene: ENAM
Site: P147
q P q P E E E A q P P
Sample: 1687_02
Scan #: 11156
Score: 122.520
Precursor m/z: 626.7644
Charge: +2
Fragmented Bonds: 10/10
Relative Abundance (%)
m/z
Error (ppm)
y2
y1
b2
b3
a1
a3
b4
b8
b9

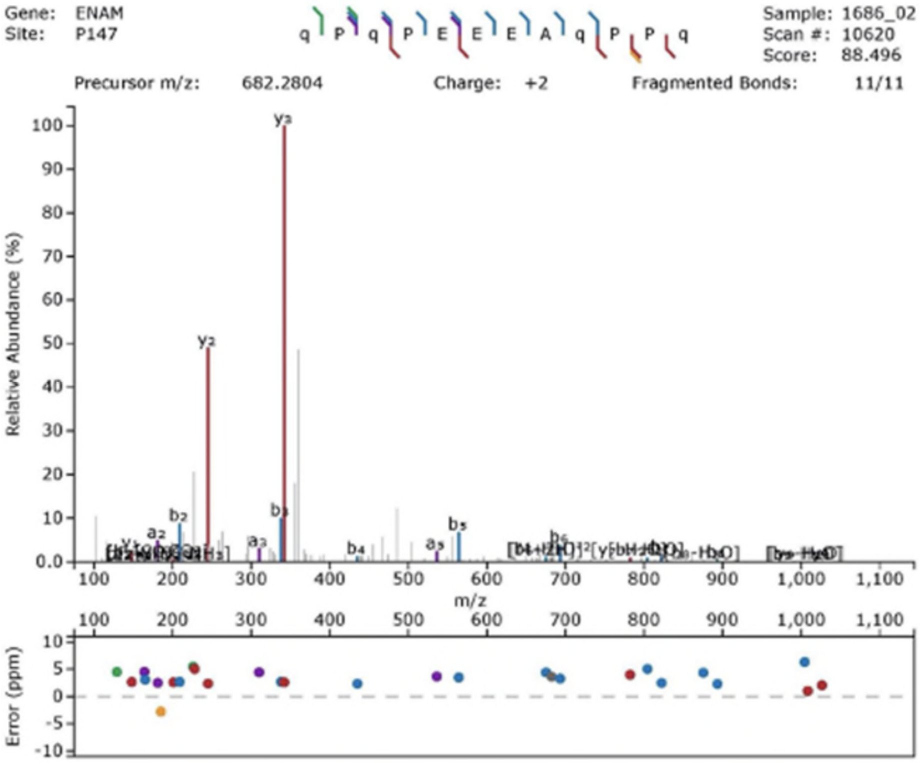
Gene: ENAM
Site: P147
Sample: 1686_02
Scan #: 10620
Score: 88.496
Precursor m/z: 682.2804
Charge: +2
Fragmented Bonds: 11/11
Relative Abundance (%)
m/z
Error (ppm)

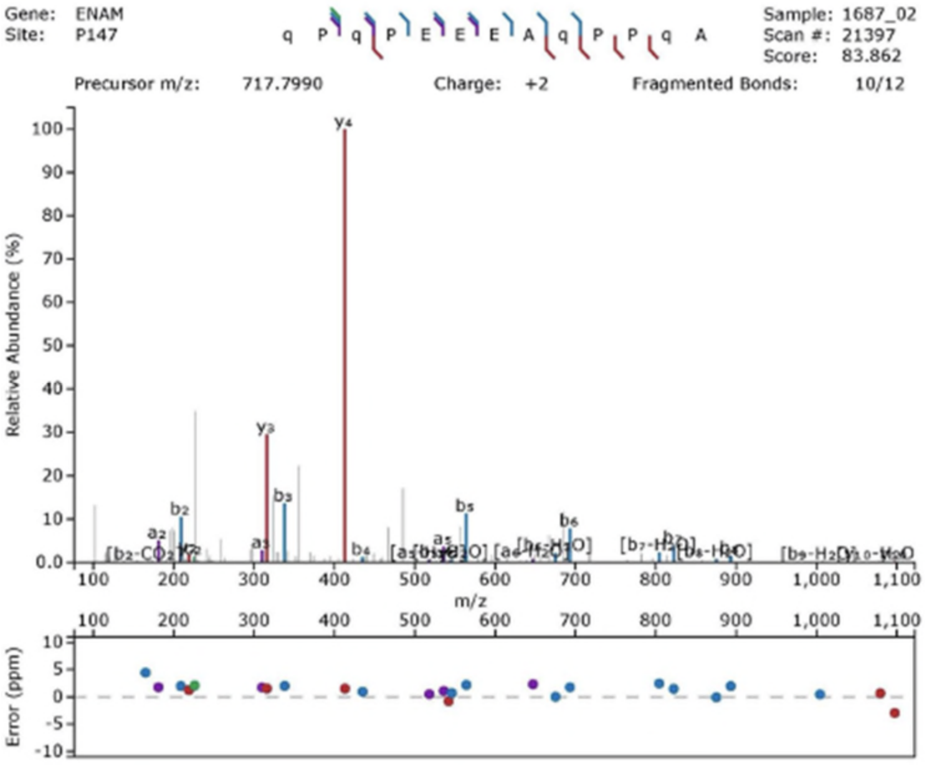
Gene: ENAM
Site: P147
Sample: 1687_02
Scan #: 21397
Score: 83.862
Precursor m/z: 717.7990
Charge: +2
Fragmented Bonds: 10/12
Relative Abundance (%)
m/z
Error (ppm)

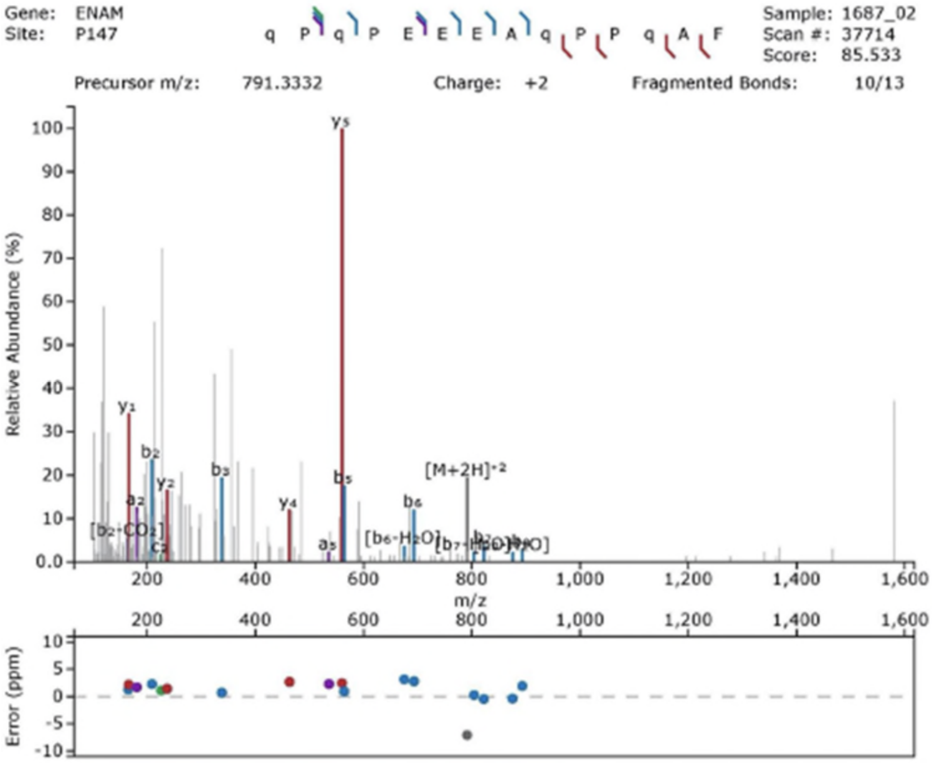
Gene: ENAM
Site: P147
q P q P E E E A q P P q A F
Sample: 1687_02
Scan #: 37714
Score: 85.533
Precursor m/z: 791.3332
Charge: +2
Fragmented Bonds: 10/13
Relative Abundance (%)
m/z
Error (ppm)
[M+2H]+2

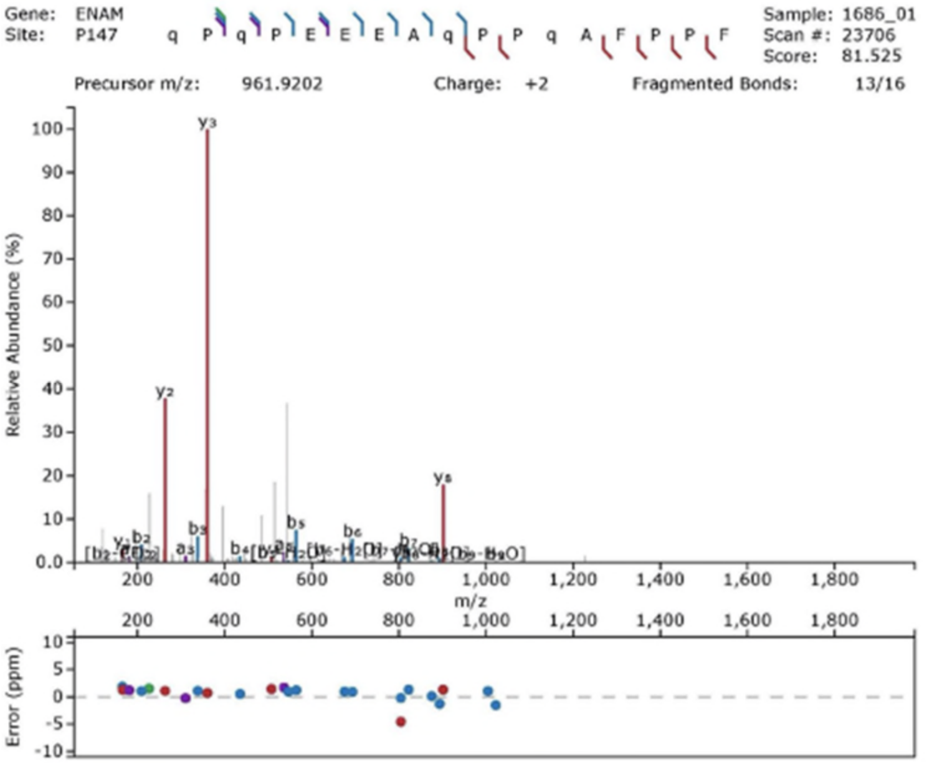
Gene: ENAM
Site: P147
q P q P E E E A q P P q A F P P F
Sample: 1686_01
Scan #: 23706
Score: 81.525
Precursor m/z: 961.9202
Charge: +2
Fragmented Bonds: 13/16
Relative Abundance (%)
m/z
Error (ppm)

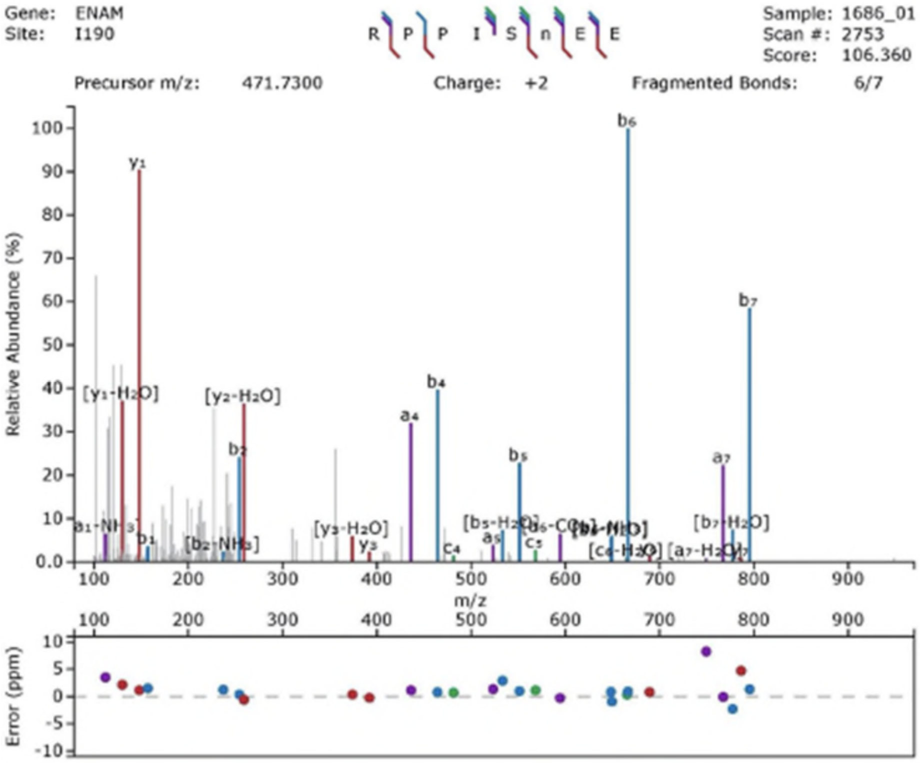
Gene: ENAM
Site: I190
R P P I S n E E
Sample: 1686_01
Scan #: 2753
Score: 106.360
Precursor m/z: 471.7300
Charge: +2
Fragmented Bonds: 6/7
Relative Abundance (%)
m/z
Error (ppm)

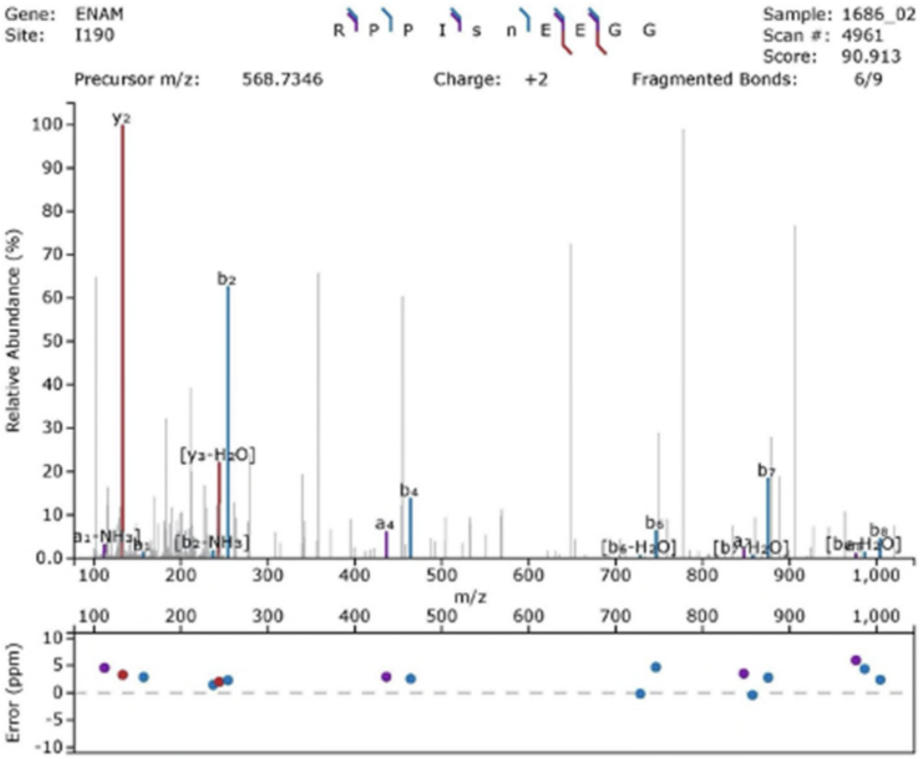
Gene: ENAM
Site: I190
R P P I s n E E G G
Sample: 1686_02
Scan #: 4961
Score: 90.913
Precursor m/z: 568.7346
Charge: +2
Fragmented Bonds: 6/9
Relative Abundance (%)
m/z
Error (ppm)

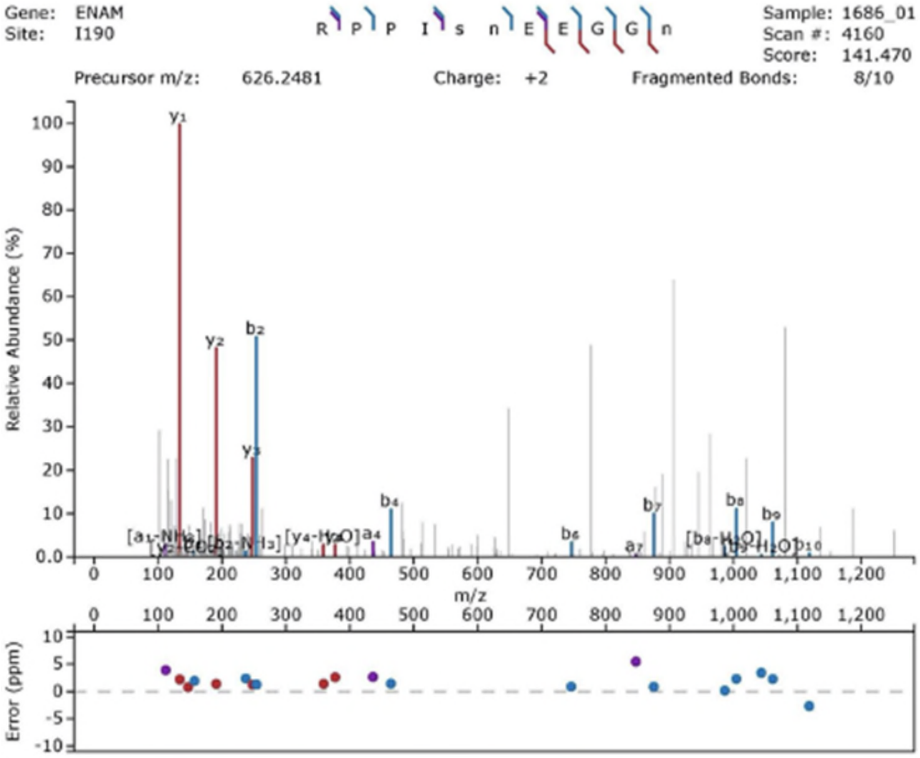
Gene: ENAM
Site: I190
R P P I s n E E G G n
Sample: 1686_01
Scan #: 4160
Score: 141.470
Precursor m/z: 626.2481
Charge: +2
Fragmented Bonds: 8/10
Relative Abundance (%)
m/z
Error (ppm)

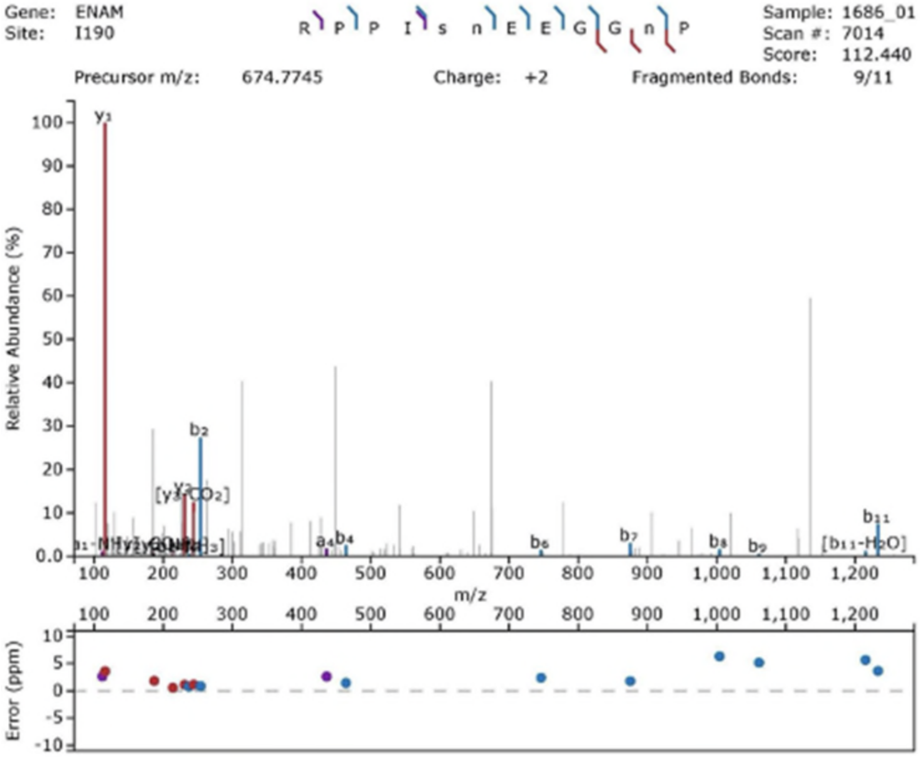
Gene: ENAM
Site: I190
R P P I s n E E G G n P
Sample: 1686_01
Scan #: 7014
Score: 112.440
Precursor m/z: 674.7745
Charge: +2
Fragmented Bonds: 9/11
Relative Abundance (%)
m/z
Error (ppm)

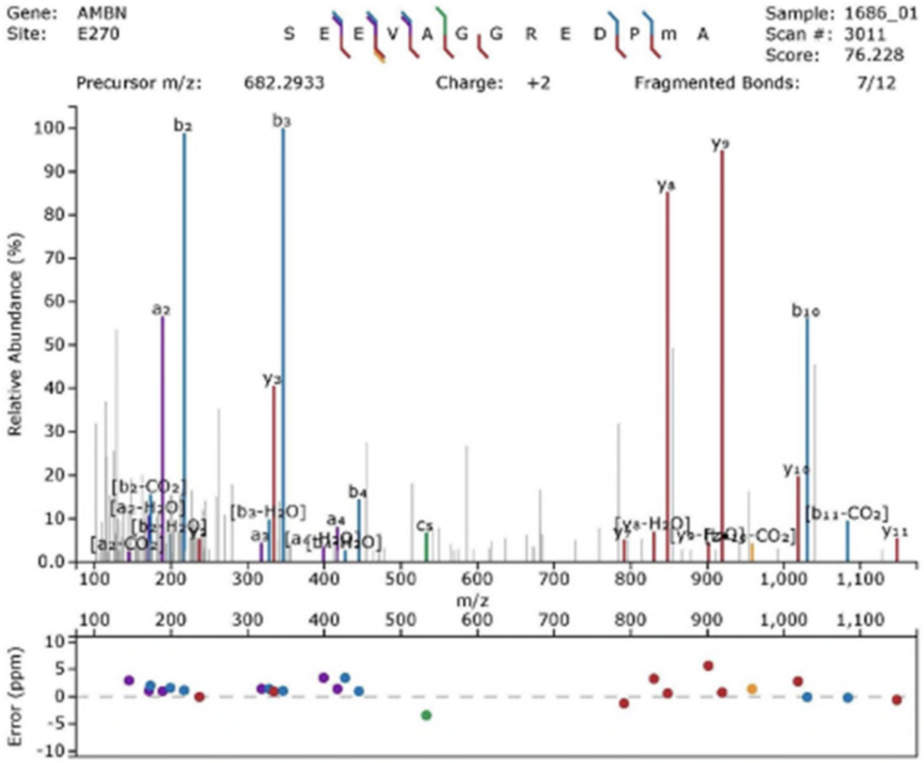
Gene: AMBN
Site: E270
S E E V A G G R E D P m A
Sample: 1686_01
Scan #: 3011
Score: 76.228
Precursor m/z: 682.2933
Charge: +2
Fragmented Bonds: 7/12
Relative Abundance (%)
m/z
Error (ppm)

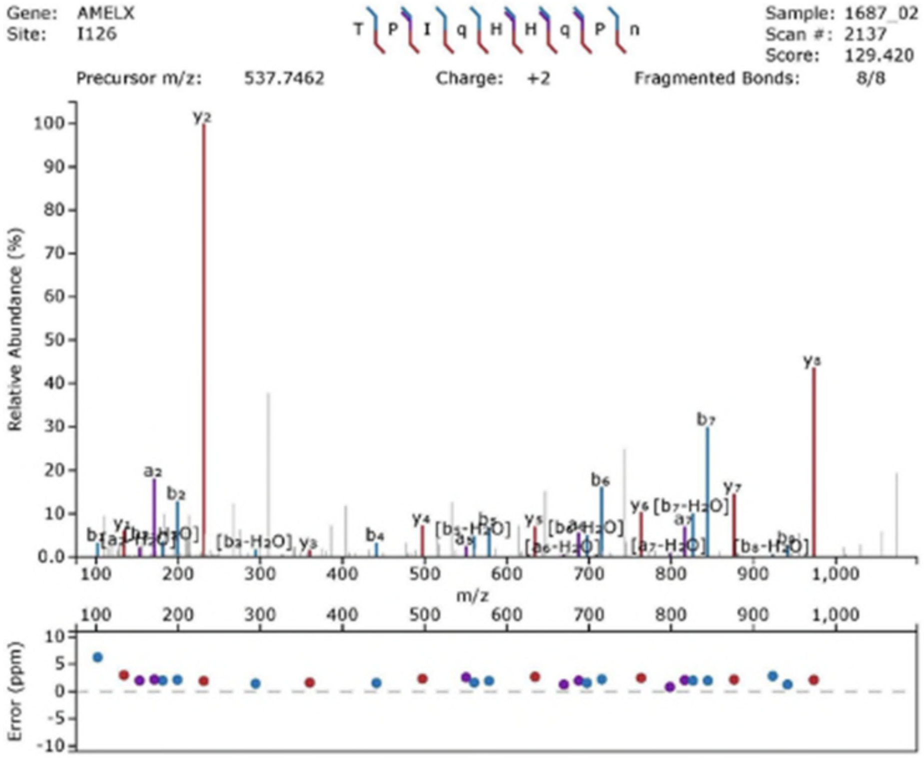
Gene: AMELX
Site: I126
T P I q H H q P n
Sample: 1687_02
Scan #: 2137
Score: 129.420
Precursor m/z: 537.7462
Charge: +2
Fragmented Bonds: 8/8
Relative Abundance (%)
m/z
Error (ppm)

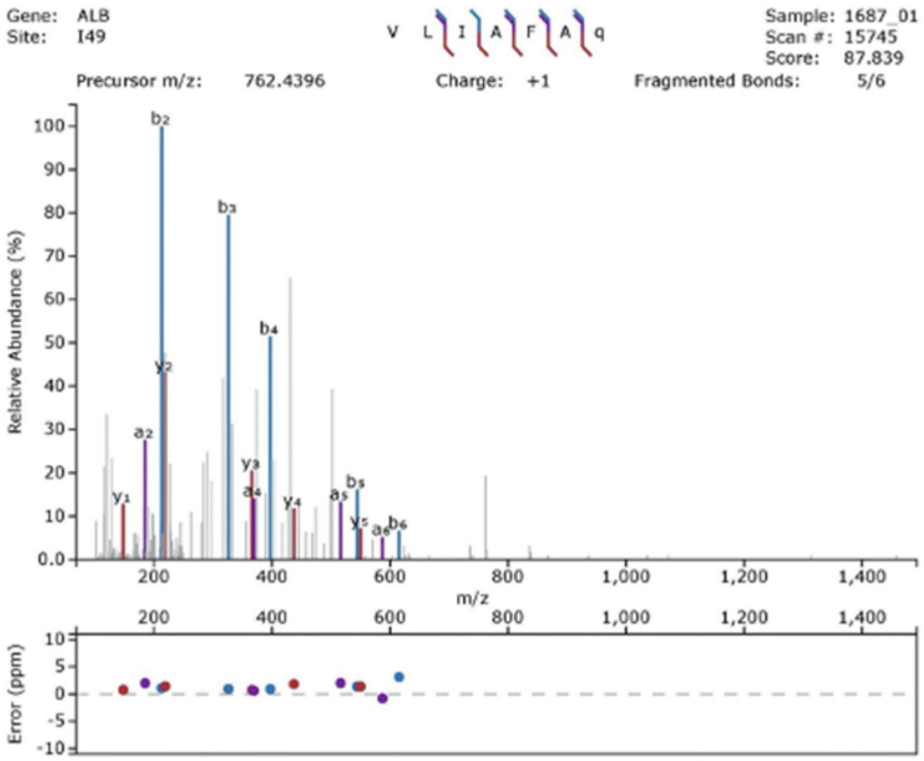
Gene: ALB
Site: I49
V L I A F A q
Sample: 1687_01
Scan #: 15745
Score: 87.839
Precursor m/z: 762.4396
Charge: +1
Fragmented Bonds: 5/6
Relative Abundance (%)
m/z
Error (ppm)

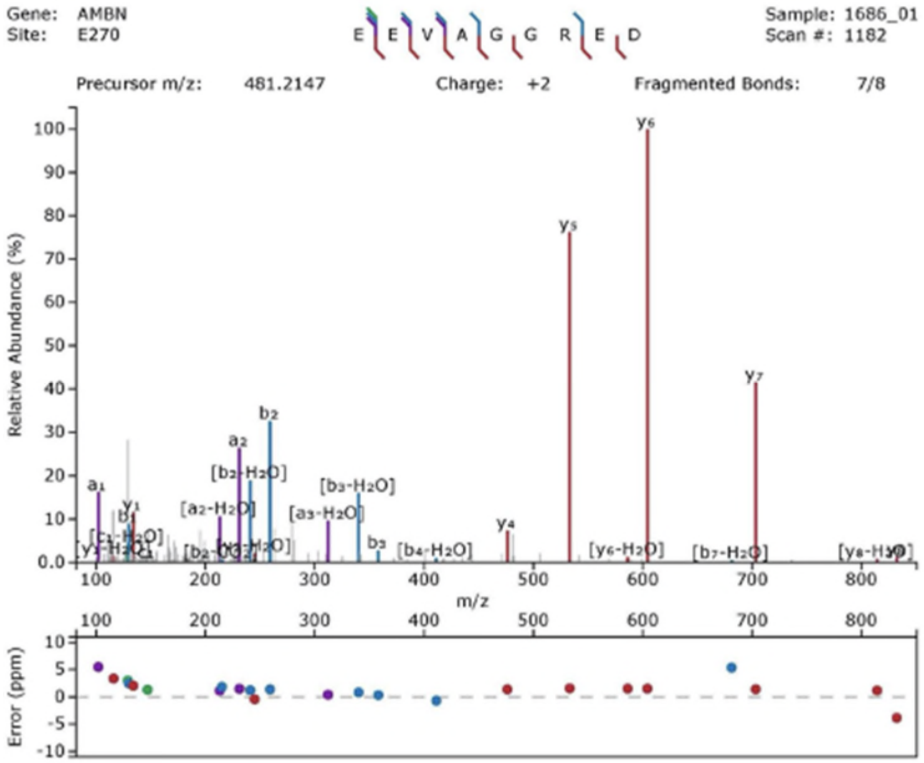
Gene: AMBN
Site: E270
E E V A G G R E D
Sample: 1686_01
Scan #: 1182
Precursor m/z: 481.2147
Charge: +2
Fragmented Bonds: 7/8
Relative Abundance (%)
m/z
Error (ppm)

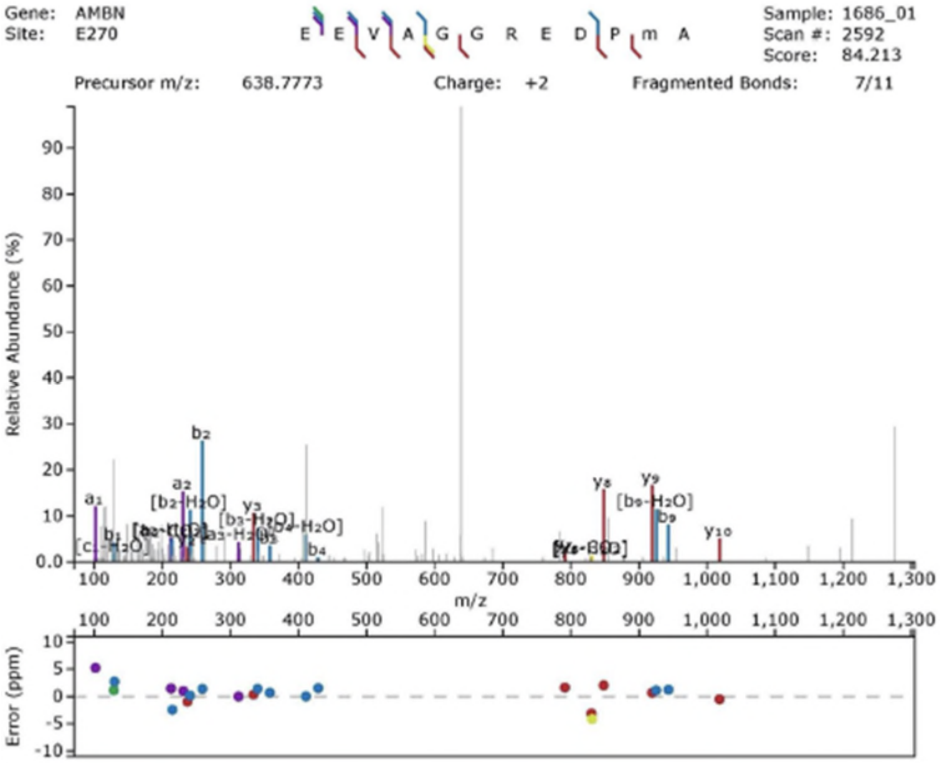
Gene: AMBN
Site: E270
E E V A G G R E D P m A
Sample: 1686_01
Scan #: 2592
Score: 84.213
Precursor m/z: 638.7773
Charge: +2
Fragmented Bonds: 7/11
Relative Abundance (%)
m/z
Error (ppm)

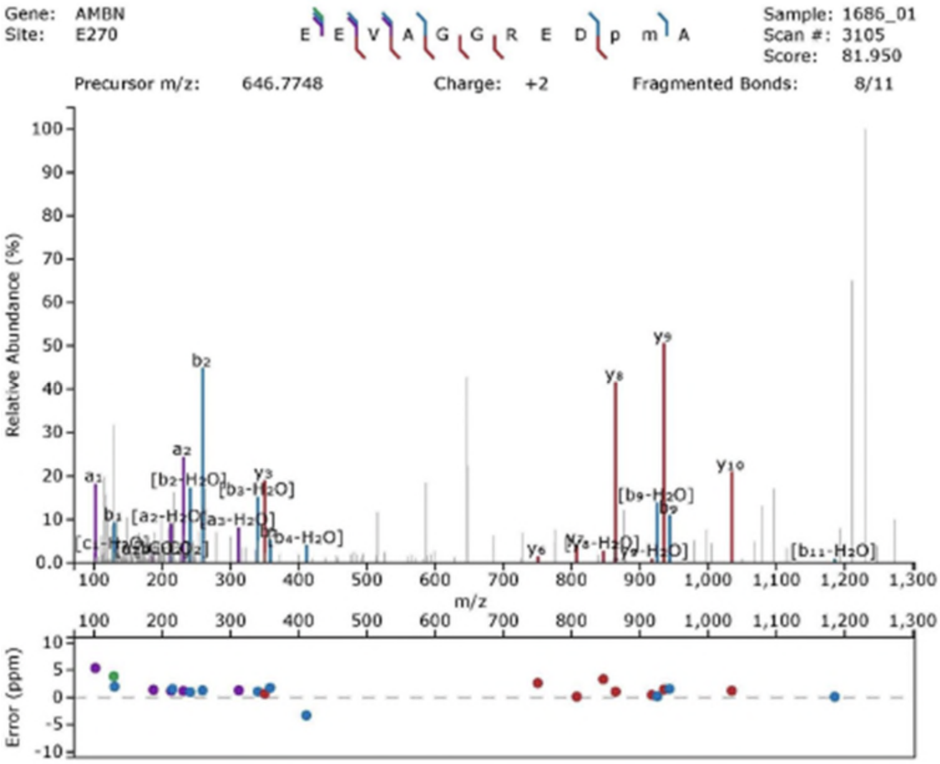
Gene: AMBN
Site: E270
E E V A G G R E D p m A
Sample: 1686_01
Scan #: 3105
Score: 81.950
Precursor m/z: 646.7748
Charge: +2
Fragmented Bonds: 8/11
Relative Abundance (%)
m/z
Error (ppm)

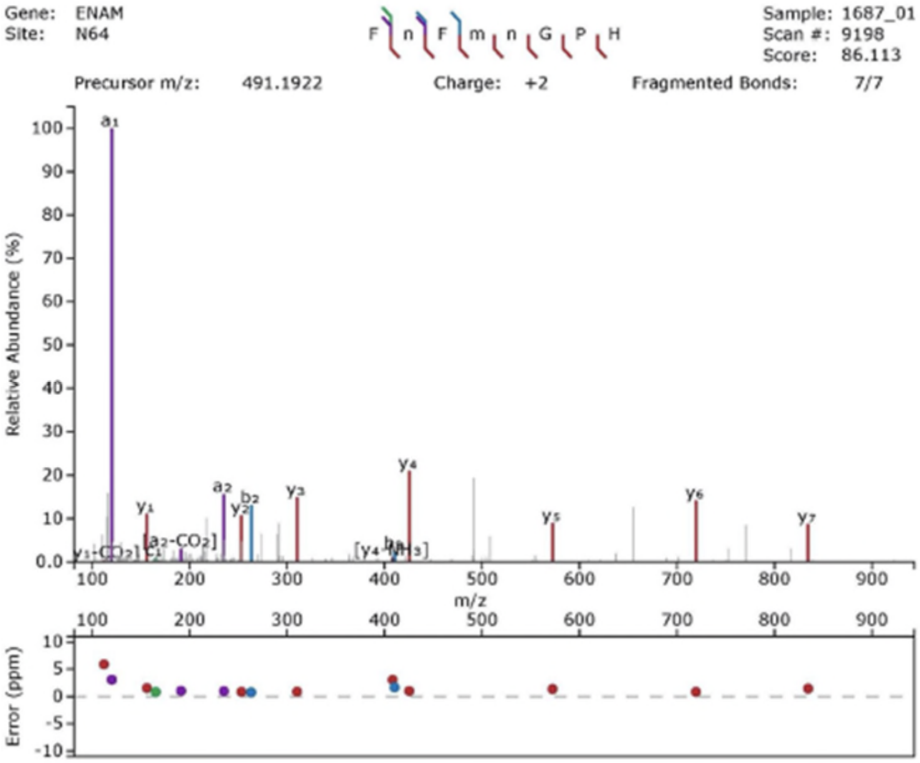
Gene: ENAM
Site: N64
Sample: 1687_01
Scan #: 9198
Score: 86.113
Precursor m/z: 491.1922
Charge: +2
Fragmented Bonds: 7/7
Relative Abundance (%)
m/z
Error (ppm)

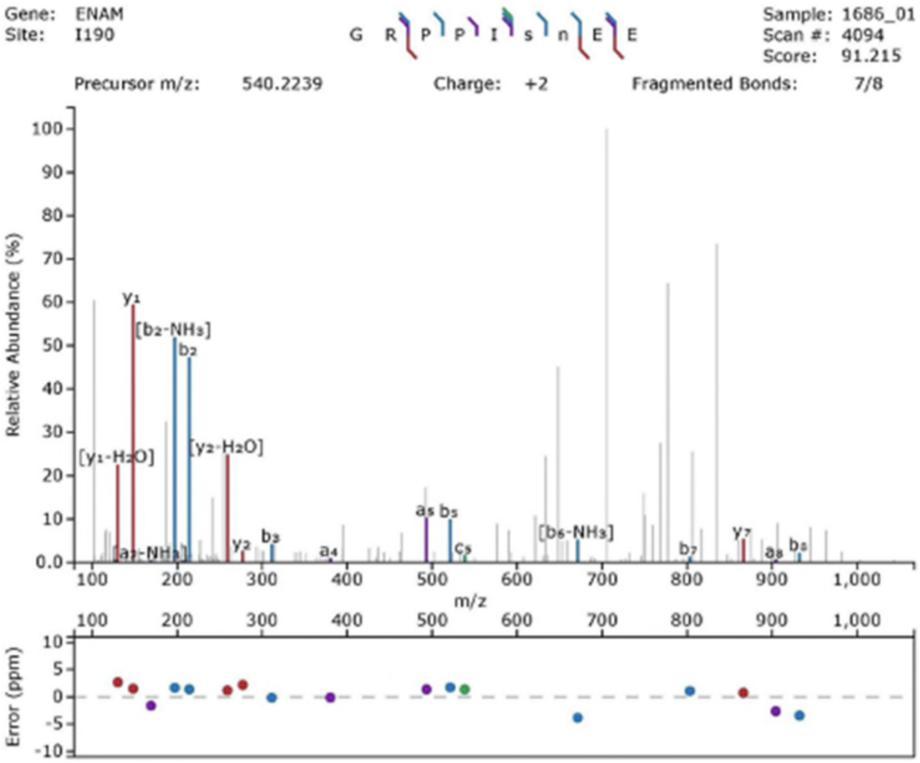
Gene: ENAM
Site: I190
Sample: 1686_01
Scan #: 4094
Score: 91.215
Precursor m/z: 540.2239
Charge: +2
Fragmented Bonds: 7/8
Relative Abundance (%)
m/z
Error (ppm)

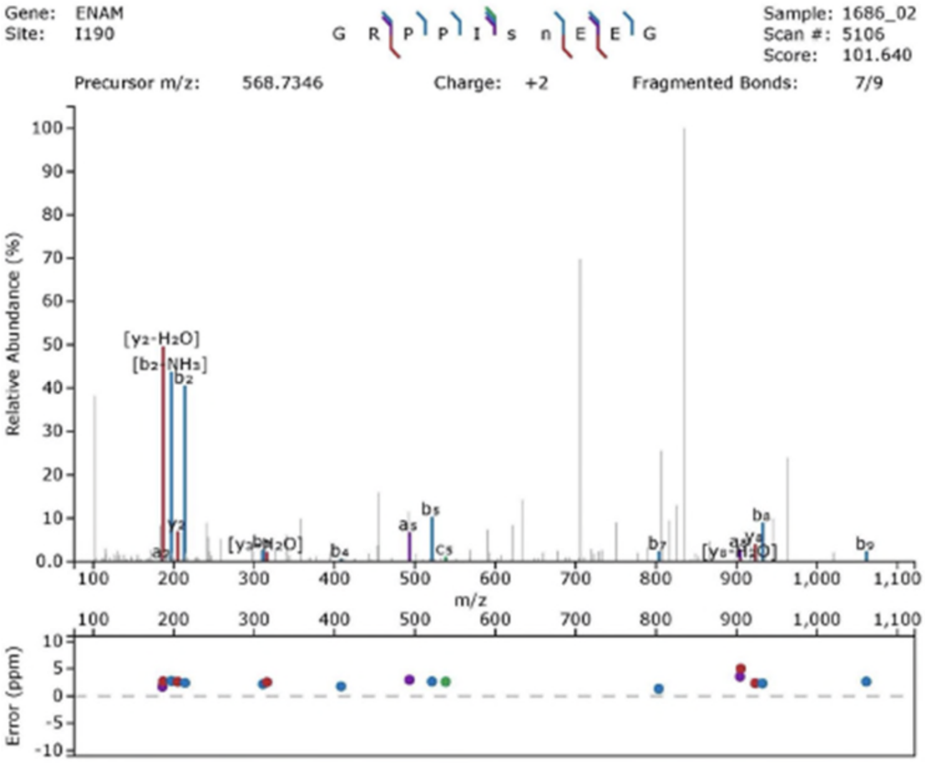
Gene: ENAM
Site: I190
G R P P I s n E E G
Sample: 1686_02
Scan #: 5106
Score: 101.640
Precursor m/z: 568.7346
Charge: +2
Fragmented Bonds: 7/9
Relative Abundance (%)
m/z
Error (ppm)

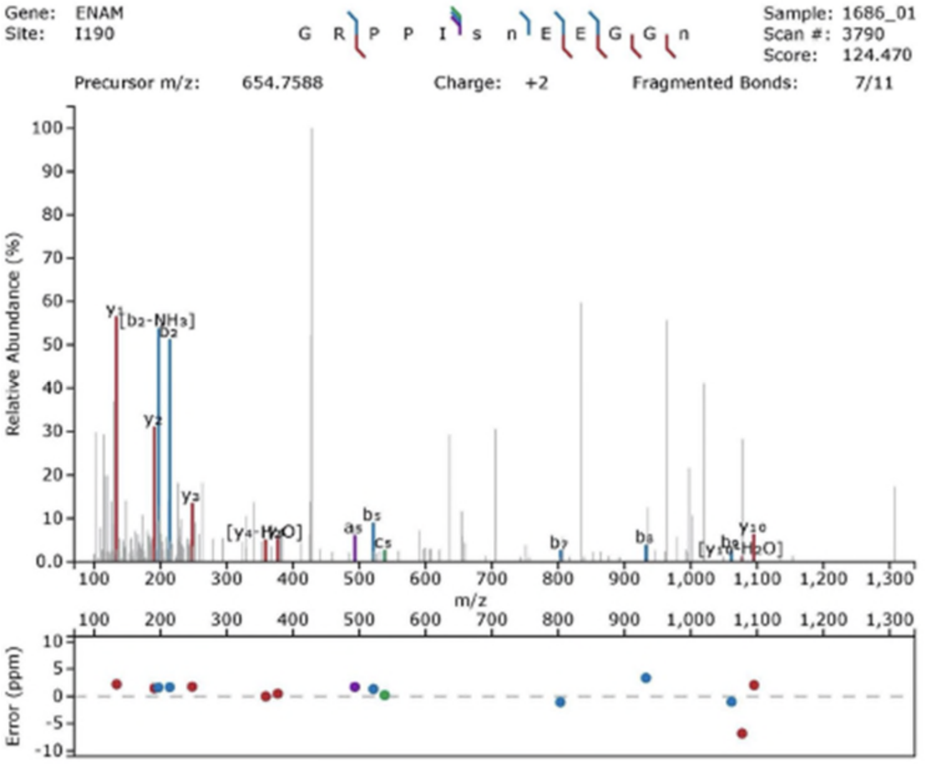
Gene: ENAM
Site: I190
G R P P I s n E E G G n
Sample: 1686_01
Scan #: 3790
Score: 124.470
Precursor m/z: 654.7588
Charge: +2
Fragmented Bonds: 7/11
Relative Abundance (%)
m/z
Error (ppm)
