# Supplementary material for: A Middle Pleistocene Denisovan molar from the Annamite Chain of northern Laos
Source: Nat Commun. 2022 May 17;13:2557. doi: 10.1038/s41467-022-29923-z (PMC9114389; doi:10.1038/s41467-022-29923-z)
Supplement: Supplementary file 6 — Reporting Summary [file 41467_2022_29923_MOESM6_ESM.pdf]

nature portfolio

Corresponding author(s): Fabrice Demeter, Clement Zanolli, Laura Shackelford

Last updated by author(s): YYYY-MM-DD

# Reporting Summary

Nature Portfolio wishes to improve the reproducibility of the work that we publish. This form provides structure for consistency and transparency in reporting. For further information on Nature Portfolio policies, see our Editorial Policies and the Editorial Policy Checklist.

## Statistics

For all statistical analyses, confirm that the following items are present in the figure legend, table legend, main text, or Methods section.

| n/a | Confirmed | |
|---|---|---|
| ☐ | ☒ | The exact sample size ($n$) for each experimental group/condition, given as a discrete number and unit of measurement |
| ☐ | ☒ | A statement on whether measurements were taken from distinct samples or whether the same sample was measured repeatedly |
| ☐ | ☒ | The statistical test(s) used AND whether they are one- or two-sided<br>*Only common tests should be described solely by name; describe more complex techniques in the Methods section.* |
| ☐ | ☒ | A description of all covariates tested |
| ☒ | ☐ | A description of any assumptions or corrections, such as tests of normality and adjustment for multiple comparisons |
| ☐ | ☒ | A full description of the statistical parameters including central tendency (e.g. means) or other basic estimates (e.g. regression coefficient) AND variation (e.g. standard deviation) or associated estimates of uncertainty (e.g. confidence intervals) |
| ☐ | ☒ | For null hypothesis testing, the test statistic (e.g. $F$, $t$, $r$) with confidence intervals, effect sizes, degrees of freedom and $P$ value noted<br>*Give P values as exact values whenever suitable.* |
| ☐ | ☒ | For Bayesian analysis, information on the choice of priors and Markov chain Monte Carlo settings |
| ☒ | ☐ | For hierarchical and complex designs, identification of the appropriate level for tests and full reporting of outcomes |
| ☒ | ☐ | Estimates of effect sizes (e.g. Cohen's $d$, Pearson's $r$), indicating how they were calculated |

*Our web collection on statistics for biologists contains articles on many of the points above.*

## Software and code

Policy information about availability of computer code

| | |
|---|---|
| Data collection | Segmentation and surface generation of the microtomographic data were performed using the software Avizo 8.0 (FEI Visualization Sciences Group). |
| Data analysis | Analyses of tooth structural organization were conducted in Avizo 8.0 (FEI Visualization Sciences Group) and in R v.3.4. The software Deformatica v.4.3 and the R packages ade4 v.1.7-6 and Morpho v.2.7 were used for geometric morphometric analyses.<br>The raw files were analysed with Maxquant version 1.6.0.17.<br>The sequence motif anlaysis of peptides containing a phosphorylated serine residue was done using the R-package "ggseqlogo".<br>Peptide fragment spectra covering sites relevant for phylogenetic placement were annotated using the "Interactive Peptide Spectral Annotator" web tool.<br>Fitting procedures were carried out with the MCDoseE 2.0 software that uses a Bayesian framework approach where the solution is a full probability distribution on the dose equivalent. |

For manuscripts utilizing custom algorithms or software that are central to the research but not yet described in published literature, software must be made available to editors and reviewers. We strongly encourage code deposition in a community repository (e.g. GitHub). See the Nature Portfolio guidelines for submitting code & software for further information.

## Data

Policy information about availability of data

All manuscripts must include a data availability statement. This statement should provide the following information, where applicable:

- Accession codes, unique identifiers, or web links for publicly available datasets
- A description of any restrictions on data availability
- For clinical datasets or third party data, please ensure that the statement adheres to our policy

The mass spectrometry proteomics data generated in this study have been deposited in the in the ProteomeXchange Consortium via the PRIDE partner repository with the dataset identifier PXD018721 [http://proteomecentral.proteomexchange.org/PXD018721], including the used proteomic reference database. The hominins teeth and faunal teeth measurements data generated in this study are provided in the Supplementary Data 2 file. The script for Bayesian modelling the Tam Ngu Hao 2 age estimates is provided in Supplementary Figure 13. TNH2-1 tooth and all the faunal teeth TNH2-2 to TNH2-208 are housed at the National Museum under the responsibility of the Ministry of Information and Culture of Lao PDR. A surface scan of THN2-1 tooth and its EDJ is publicly available in the Human Record archive (https://human-fossil-record.org), register an account and afterwards click the Museum of Lao PDR link on the left hand side of the page). Source data are provided with this paper.

# Field-specific reporting

Please select the one below that is the best fit for your research. If you are not sure, read the appropriate sections before making your selection.

☐ Life sciences ☐ Behavioural & social sciences ☒ Ecological, evolutionary & environmental sciences

For a reference copy of the document with all sections, see nature.com/documents/nr-reporting-summary-flat.pdf

# Ecological, evolutionary & environmental sciences study design

All studies must disclose on these points even when the disclosure is negative.

| | |
|---|---|
| Study description | We report here the first Middle Pleistocene hominin specimen from Laos, with the discovery of a molar from the Tam Ngu Hao 2 (Cobra Cave) limestone cave in the Annamite Mountains. The fossil-bearing breccia ranges between 164-131 kyr based on the Bayesian modelling of luminescence dating of the sedimentary matrix from which it was recovered, U-series dating of an overlying flowstone and U-series–ESR dating of associated faunal teeth. Analyses of the internal structure of the molar in tandem with palaeoproteomic analyses of the enamel indicate that the tooth derives from a young, likely female, Homo individual. The close morphological affinities with the Xiahe specimen from China indicate that they belong to the same taxon and that Tam Ngu Hao 2 most likely represents a Denisovan. |
| Research sample | The lower molar TNH2-1 was compared with a large sample of Early Pleistocene to Holocene Homo specimens. The comparative sample includes molars belonging to Homo erectus, Middle to Late Pleistocene Neanderthals, as well as Late Pleistocene and Holocene modern humans. |
| Sampling strategy | The specimens were selected for their relevance and availability to compare with the newly discovered molar TNH2-1. For palaeoproteomics, the TNH2-1 specimen was sampled in a way to preserve as much as possible some dentine for further Micro CT scan work. Details are given in the Method section of the MS. |
| Data collection | All specimens, including TNH2-1 and comparative data, were scanned by X-ray and neutron microtomography, and synchrotron radiation. Data have been collected by Clément Zanolli, Matthew Skinner and various other colleagues between 2009 and 2022. |
| Timing and spatial scale | The scans were performed between 2009 and 2022 and include fossil and recent Homo specimens from Africa, Europe and Asia. The scans are curated by C. Zanolli. |
| Data exclusions | No available data was excluded from this study. |
| Reproducibility | The methods included in this study have been previously tested and published on numerous occasions and fully demonstrated to be reliable and replicable. Intra- and inter-observer tests revealed differences inferior to 5%. All variables are provided in the SI document which allows full reproducibility. |
| Randomization | Not relevant here as the study is based on a limited fossil sample. Allocation to the groups was based on taxonomic identity of the fossil and extant hominin specimens/samples included in the study. |
| Blinding | No blinding was necessary in this study. Since no trial or biological experiment involving living participants can be conducted, blinding is not applicable to palaeontology/palaeoanthropology. |

Did the study involve field work? ☒ Yes ☐ No

## Field work, collection and transport

| | |
|---|---|
| Field conditions | Field work activities were conducted in the frame of the international paleoanthropological mission at Tam Pa Ling, directed by F. |

| | |
|---|---|
| | Demeter and L. Shackelford. This is an annual fieldword conducted before the rainfall season that comprises the excavation of the main site and the prospecting of new areas surrounding Tam Pa Ling Cave. All of this work is conducted in close collaboration with the Ministry of Information, Culture, and Tourism of Loas PDR and also with the support and help of the villagers from Long Gua Ppa. |
| Location | Tam Ngu Hao 2 (Cobra Cave), Huà Pan province, Laos (20°12’41.5”N, 103°24’32.2”E, altitude 1,116 m. The tower karst in which the cave was formed is positioned on the south-eastern side of P’ou Loi Mountain with an entrance located 34 m above the alluvial plain. |
| Access & import/export | Export permits were officially granted in January 2019 to conduct all the analyses included in the study by the Ministry of Information, Culture and Tourism of Laos PDR. |
| Disturbance | No disturbance to report |

# Reporting for specific materials, systems and methods

We require information from authors about some types of materials, experimental systems and methods used in many studies. Here, indicate whether each material, system or method listed is relevant to your study. If you are not sure if a list item applies to your research, read the appropriate section before selecting a response.

## Materials & experimental systems

| n/a | Involved in the study |
|---|---|
| ☒ | ☐ Antibodies |
| ☒ | ☐ Eukaryotic cell lines |
| ☐ | ☒ Palaeontology and archaeology |
| ☒ | ☐ Animals and other organisms |
| ☒ | ☐ Human research participants |
| ☒ | ☐ Clinical data |
| ☒ | ☐ Dual use research of concern |

## Methods

| n/a | Involved in the study |
|---|---|
| ☒ | ☐ ChIP-seq |
| ☒ | ☐ Flow cytometry |
| ☒ | ☐ MRI-based neuroimaging |

## Palaeontology and Archaeology

| | |
|---|---|
| Specimen provenance | The TNH2-1 molar was recovered in the site of Tam Ngu Hao 2 (Cobra Cave) during the excavation of fossil breccia. Permit to conduct this research had been granted by the MInistry of Information, Culture and Tourism of Laos PDR in December 2018. |
| Specimen deposition | The specimen is curated at the Ministry of Information, Culture and Tourism of Laos PDR.  Sethathirath Road, Vientiane, Laos PDR. |
| Dating methods | The dating methods applied to date the faunal teeth and the sediment are extensively described in the method section of the main text. |

☒ Tick this box to confirm that the raw and calibrated dates are available in the paper or in Supplementary Information.

| | |
|---|---|
| Ethics oversight | No ethical approval or guidance was required as this study was not dealing with modern human nor animal remains. |

Note that full information on the approval of the study protocol must also be provided in the manuscript.
